# Establishment of In Vitro FUS-Associated Familial Amyotrophic Lateral Sclerosis Model Using Human Induced Pluripotent Stem Cells

**DOI:** 10.1016/j.stemcr.2016.02.011

**Published:** 2016-03-17

**Authors:** Naoki Ichiyanagi, Koki Fujimori, Masato Yano, Chikako Ishihara-Fujisaki, Takefumi Sone, Tetsuya Akiyama, Yohei Okada, Wado Akamatsu, Takuya Matsumoto, Mitsuru Ishikawa, Yoshinori Nishimoto, Yasuharu Ishihara, Tetsushi Sakuma, Takashi Yamamoto, Hitomi Tsuiji, Naoki Suzuki, Hitoshi Warita, Masashi Aoki, Hideyuki Okano

**Affiliations:** 1 Department of Physiology, School of Medicine, Keio University, 35 Shinanomachi, Shinjuku-ku, Tokyo 160-8582, Japan; 2 Division of Neurobiology and Anatomy, Graduate School of Medical and Dental Sciences, Niigata University, 1-757, Asahimachidori, Chuo-ku, Niigata 951-8510, Japan; 3 Department of Neurology, Tohoku University Graduate School of Medicine, 1-1 Seiryo-machi, Aoba-ku, Sendai, Miyagi 980-8574, Japan; 4 Department of Neurology, Aichi Medical University School of Medicine, 1-1 Yazako Karimata, Nagakute, Aichi 480-1195, Japan; 5 Center for Genomic and Regenerative Medicine, Graduated School of Medicine, Juntendo University, 2-1-1 Hongo, Bunkyo-ku, Tokyo 113-8421, Japan; 6 Department of Mathematical and Life Sciences, Graduate School of Science, Hiroshima University, 1-3-1 Kagamiyama, Higashihiroshima, Hiroshima 739-8526, Japan; 7 Department of Biomedical Science, Graduate School of Pharmaceutical Sciences, Nagoya City University, 3-1 Tanabe-dori, Mizuho-ku, Nagoya, Aichi 467-8603, Japan

## Abstract

Amyotrophic lateral sclerosis (ALS) is a late-onset motor neuron disorder. Although its neuropathology is well understood, the cellular and molecular mechanisms are yet to be elucidated due to limitations in the currently available human genetic data. In this study, we generated induced pluripotent stem cells (iPSC) from two familial ALS (FALS) patients with a missense mutation in the *fused-in sarcoma* (*FUS*) gene carrying the heterozygous FUS H517D mutation, and isogenic iPSCs with the homozygous FUS H517D mutation by genome editing technology. These cell-derived motor neurons mimicked several neurodegenerative phenotypes including mis-localization of FUS into cytosolic and stress granules under stress conditions, and cellular vulnerability. Moreover, exon array analysis using motor neuron precursor cells (MPCs) combined with CLIP-seq datasets revealed aberrant gene expression and/or splicing pattern in FALS MPCs. These results suggest that iPSC-derived motor neurons are a useful tool for analyzing the pathogenesis of human motor neuron disorders.

## Highlights

• Modeling familial ALS (FALS) using iPSC-derived motor neurons
• Aberrant gene expression and/or splicing in FALS motor neuron precursor cells
• Mis-localization of mutant FUS protein in FALS motor neurons
• Increased apoptosis of FALS motor neurons

## Graphical Abstract

**Figure:**
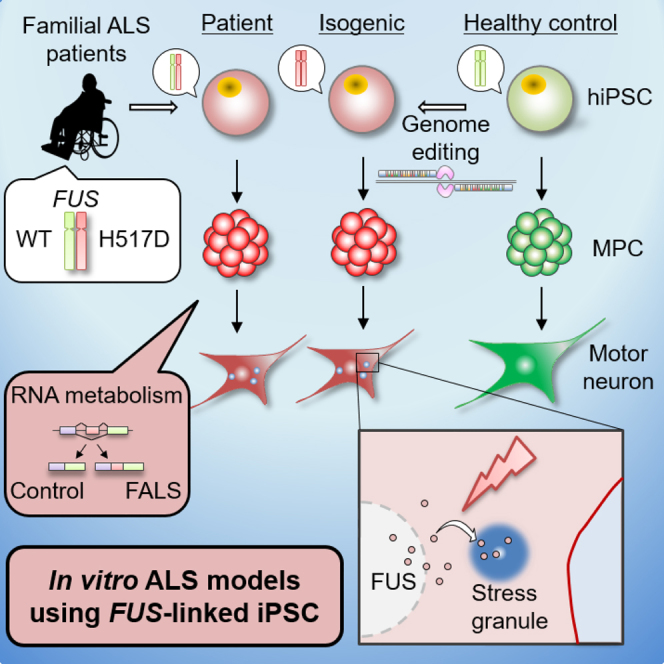

## Teaser

Okano, Yano, and colleagues established iPSCs from two familial ALS patients with the missense mutation in the *FUS* gene and differentiated them into motor neurons as a novel in vitro model for ALS. Moreover, this model enables the pursuit of correlations and new discovery of molecular pathophysiology in various cell biological phenomena by endogenous mutation in patient-derived motor neurons.

## Introduction

Amyotrophic lateral sclerosis (ALS) is a neurodegenerative disease resulting in the selective death of motor neurons (Cleveland and Rothstein, 2001). ALS symptoms are associated with muscle weakness and paralysis and approximately 80% of ALS patients die within 3–5 years after the onset of these symptoms. The prevalence of ALS is two per 100,000 people per year (Bruijn et al., 2004) and approximately 10% of patients have a familial history of the disease (Gros-Louis et al., 2006). Familial ALS (FALS) is identified by mutations in several genes, including *SOD1*, *TARDBP* and *FUS* (Chen et al., 2013).

Several efforts including animal and in vitro culture models have been undertaken to understand the pathogenic mechanism of ALS. In animal models, neurobiological phenotypes of ALS are observed, which are due to multiple pathogenic mechanisms, including protein degradation, oxidative stress, inflammation, paraspeckle formation, mitochondrial dysfunction and apoptotic pathways (Lanson and Pandey, 2012, Nishimoto et al., 2013, Robberecht and Philips, 2013, Tsao et al., 2012). In addition, the use of recently developed induced pluripotent stem cell (iPSC) technologies also enables understanding of the disease pathogenesis (Mattis and Svendsen, 2011, Okano and Yamanaka, 2014). Indeed, iPSCs have been generated from ALS patients with mutations in SOD1 (Chestkov and Vasilieva, 2014, Dimos et al., 2008), TDP-43 (Bilican et al., 2012, Egawa et al., 2012), C9ORF72 (Almeida et al., 2013, Sareen et al., 2013) and recent publications of FUS (Lenzi et al., 2015, Liu et al., 2015, Di Salvio et al., 2015) suggest a useful tool for pursuing the cellular pathogenesis and mechanism underlying FALS.

FUS, also known as Translocated in Liposarcoma (TLS), is a DNA/RNA-binding protein containing a glycine-rich region, an RNA recognition motif and a nuclear localization signal (Lattante et al., 2013, Yang et al., 2010). In FALS, more than 50 mutations in the *FUS* gene have been reported (Lattante et al., 2013). Some mutant FUS proteins form nuclear/cytosolic protein aggregations that shift from the nucleus to the cytoplasm (Dormann et al., 2010, Suzuki et al., 2012, Vance et al., 2013, Zhou et al., 2013). This sequestration of FUS into aggregations is thought to be a potential cue for the initiation of motor neuron degeneration.

In the present study, we generated iPSCs from two ALS patients carrying the FUS H517D mutation, healthy volunteers and isogenic iPSCs with FUS H517D mutation using the TALEN system and investigated the multifaceted cellular phenotypes of their motor neurons in vitro. We differentiated these iPSCs into the motor neuron cell lineage and observed significant mis-localization of mutant FUS to the cytosol and the accumulation of FUS in stress granules under various stresses, which has never been reported for wild-type FUS protein. Furthermore, using two comprehensive analyses, exon array combined with previous CLIP-seq data (Lagier-Tourenne et al., 2012), we identified transcripts showing aberrant gene expression, which may be involved in FUS-dependent pathology, even in motor neuron precursor cells (MPCs) derived from FALS iPSCs. The subsequent analysis using the IN Cell Analyzer revealed an increase in neuronal cell death and a decrease in neurite length in FALS iPSC-derived HB9-positive motor neurons compared with controls under stress conditions. In contrast, in our analysis on βIII-TUBULIN-positive neurons, there were nearly no changes in FUS mis-localization, FUS accumulation in stress granules, occurrence of neuronal cell death, or neurite length. Thus, the present report describes the in vitro modeling of human ALS with the *FUS* mutation to uncover the pathogenetic history of this disease.

## Results

### Generation of FUS-ALS-iPSCs and Differentiation into Motor Neurons

**Figure 1:**
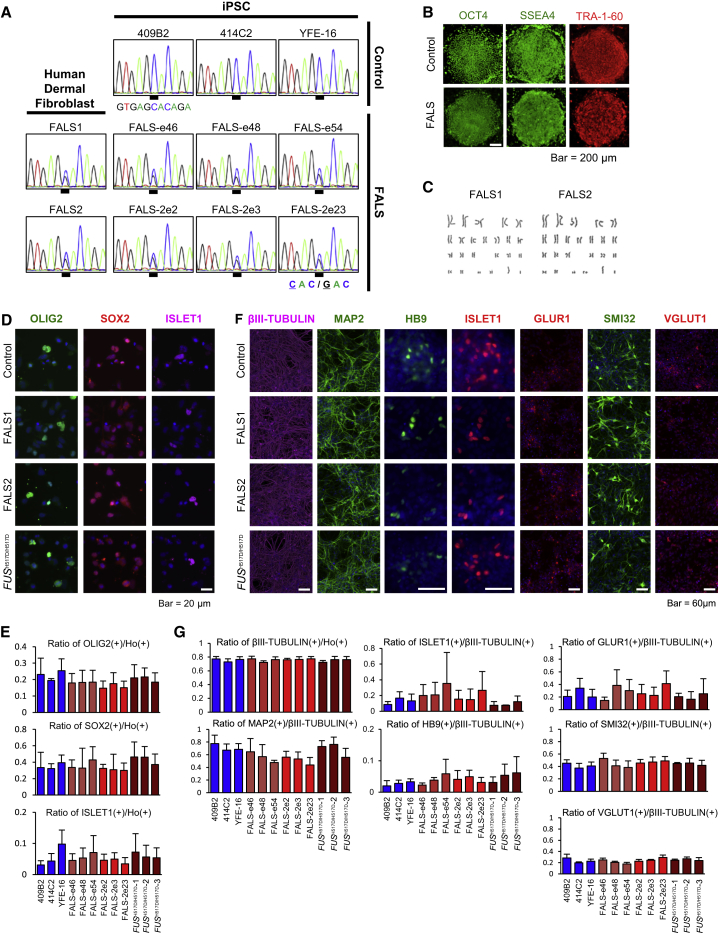
Characterization of iPSCs and Differentiation into Motor Neurons (A) Human dermal fibroblasts from two ALS patients who carried the FUS H517D heterozygous mutation (C-to-G heterozygous mutation); the mutation was maintained in the generated iPSCs. (B) Representative image of immunochemical analysis of pluripotent markers, OCT4, SSEA4 and TRA-1-60. Control, YFE-16; FALS, FALS-2e2. The same images are shown in Figure S1B. The scale bar represents 200 μm. (C) Representative karyotypes of the generated FALS1 and FALS2 iPSC lines are shown. (D) Representative image of immunocytochemistry for the neural stem cell marker (SOX2) and motor neuron progenitor markers (OLIG2 and ISLET1). The scale bar represents 20 μm. (E) Quantitative data of the ratio of each MPC marker-positive cell/Hoechst-positive cell (n = 3 independent experiments; means ± SD; Tukey's test). (F) Representative image of immunocytochemistry for motor neuron markers (HB9, ISLET1 and SMI32) and other neural markers (βIII-TUBULIN, MAP2, VGLUT1 and GLUR1). The scale bars represent 60 μm. (G) Quantitative data of the ratio of each marker-positive cell/βIII-TUBULIN-positive cell (n = 3 independent experiments; mean ± SD; Tukey's test).

We generated FALS iPSCs derived from skin fibroblasts isolated from two FALS patients (Figure S1A and Table S1), both with a point mutation in the gene encoding the C-terminal domain of the FUS protein (Akiyama et al., 2016). To reprogram the skin cells into FUS-ALS-iPSCs, we used episomal vectors carrying *OCT4*, *SOX2*, *KLF4*, *L-MYC*, *LIN28* and *p53* shRNA. The two ALS patients had a heterozygous C-to-G transition at nucleotide 1,550 (c.1550C>G) in the exon 15 coding sequence of the *FUS* gene (hereafter referred to as patient-1, FALS-e46, FALS-e48, and FALS-e54; and patient-2, FALS-2e2, FALS-2e3, and FALS-2e23). We confirmed that FALS iPSCs also harbored the heterozygous C-to-G point mutation in the *FUS* gene (Figure 1A) and had no other mutations in the *FUS* gene (data not shown). This point mutation causes a single amino acid substitution (histidine to aspartic acid) at amino acid position 517 in the C-terminal domain of the FUS protein, termed H517D. Furthermore, we established another control iPSC line, YFE-16 (Shimojo et al., 2015), in addition to the two control human iPSC lines, 409B2 and 414C2, which were established previously (Table S1) (Okita et al., 2011). These three control lines were derived from two individuals who were diagnosed as clinically healthy and did not have the C-to-G point mutation in the *FUS* gene (Figure 1A). Importantly, these iPSC lines showed the typical morphology of colonies similar to human embryonic stem cell lines, based on the expression of pluripotent stem cell markers (SSEA4, OCT4, and TRA-1-60) by immunocytochemical analysis (Figures 1B and S1B; control and FALS in Figure 1B represented YFE-16 and FALS-2e2 respectively and the same images are shown in Figure S1B), normal karyotypes by the G-band staining method (Figure 1C) and no exogenous transgene expression by qRT-PCR (Figure S1C) or *oriP* genomic PCR (Figure S1D). We also confirmed the pluripotency of differentiation potentials into three germ layers (Figure S1E). In addition, we generated isogenic iPSCs with FUS H517D homozygous mutation (hereafter referred to as *FUS*^H517D/H517D^-1, -2, and -3) using the TALEN genome editing systems on 409B2 control iPSCs (Figure S2).

All iPSCs were differentiated into neural lineages including motor neurons based on a previously described protocol but with slight modifications (Bilican et al., 2012, Chambers and Fasano, 2009, Egawa et al., 2012, Hester et al., 2011, Hu and Zhang, 2009, Imaizumi et al., 2015, Nizzardo et al., 2010, Surmacz et al., 2012, Matsumoto et al., 2016). The iPSCs were transferred into a suspension culture to form the neurosphere in neural progenitor maintenance media containing retinoic acid and the hedgehog signaling activator, purmorphamine, to promote the commitment of MPCs. All MPCs were constructed from motor neuron progenitor marker, OLIG2 and SOX2 double-positive cells, and an early marker of motor neuron differentiation, ISLET1 positive cells (Figure 1D). Quantitative analysis for the ratio of marker expression using IN Cell Analyzer revealed that there was no significant difference in the differentiation ratio between control, FALS, and *FUS*^H517D/H517D^ MPCs (Figure 1E). MPCs were passaged twice and then adhered on poly-ornithine/laminin-coated plates. Neurons were differentiated after 20 days in adherent culture. These neuronal cell populations contained motor neurons (i.e., HB9, ISLET1 and SMI32), glutamatergic neurons (i.e., VGLUT1) and glutamate-responsive neurons (i.e., GLUR1) (Figure 1F) and there was no significant difference in the ratio of these markers between control, FALS, and *FUS*^H517D/H517D^ neurons (Figure 1G).

### Aberrant Gene Expression in FALS Motor Neuron Progenitor Cells

**Figure 2:**
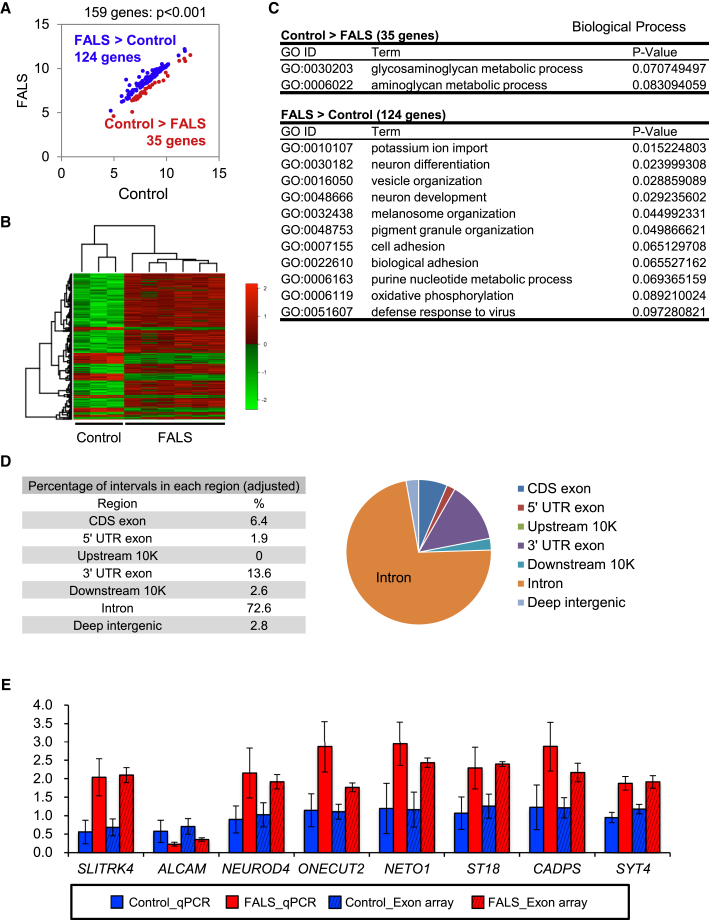
Exon Array Analysis Using MPCs and Comparison with FUS CLIP-Seq (A) Scatterplot analysis of gene expression using control and FALS MPCs. A total of 124 genes were upregulated (blue) and 35 were downregulated (red) in FALS MPCs compared with control MPCs. (B) The heatmap of correlation coefficients. (C) Major GO terms showed both increases and decreases in gene expression in FALS versus control MPCs. (D) Reanalysis of previously reported CLIP-seq (Lagier-Tourenne et al., 2012). (E) Quantitative RT-PCR analysis of the expression levels for eight randomly selected genes in control and FALS iPSC-derived MPCs. Solid and hatched bars show qRT-PCR and exon array data, respectively (n = 3–6 independent samples; mean ± SD; Dunnett's test).

We performed exon array analysis using an Affymetrix GeneChip Human Exon 1.0 ST Array to analyze RNA profiles in control and FALS-derived MPCs, which efficiently differentiate into motor neurons. We compared 18,738 genes using core probes to observe fold changes (FCs) in gene expression between the three control and six FALS-derived cell types. A scatterplot of FCs showed a very high correlation coefficient of more than 0.9, suggesting our in vitro culture model was stable regarding their transcriptome. Next, we prepared a list of FUS-regulated genes, which were differentially expressed for each cell, by filtering the gene-level signal intensities with Bayes statistics p values <0.001. Of the 159 differently expressed genes we identified, 124 genes were upregulated and 35 genes were downregulated in FALS MPCs as summarized in plots and a heatmap (Figures 2A and 2B). We analyzed the Gene Ontology (GO) terms (Figure 2C) of the genes, including *SLITRK4*, *DST*, *GNAO1*, *ALCAM*, *NFASC*, *NEUROD4* and *ONECUT2* that were aberrantly expressed in FALS-derived cells. We found that these genes were associated with neuron differentiation, neuron development and cell adhesion and were enriched compared with the profiles of control MPCs (Aruga and Mikoshiba, 2003).

We reanalyzed a previous FUS CLIP-seq (crosslinking and immunoprecipitation, followed by high-throughput sequencing) dataset (Lagier-Tourenne et al., 2012) to determine whether this gene regulation is involved in direct regulation of the FUS protein. Among 10,159 FUS CLIP clusters, we defined 1,558 genes with tag number ≥20 and peak height ≥10; approximately 75% were located in intronic sequences (Figure 2D), which is consistent with previously reported FUS binding sites. We found that 23 genes were significantly overlapped using the exon array of 159 genes and potential FUS targets, as well as 1,558 genes based on the CLIP-seq dataset (Figures S3A, S3B, and Table S2). Most of these differently expressed genes were validated by qRT-PCR analysis (Figure 2E), indicating consistency between qRT-PCR and exon array analyses (Table S3).

**Figure 3:**
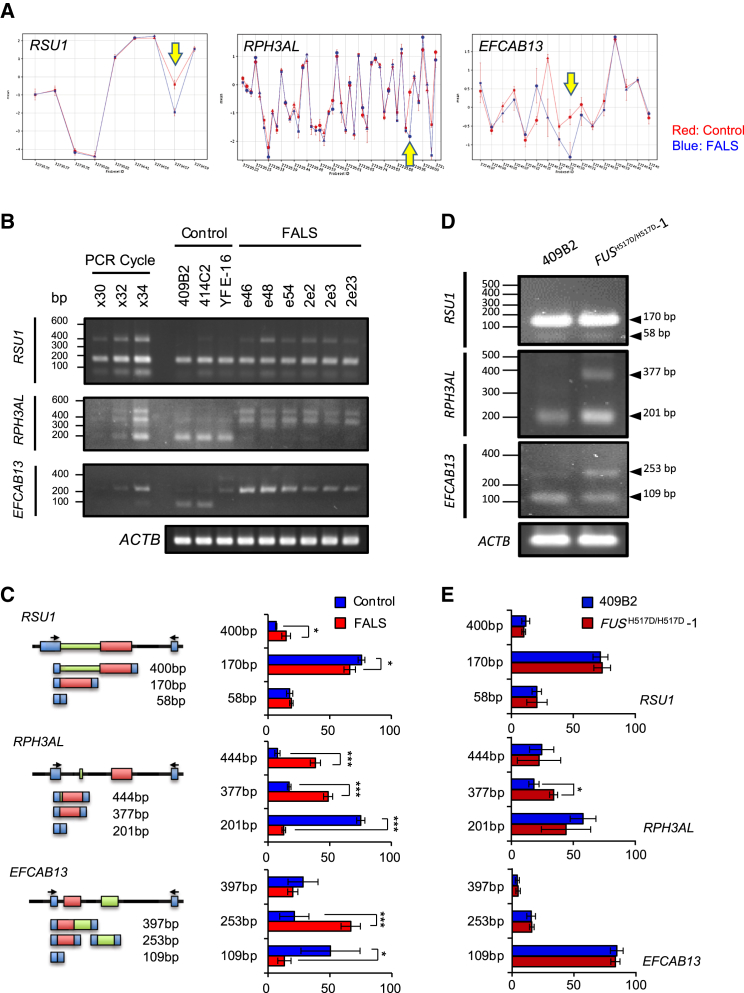
Alternative Splicing Analysis on MPCs (A) The plots of the expression levels in exon probes. Red and blue lines show control and FALS expression levels, respectively. The yellow arrows show the change points of splicing between controls and FALS in each gene. (B) RT-PCR of splicing variants in *RSU1*, *RPH3AL* and *EFCAB13* in iPSC-derived MPCs. The PCR cycle validated the PCR cycle numbers. (C) Schematic figure of alternative splicing in each gene (left) and the measurement of the expression level of each of the spliced bands in (B) (right) (n = 3–6 independent samples; mean ± SD; ^∗^p < 0.05; ^∗∗∗^p < 0.001; Student's t test). (D) RT-PCR of splicing variants in *RSU1*, *RPH3AL* and *EFCAB13* in 409B2 and *FUS*^H517D/H517D^-1 iPSC-derived MPCs. (E) Expression levels of each of the spliced bands in (D) (n = 3 independent experiments; mean ± SD; ^∗^p < 0.05; Student's t test).

We also analyzed the alternative splicing changes in control and FALS MPCs by exon array. We identified altered expression of exons between control and FALS MPCs including *RSU1* (Ras suppressor protein 1), *RPH3AL* (rabphilin 3A-like) and *EFCAB13* (EF-hand calcium binding domain 13), which had differences in expression levels on the core probes (Figure 3A). To validate these results by semi-quantitative RT-PCR assay (Figure 3B), we designed the primers flanking the splicing target exons in these genes and validated alternative splicing changes using total RNA obtained from tertiary MPC-specific cells by semi-quantitative RT-PCR analysis. As expected, these three genes showed dramatic splicing changes in FALS MPCs (Figure 3C). To confirm whether the splicing changes between control and FALS-derived cells depend on the mutation of the FUS protein, we analyzed the splicing patterns of these three genes in the MPCs derived from *FUS*^H517D/H517D^-iPSCs carrying the FUS H517D homozygous mutation. We confirmed that differential splicing changes also occur in the *RPH3AL* and *EFCAB13* genes between control and *FUS*^H517D/H517D^ MPCs (Figures 3D and S3C). Semi-quantitative analysis of splicing variants revealed that *FUS*^H517D/H517D^ MPCs express higher levels of the *RPH3AL* 377-bp band than 409B2 control MPCs, but express similar levels of *RSU1* and *EFCAB13* splicing bands (Figure 3E). These results suggest that aberrant gene expressions and/or splicing changes are associated partially with mutant FUS.

### Mutant FUS Is Localized in Stress Granules under Stress Conditions

**Figure 4:**
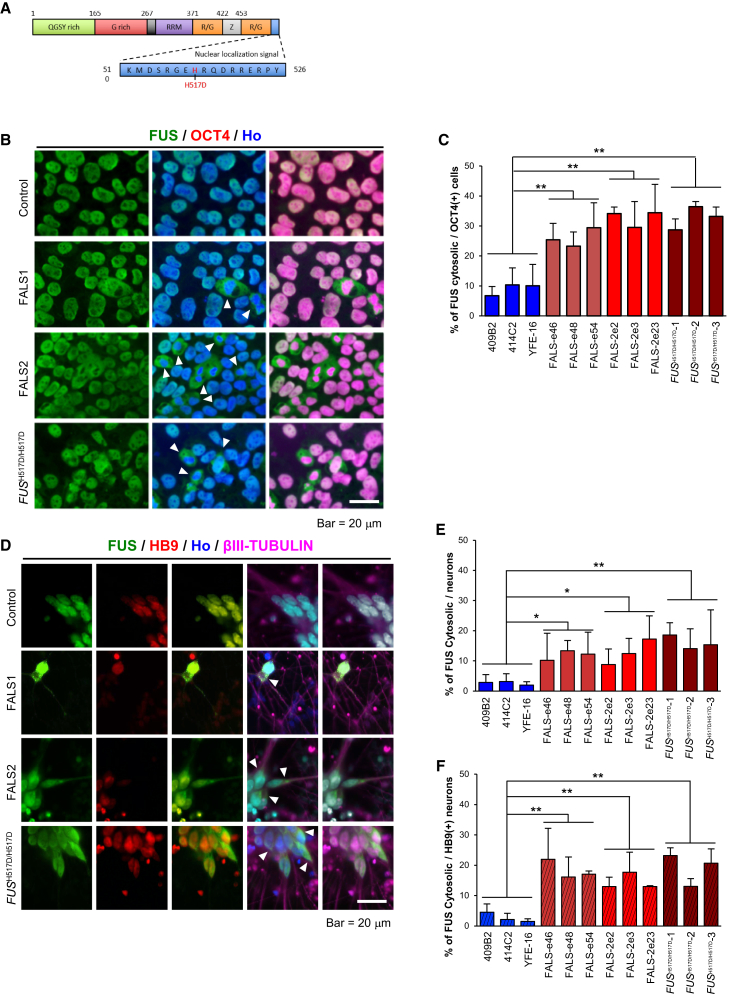
FUS Localization in iPSCs and iPSC-Derived Neurons (A) Schematic diagram of FUS. The H517D mutation is located in the nuclear localization signal; RRM, RNA recognition motif; R/G, R/G rich region; Z, zinc finger domain. (B) Representative images of immunocytochemistry for FUS in iPSCs. Arrowheads indicate cytosolic FUS. The scale bar represents 20 μm. (C) Quantitative data of the percentages of cytosolic FUS ratio per OCT4-positive cells in iPSCs (n = 3 independent experiments; mean ± SD; ^∗∗^p < 0.01; Dunnett's test). (D) Representative images of immunocytochemistry for FUS in iPSC-derived neurons. Arrowheads indicate cytosolic FUS. The scale bar represents 20 μm. (E) Quantitative data of the percentage of cytosolic FUS ratio per Hoechst-positive cell in iPSC-derived neurons (n = 3 independent experiments; mean ± SD; ^∗^p < 0.05, ^∗∗^p < 0.01; Dunnett's test). (F) Quantitative data of the percentage of cytosolic FUS ratio per HB9-positive cell in iPSC-derived neurons (n = 3 independent experiments; mean ± SD; ^∗∗^p < 0.01; Dunnett's test).

The H517D mutation in FUS lies in a nuclear localization signal (NLS) (Figure 4A). Previous reports revealed that the FUS protein localizes to the nucleus; however, the mutations in the NLS sequences of the FUS protein cause mis-localization in the cytoplasm, even under normal conditions. To address the possibility that the FUS protein from FALS iPSC-derived cells harboring the H517D mutation also mis-localizes to the cytoplasm, we performed immunocytochemical analysis. The data confirmed that there is cytoplasmic mis-localization of the FUS protein in FALS and *FUS*^H517D/H517D^ iPSCs (Figure 4B) and we determined the ratio of cytosolic FUS using IN Cell Analyzer (Figure 4C). The FUS proteins in FALS and *FUS*^H517D/H517D^ iPSC-derived neuronal lineage cells (Figures 4D and 4E) and HB9-positive motor neurons (Figures 4D and 4F) were also mis-localized into cytosol. In addition, we performed biochemical analysis to measure the expression levels of *FUS* mRNA. Control, FALS and *FUS*^H517D/H517D^ MPCs and neurons expressed similar levels of the *FUS* gene (Figure S4).

**Figure 5:**
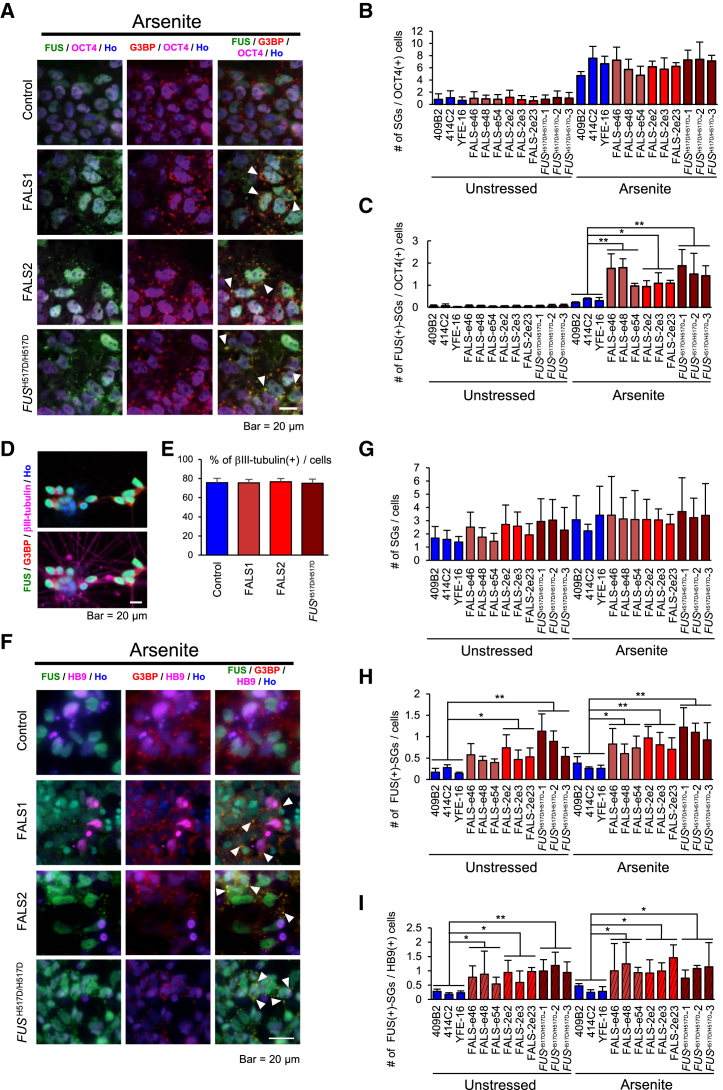
FUS Protein Localization into Stress Granules (A) Representative images of immunocytochemistry for SG in iPSCs under 0.5 mM sodium arsenite stress conditions. FUS in FALS and *FUS*^H517D/H517D^ iPSCs co-localized with the SG marker G3BP (arrowhead), whereas FUS in control iPSCs remained in the nucleus. The scale bar represents 20 μm. In (A)–(I) images or graphs, Arsenite means addition of 0.5 mM sodium arsenite, 1 hr treatment. (B) Quantitative data of the number of SGs per OCT4-positive cell in iPSCs (n = 3 independent experiments; mean ± SD; Dunnett's test). (C) Quantitative data of the number of FUS-positive SGs per OCT4-positive cell in iPSCs (n = 3 independent experiments; mean ± SD; ^∗^p < 0.05, ^∗∗^p < 0.01; Dunnett's test). (D) Representative images of immunocytochemistry for βIII-TUBULIN-positive neurons. The scale bar represents 20 μm. (E) Quantitative data of the percentage of βIII-TUBULIN-positive neurons (n = 3 independent experiments; mean ± SD; Dunnett's test). (F) Representative images of immunocytochemistry for SG in iPSC-derived neurons under 0.5 mM sodium arsenite stress conditions. FUS co-localized with the SG marker G3BP (arrowhead). The scale bar represents 20 μm. (G) Quantitative data of the number of SGs per Hoechst-positive cell in iPSC-derived neurons (n = 3 independent experiments; mean ± SD; Dunnett's test). (H) Quantitative data of the number of FUS-positive SGs per Hoechst-positive cell in iPSC-derived neurons (n = 3 independent experiments; mean ± SD; ^∗^p < 0.05, ^∗∗^p < 0.01; Dunnett's test). (I) Quantitative data of the number of FUS-positive SGs per HB9-positive cell in iPSC-derived neurons (n = 3 independent experiments; mean ± SD; ^∗^p < 0.05, ^∗∗^p < 0.01; Dunnett's test).

It has been reported that mutant FUS localizes into cytoplasmic stress granules (SGs) upon various stimuli (Aulas et al., 2012, Bentmann et al., 2012, Vance et al., 2013). SGs are cytosolic structures that transiently form upon exposure of cells to environmental stress, such as heat, oxidative stress, or hypoxia functions in the cellular defense against stress, as well as translational repression of a subset of mRNAs (Nishimoto et al., 2010). We induced oxidative stress in iPSCs and iPSC-derived neurons by treatment with 0.5 mM sodium arsenite for 60 min to observe the formation of SGs using anti-G3BP (Ras GTPase-activating protein-binding protein) as a marker of SGs for immunocytochemistry. First we found that FUS proteins from FALS and *FUS*^H517D/H517D^ iPSCs and iPSC-derived neurons leak and form aggregates in the cytosol, and that their aggregates co-localize with SGs (Figure S5A). In contrast, wild-type FUS remained in the nucleus and did not form G3BP-positive cytoplasmic granules (Figures 5A and 5F). Next, we determined the number of all SGs and FUS-positive SGs per OCT4-positive iPSCs or HB9-positive motor neurons using IN Cell Analyzer. The number of all SGs in iPSCs was unchanged in all lines (Figure 5B); however, the levels of FUS-positive SGs are significantly higher in FALS- and *FUS*^H517D/H517D^-iPSCs than in controls (Figure 5C). We next examined similar assays in neuronal lineages. Importantly, we confirmed that the number of neuronal cell populations and all SGs in the differentiating cells in our culture was not significantly changed among all the lines (Figures 5D, 5E and 5G). FALS- and *FUS*^H517D/H517D^-iPSC-derived neuronal lineage cells and HB9-positive motor neurons showed higher levels of FUS-positive SGs than did controls with significant changes (Figures 5H and 5I). We obtained similar results under the condition of 44°C heat shock (Figures S5B–S5E). In addition, FALS iPSC lines expressed both H517D-mutant and normal *FUS*; therefore, we used a transient expression assay to confirm whether the H517D mutant FUS protein is localized into cytoplasmic SGs under stress conditions. The 293T cell lines were transfected with plasmids encoding either wild-type FUS or H517D mutated FUS (Figure S5F). Expectedly, only the H517D FUS mutant was co-localized with G3BP-positive SGs under the treatment with arsenite but not wild-type FUS, reflecting our observation in FUS iPSC lines (Figure S5G).

### Stress Vulnerability in FALS Motor Neurons

**Figure 6:**
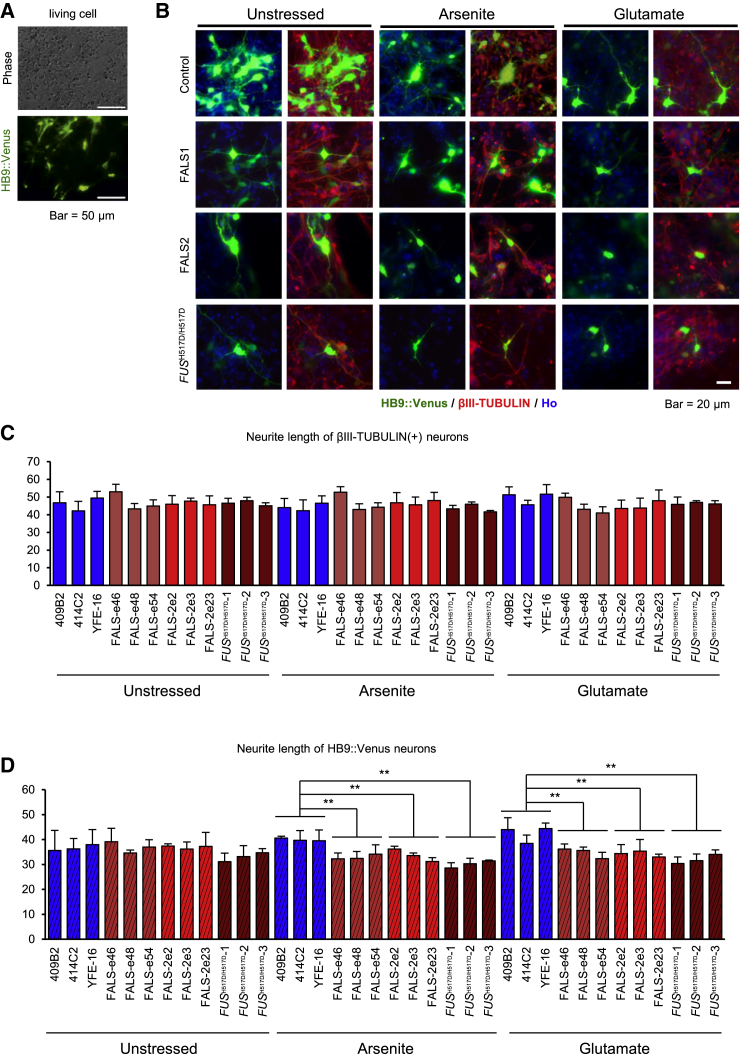
Shorter Neurites in FALS iPSC-Derived Motor Neurons (A) Representative images of HB9::Venus-positive living motor neurons. The scale bars represent 50 μm. (B) Representative images of immunocytochemistry for HB9::Venus-positive motor neurons with anti-GFP antibody. The scale bar represents 20 μm. In (B)–(D) images or graphs, Arsenite means 1.0 mM sodium arsenite, 1 hr treatment; Glutamate means 1.0 mM glutamate, 24 hr treatment. (C) Quantitative data of the neurite length of βIII-TUBULIN-positive neurons (n = 3 independent experiments; mean ± SD; Dunnett's test). (D) Quantitative data of the neurite length of GFP-positive motor neurons (n = 3 independent experiments; mean ± SD; ^∗∗^p < 0.01; Dunnett's test).

**Figure 7:**
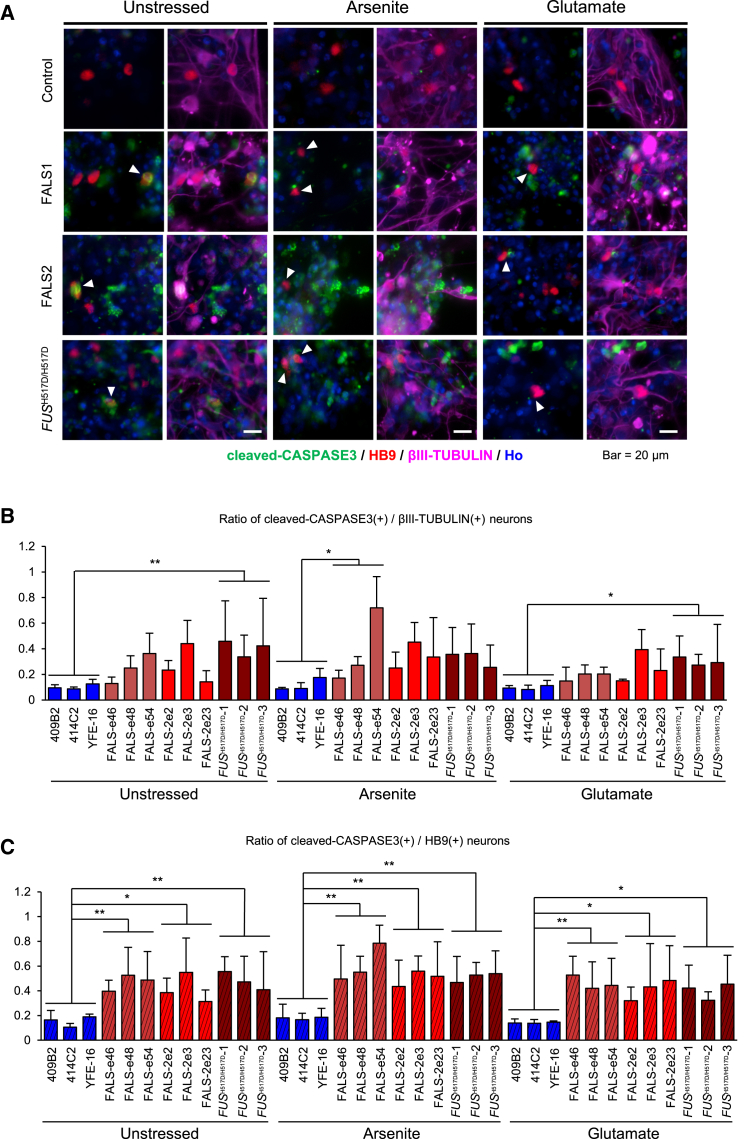
Enhanced Apoptosis in FALS iPSC-Derived Motor Neurons (A) Representative images of immunocytochemistry for apoptotic HB9-positive motor neurons using markers for apoptosis (cleaved-CASPASE3), immature neurons (βIII-TUBULIN), and motor neurons (HB9). Arrowheads indicate HB9 positive cells. The scale bars represent 20 μm. In (A)–(C) images or graphs, Arsenite means 0.5 mM sodium arsenite, 1 hr treatment; Glutamate means 3.0 mM glutamate, 24 hr treatment. (B) Quantitative data of the ratio of cleaved-CASPASE3-positive cells in βIII-TUBULIN-positive neurons (n = 3 independent experiments; mean ± SD; ^∗^p < 0.05, ^∗∗^p < 0.01; Dunnett's test). (C) Quantitative data of the ratio of cleaved-CASPASE3-positive cells in HB9-positive motor neurons (n = 3 independent experiments; mean ± SD; ^∗^p < 0.05, ^∗∗^p < 0.01; Dunnett's test).

While ALS is an adult-onset neurodegenerative disease that specifically targets motor neurons, GO analysis and overlapping genes with CLIP-seq predicted that FALS motor neurons are involved in neural development (Table S2). To address the possibility that FALS iPSC-derived neurons display any defects of neuronal maturation, we measured neurite length on motor neurons. Neural cell populations derived from iPSCs included various neuronal subtypes. Therefore, to label only motor neurons, HB9-venus reporter lentivirus (HB9:Venus) (Shimojo et al., 2015) was infected into the iPSC-derived cells. Motor neurons were visualized by Venus fluorescent protein (Figure 6A); we identified motor neurons labeled by the Venus fluorescent protein with the anti-GFP antibody to determine the length of neurites by IN Cell Analyzer (Figure 6B). As a result, there were no differences between controls, FALS, and *FUS*^H517D/H517D^-derived βIII-TUBULIN-positive neurons under all conditions: unstressed, sodium arsenite treatment and glutamate treatment (Figure 6C). In HB9:Venus-positive motor neurons, however, FALS and *FUS*^H517D/H517D^ neurons showed significantly shorter neurites than controls by treatment with both sodium arsenite and glutamate (Figure 6D). These results suggest that FALS-derived neurons might show not only neuronal maturation (in this case, neurite maintenance) but also cellular vulnerability especially in motor neurons. Therefore, we next pursued the cell viability of FALS-derived neurons and HB9-positive motor neurons under normal and stress conditions by immunostaining with cleaved-CASPASE3, which is a marker for apoptosis (Figure 7A). In all neurons labeled with βIII-TUBULIN, we found significant differences between control and *FUS*^H517D/H517D^ lines; this may be due to homozygous mutation of FUS protein (Figure 7B). However, we were unable to confirm this as a single population of the FALS line also has a significant change. On the other hand, in HB9-positive motor neurons, the changes in the apoptotic cell population were much greater between control and all FALS and *FUS*^H517D/H517D^ lines (Figure 7C). These results suggest that iPSC-derived HB9-positive motor neurons with FUS H517D mutation are vulnerable to stress and even with normal conditions rather than other types of neurons, this phenomenon reflects the ALS-like malady phenotype. Therefore we conclude that our in vitro FALS model is a useful tool to pursue the mechanism underlying the disease phenotype and treatment.

## Discussion

In the present study, we established ALS-specific human iPSCs from two patients with a point mutation in the *FUS* gene and isogenic iPSCs with FUS H517D homozygous mutations derived from healthy 409B2 iPSCs using the TALEN genome editing system. In addition, we differentiated into patient derived neurons to observe the disease history of FALS during neuronal differentiation in vitro. FALS-derived motor neuron lineage cells and HB9-positive mature motor neurons showed several ALS-related phenotypes such as neuronal cell death, pathological cellular structure and altered gene regulation, including steady-state transcript levels and alternative splicing. Our in vitro model may thus enable an investigation into the correlations between the molecular pathophysiology of ALS and various cell biological phenomena.

RNA-mediated mechanisms in ALS originated from the first discovery of ubiquitinated TDP43 protein, which is an RNA-binding protein (Neumann et al., 2006). Subsequent studies found the mutation in this gene among FALS (Ricketts et al., 2014, Rutherford et al., 2008, Sreedharan et al., 2008). TDP43-containing protein inclusions in cells ultimately came to be recognized as a pathological hallmark in ALS and frontotemporal lobar degeneration (FTLD). The other RNA-binding protein, the FUS/TLS protein coding gene, also contains potential causative mutations in FALS and in FTLD (Kwiatkowski et al., 2009, Vance et al., 2009). In the present study, the mutant FUS protein was co-localized with the stress marker protein G3BP in our cultured iPSCs, HB9-positive motor neurons derived from them, and fibroblast cell line overexpressing FUS protein responding to stress conditions, arsenite and heat shock (Figures 5F and S5). In general, the stress granule is a mechanism for avoiding stress and has been implicated in the cellular stress defense. These granules are in equilibrium between assembly and disassembly to manage correct gene regulation with cellular conditions. One such pathological hypothesis considers that once this equilibrium is disrupted, these stress granules form irreversible protein inclusions, named “pathological aggregates.” In our in vitro model, we observed that FUS mutant proteins localize to SGs in iPSC-derived HB9-positive motor neurons, even under unstressed conditions; the co-localization of FUS and G3BP has not been observed in wild-type cells expressing normal FUS. This suggests the possibility that over-migration of FUS into SGs potentially causes aberrant gene expression and/or splicing, and that these granules may eventually form pathological aggregates, leading to neuronal cell death.

FUS binds to DNA as well as to RNA and regulates the expression of many transcripts in multiple steps of the gene regulation process (Dormann and Haass, 2013). In this study, we used FALS MPCs efficiently oriented into neuronal lineages including HB9-positive motor neurons to observe altered gene regulation in FALS-derived cells and to discover an early disease-related diagnostic marker. FUS has been shown to associate with RNA polymerase II and TFIID, thereby participating in the general transcriptional regulation process (Bertolotti et al., 1996). In addition, FUS binds to TBP and TFIID to repress transcription by RNAPIII, suggesting that FUS controls the cross-regulation between RNA polymerases (Tan and Manley, 2010). Recent studies, however, have shown that the recruitment of FUS proteins to promoter regions with lncRNA represses transcription (Wang et al., 2008). Furthermore, FUS binding to the antisense RNA transcribed by RNAPIII from promoter regions downregulates transcription (Ishigaki et al., 2012). These past studies suggest that FUS regulates transcriptional repression by various mechanisms in specific target genes. This finding could reflect our observations from our microarray assay that 78% (124 out of 159 genes) of the significantly changed genes in transcript levels are upregulated in FALS MPCs. Furthermore, with more specific analysis that focused on FUS direct targets that we defined by using the CLIP-seq dataset, we found that 95.6% of genes (of 23 genes) are upregulated in FALS.

In one proteomics study, FUS/TLS proteins were also identified as general splicing factors, which may be an early stage of the splicing process (Hartmuth et al., 2002). Our exon array analysis also revealed aberrant gene splicing events in *RSU1*, *RPH3AL*, and *EFCAB13* genes in the FALS patients. Of these, aberrant RNA processing of *RPH3AL* was also confirmed by our isogenic *FUS*^H517D/H517D^ lines, suggesting the direct effects of FUS H517D mutation. However, in the other two alternative exons, we did not detect significant differences between control and isogenic *FUS*^H517D/H517D^ lines, suggesting that FUS H517D mutation is not likely to have direct effects on their splicing regulation. RPH3AL is an associated protein of RAB3A (Haynes et al., 2001) and RAB27A (Fukuda, 2003) and regulates exocytosis in dense-core granules from endocrine cells (Haynes et al., 2001). In addition, mutant RPH3AL is mis-localized throughout the cytosol, whereas WT-RPH3AL is localized in the distal portion of the neurites (Fukuda et al., 2004). To date, there has been no reports of functional analyses of products from each alternative splicing event in these three genes. This may be involved in the pathological features of FUS-mediated pathologies and may also be useful as early diagnostic markers for ALS. Our iPSC model for FALS may thus represent a useful tool for observing gene expression levels in the motor neuron lineage, as gene expression analysis in motor progenitor cells oriented into HB9-positive motor neurons cannot be achieved using conventional disease models.

We also addressed whether pathological features observed in other ALS models are observed in our in vitro FALS model. Motor neurons in patients with sporadic ALS express abundant unedited GLUA2 forms in the AMPA receptor subunit (Kawahara et al., 2004, Kwak et al., 2010), but the unedited GLUA2 form in FALS MPCs and neurons derived from iPSCs was not detected (Figure S6). ER stress is increasingly recognized as an important pathway leading to cell death in animal and cellular disease models based on mutant SOD1 (Atkin et al., 2006, Saxena et al., 2009), and mutant FUS proteins would also be predicted to induce ER stress and to interact with protein disulfide isomerase, similar to mutant SOD1 (Farg et al., 2012). In this study, however, the expression of ER stress-related genes (*BIP*, *CHOP*, spliced *XBP1*, *CASP4*, and *ASK1*) almost did not change in FALS and *FUS*^H517D/H517D^ MPCs and neurons (Figure S7).

Our study showed that neurite length and the ratio of apoptotic cells in FALS- and *FUS*^H517D/H517D^ iPSC-derived neurons were the same as in control motor neurons. With several stresses especially in glutamate treatment, however, FALS- and *FUS*^H517D/H517D^ HB9-positive motor neurons exhibited shorter neurites and an increased cleaved-CASPASE3-positive cell ratio compared with control HB9-positive motor neurons. These results suggest that FALS iPSC-derived motor neurons are vulnerable to oxidative and excitatory stresses and there is a possibility that these phenomena are relevant to ALS-like phenotypes. We thus determined that these ALS patient-specific iPSC-derived motor neurons recapitulate disease phenotypes.

Recently, other groups also established in vitro FUS iPSCs models showing the inclusion of the stress granule of FUS mutant proteins (Lenzi et al., 2015, Liu et al., 2015). In addition to these findings, we observed additional ALS-like phenotypes and motor neuron vulnerability under some stress conditions and discovered potential biologically relevant aberrant gene expressions. Importantly, these events are also confirmed by the use of genome editing technology to generate isogenic mutant FUS H517D, suggesting that the present in vitro FALS model is able to recapitulate the ALS-like phenotypes. Based on these results, our mutant FUS-associated iPSC-derived HB9-positive motor neurons can be added to the list of model systems that may provide general tools for use in the analysis of the pathogenic process and drug screening studies in human motor neuron disorders.

## Experimental Procedures

### Isolation of Human Skin Fibroblasts and Generation of iPSCs

The 409B2 and 414C2 iPSCs were kindly provided by Dr. Shinya Yamanaka (Okita et al., 2011). A skin-punch biopsy from a healthy 24-year-old Japanese man obtained after written informed consent (Keio University School of Medicine) was used to generate the control YFE-16 (Shimojo et al., 2015) iPSCs. FALS-1 and FALS-2 iPSCs were generated from a 39-year-old Japanese man and 43-year-old Japanese man, respectively. All the human iPSC clones were established by episomal vector transduction of transcription factors (SOX2, OCT4, KLF4, L-MYC, LIN28 and short hairpin RNA of p53) into human dermal fibroblasts (Okita et al., 2011). The cells were evaluated as described previously (Egawa et al., 2012, Müller et al., 2011, Okita et al., 2011). All the experimental procedures for skin biopsy and iPSC production were approved by the Keio University School of Medicine Ethics committee (approval number, 20080016) and Tohoku University School of Medicine Ethics committee (approval number, 2010-306).

### Motor Neuron Differentiation

Motor neuron differentiation of iPSCs was performed as previously described with slight modifications (Imaizumi et al., 2015). Briefly, iPSCs were exposed to a medium including 3 μM dorsomorphin (Sigma), 3 μM SB431542 (Tocris Bioscience), and 3 μM CHIR99021 (Stemgent) over 5 consecutive days. Next, iPSCs were detached from feeder layers and then enzymatically dissociated into single cells. The dissociated cells were cultured in suspension in media hormone mix (MHM) (Okada et al., 2008) including 2% B27 supplement (Life Technologies), 2 ng/ml basic fibroblast growth factor (PeproTech), 3 μM CHIR99021 (Stemgent), 2 μM SB431542 (Tocris Bioscience), 10 μM Y27632 (Wako), 1 μM retinoic acid (Sigma), 1 μM purmorphamine (Calbiochem), and 10 ng/ml recombinant human leukemia inhibitory factor (Millipore) for 5–24 days to allow the formation of MPCs. MPCs were passaged repeatedly by dissociation into single cells followed by culture in the same manner. Typically, MPCs at three passages were used for analysis. The starting cell density was 1 × 10^4^ cells/ml in primary MPC culture and 1 × 10^5^ cells/ml in secondary and tertiary MPC cultures. For terminal differentiation, dissociated MPCs were allowed to adhere to poly-L-ornithine- and laminin-coated dishes with 5 × 10^4^ cells/well in 96-well plate in MHM/B27 including 1× N2 supplement (Gibco), 1× GlutaMAX (Gibco), 10 ng/ml recombinant human brain-derived neurotrophic factor (R&D Systems), 10 ng/ml recombinant human glial cell line-derived neurotrophic factor (R&D Systems), 50 ng/ml recombinant hSHH (R&D Systems), 10 ng/ml insulin growth factor 1 (R&D Systems), 200 ng/ml ascorbic acid, 50 nM retinoic acid (Sigma), and 1 μM dibutyryl cyclic AMP (Sigma), and cultured for 20 days. Glutamate was added in the last 24 hr and sodium arsenite was added in the last 1 hr.

### Immunocytochemistry

Immunocytochemistry was performed as described previously (Imaizumi et al., 2015). Detailed conditions are given in the Supplemental Information (Table S4). Fluorescence images were acquired on AxioVision (Zeiss), BZ-9000 (Keyence), or IN Cell Analyzer (GE Healthcare).

### High-Content Analysis

For the MPC population assay, neuronal subtype assay, neurite length analysis and cleaved-CASPASE3 analysis, stained plates were imaged on the high-content cellular analysis system IN Cell Analyzer 6000 (GE Healthcare) and a set of 5 × 5 fields were collected from each well using the 20× objective, resulting in over 10,000 cells being scored per well. For FUS mis-localization analysis and stress granule analysis, stained plates were imaged on IN Cell Analyzer 6000 and a set of 6 × 6 fields were collected from each well using the 60× objective, resulting in over 9,000 cells being scored per well. Analysis (IN Cell Developer Toolbox v1.9; GE Healthcare) began by identifying intact nuclei stained by Hoechst, which were defined as traced nuclei that were larger than 50 μm^2^ in surface area and with intensity levels that were typical and lower than the threshold brightness of pyknotic cells. Each traced nucleus region was then expanded by 50% and cross-referenced with MPC markers (OLIG2, SOX2 and ISLET1), motor neuron markers (ISLET1, SMI32 and HB9), glutamatergic neuron marker (VGLUT1), glutamate-responsive neuron marker (GLUR1), neuron markers (MAP2 and βIII-TUBULIN), and pluripotent marker (OCT4) to identify them; from these images, the percentages of these were calculated. By setting areas on each cell type or neural subtype, the ratio of FUS mis-localization into cytosol, the number of stress granules, or the number of FUS-positive stress granules in OCT4-positve iPSCs, βIII-TUBULIN-positive neurons or HB9-positive motor neurons were analyzed. Using the above-described traced images of each cell, neurite length and the cleaved-CASPASE3-positive cell ratio in βIII-TUBULIN-positive neurons or HB9-positive motor neurons were analyzed.

### Quantitative RT-PCR

RNA was isolated using an RNeasy kit (Qiagen) and reverse transcription using iScript cDNA Synthesis Kit (Bio-Rad). Quantitative RT-PCR was performed using SYBR Premix Ex Taq II (TaKaRa) on the ViiA 7 Real-Time PCR System (Life Technologies) (Table S5).

### Sequence Analysis

Genomic DNA was isolated using a DNeasy kit (Qiagen) and amplified using intronic primers and direct nucleotide sequencing (Table S5). Both sense and antisense strands of all amplicons were sequenced using the Big Dye 3.1 dideoxy terminator methods (Applied Biosystems) and ABI Prism 3130xL Genetic Analyzer (Applied Biosystems).

### Exon Array for MPCs

Exon array analysis was performed using an Affymetrix GeneChip Human Exon 1.0 ST Array. Data were analyzed using the GeneSpring GX7.3.1 software (Agilent), UCSC Genome browser (http://genome.ucsc.edu/index.html), and DAVID Bioinformatics Resources (http://david.abcc.ncifcrf.gov/). Exon array data have been registered in the Gene Expression Omnibus under accession number GEO: GSE76698.

## Author Contributions

N.I., K.F., M.Y., and H.O. conceived and designed the experiments and wrote the manuscript. N.I. and K.F. performed most of the experiments and analyzed the data. N.I., C.I.-F., T.S., T.A., Y.O., W.A., T.M., M.I., Y.I., T.S., and T.Y. contributed to generate the patient-derived hiPSCs, isogenic hiPSCs, and analyzed the culture assay results. N.I., Y.N., H.T., and M.Y. analyzed and validated the microarray data and helped with in vitro analysis. N.S., H.W., and M.A. contributed to clinical and genetic analyses of the patient-coordinated study. All the authors read and approved the final version of the manuscript.

## Supplemental Information

Document S1. Supplemental Experimental Procedures, Figures S1–S7, and Tables S1–S5

Document S2. Article plus Supplemental Information
