# Supplementary material for: Establishment of In Vitro FUS-Associated Familial Amyotrophic Lateral Sclerosis Model Using Human Induced Pluripotent Stem Cells
Source: Stem Cell Reports. 2016 Mar 17;6(4):496–510. doi: 10.1016/j.stemcr.2016.02.011 (PMC4834049; doi:10.1016/j.stemcr.2016.02.011)
Supplement: Document S1. Supplemental Experimental Procedures, Figures S1–S7, and Tables S1–S5 [file mmc1.pdf]

Stem Cell Reports, Volume *6*

Supplemental Information

Establishment of In Vitro FUS-Associated Familial Amyotrophic Lateral Sclerosis Model Using Human Induced Pluripotent Stem Cells

Naoki Ichiyanagi, Koki Fujimori, Masato Yano, Chikako Ishihara-Fujisaki, Takefumi Sone, Tetsuya Akiyama, Yohei Okada, Wado Akamatsu, Takuya Matsumoto, Mitsuru Ishikawa, Yoshinori Nishimoto, Yasuharu Ishihara, Tetsushi Sakuma, Takashi Yamamoto, Hitomi Tsuiji, Naoki Suzuki, Hitoshi Warita, Masashi Aoki, and Hideyuki Okano

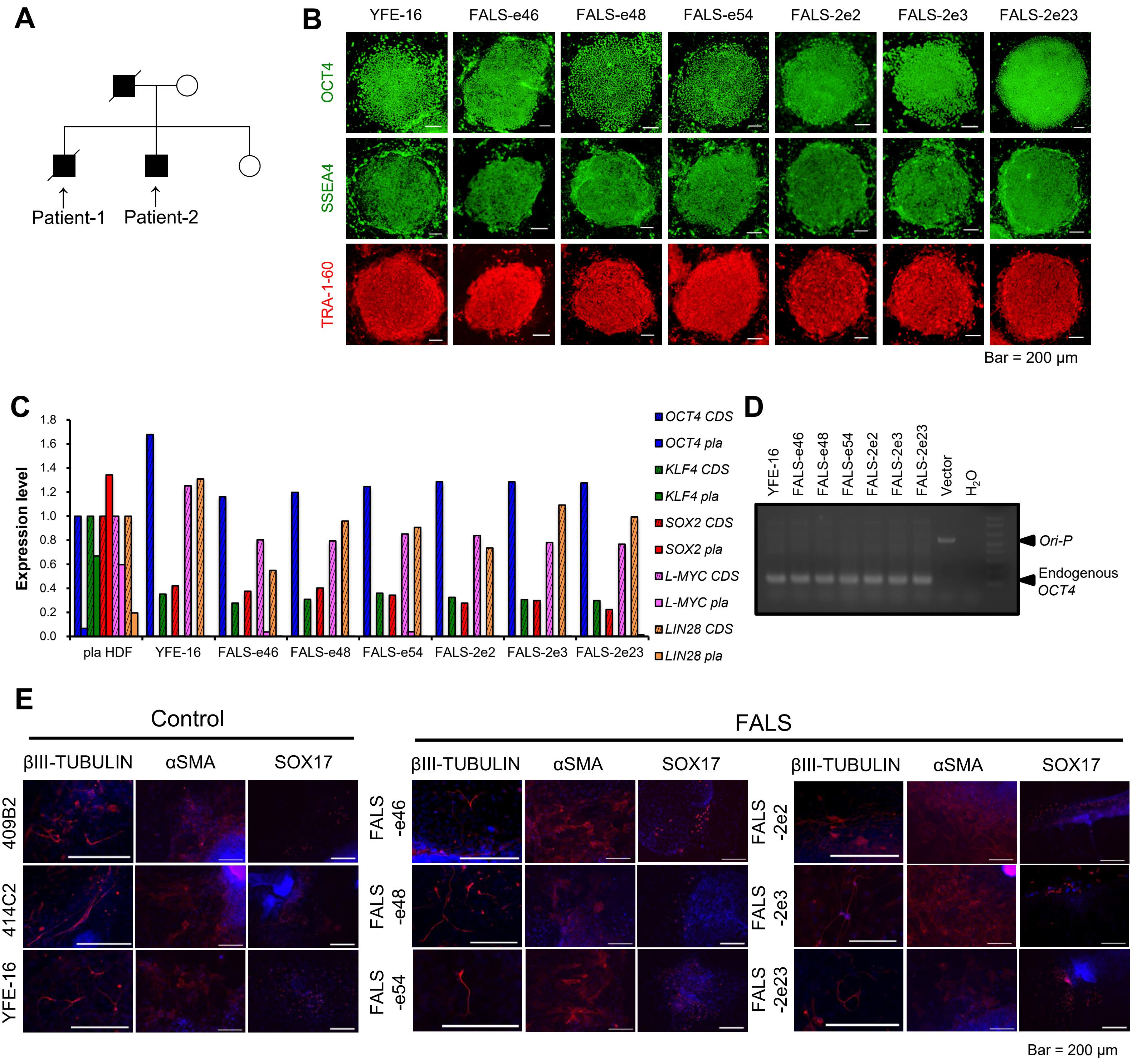

Figure S1. Pluripotent markers, Transgene expression, Genome integration, and *in vitro* differentiation into three-germ layers (Related to Figure 1)

A. Pedigree of the patients carrying *FUS*^{H517D} mutation (extracted from Akiyama et al. (2016)). iPSCs were generated from Patient-1 and Patient-2. The detailed pedigree tree of this family is described in Akiyama et al. (2016).

B. Representative image of immunochemical analysis of the pluripotent markers, OCT4, SSEA4, and TRA-1-60. The scale bars represent 200 μm.

C. Quantitative RT-PCR analysis of the expression of transgenes of episomal vectors in established YFE-16 and FALS iPSC clones. The data are presented as the mRNA copy numbers for each transgene divided by those for *ACTB*. As a control, fibroblasts at 6 days after transfection of 5 episomal vectors were analyzed (pla HDF). CDS, for detection of coding sequence; pla, for detection of plasmid vector-derived expression.

D. Integrated episomal vectors were detected by PCR for *Ori-P* cassette in established YFE-16 and FALS iPSC clones. Endogenous *OCT4* was detected for the expression control, and Vector and H2O were positive and negative controls, respectively.

E. Representative image of immunocytochemistry for the in vitro three-germ layer assay. βIII-TUBULIN, αSMA, and SOX17 are the markers of ectoderm, mesoderm, and endoderm, respectively. Scale bars, 200 μm.

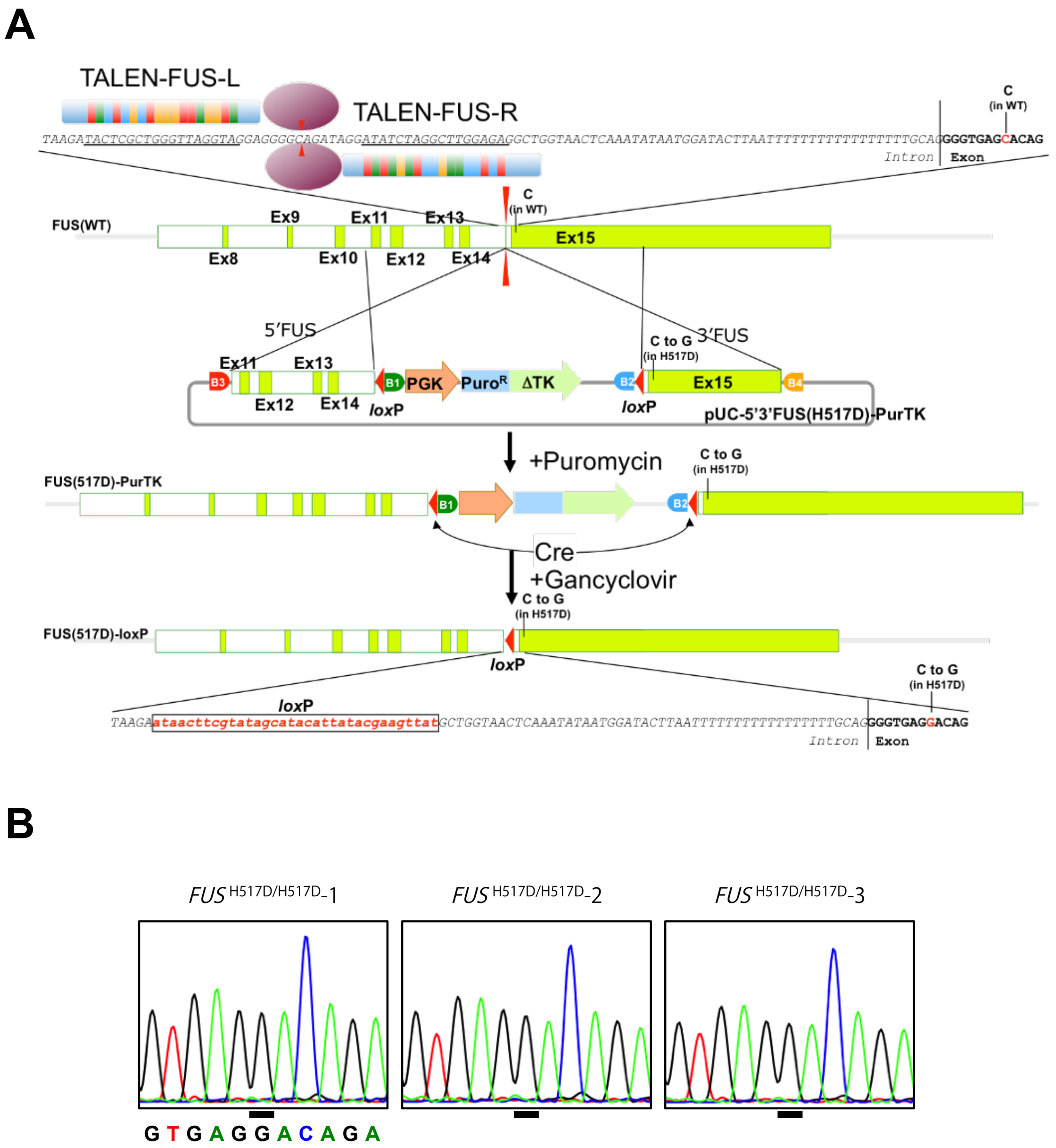

Figure S2. TALEN-mediated genome editing in iPSC (Related to Figure 1)

A. Schematic presentation of the protocols for TALEN-mediated genome editing in iPSC.

B. The *FUS* H517D (C-to-G) homozygous mutations identified in TALEN-mediated iPSCs, *FUS*$^{H517D/H517D}$-1, -2, and -3.

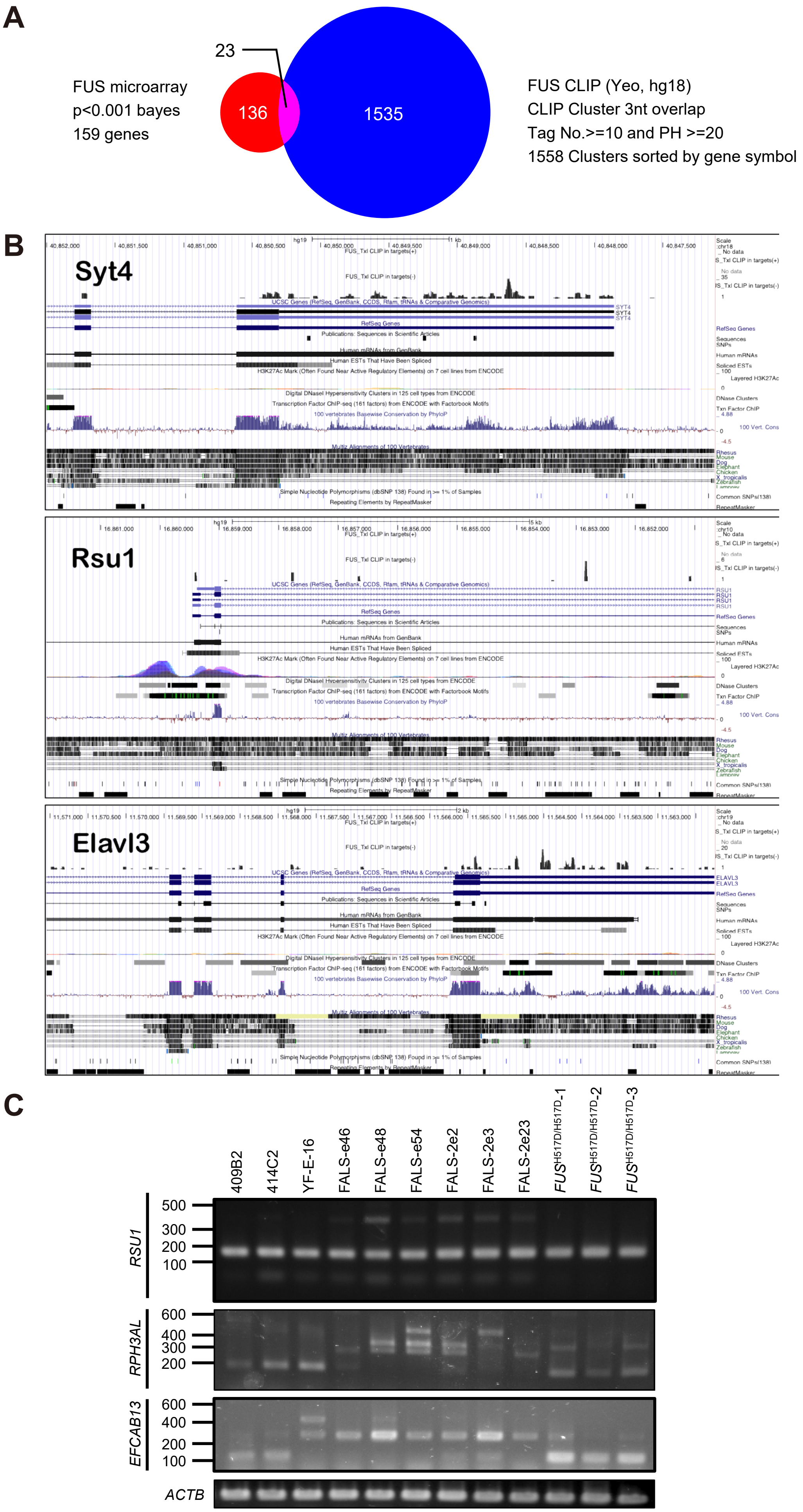

Figure S3. Comparison with exon array and CLIP-seq, and alternative splicing analysis on MPCs (Related to Figure 2 and Figure 3)

A. Venn diagram showing the number of exon array clusters (red) and FUS CLIP-seq clusters (blue) that overlap (purple) each other. Exon array clusters were then restricted within p<0.001 in Bayesian statistics, and CLIP-seq clusters were then restricted within a 3 nt overlap, tag numbers of more than 10, and peak height (PH) of more than 20.

B. The graphic view from the genome browser showing general gene information, scale, chromosomal coordinates, and the genes *SYT4*, *RSU1*, and *ELAVL3*.

C. Representative image of RT-PCR of splicing variants in *RSU1*, *RPH3AL*, and *EFCAB13* in iPSC-derived MPCs.

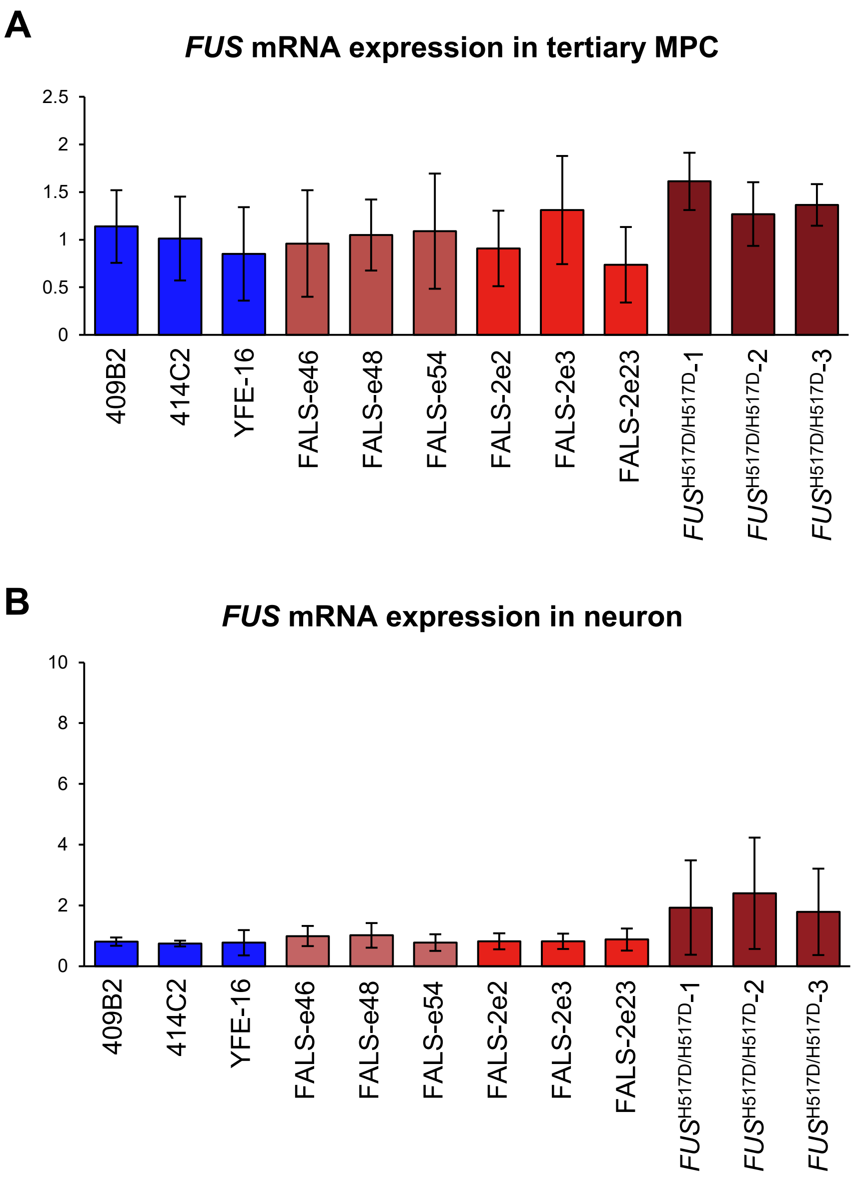

Figure S4. *FUS* gene expression (Related to Figure 4)

Quantitative RT-PCR analysis of the expression of the *FUS* gene in MPCs (A) and neurons (B). The data are presented as the mRNA copy numbers divided by *ACTB*. (n = 3 independent experiments; mean ± SD; Dunnett's test)

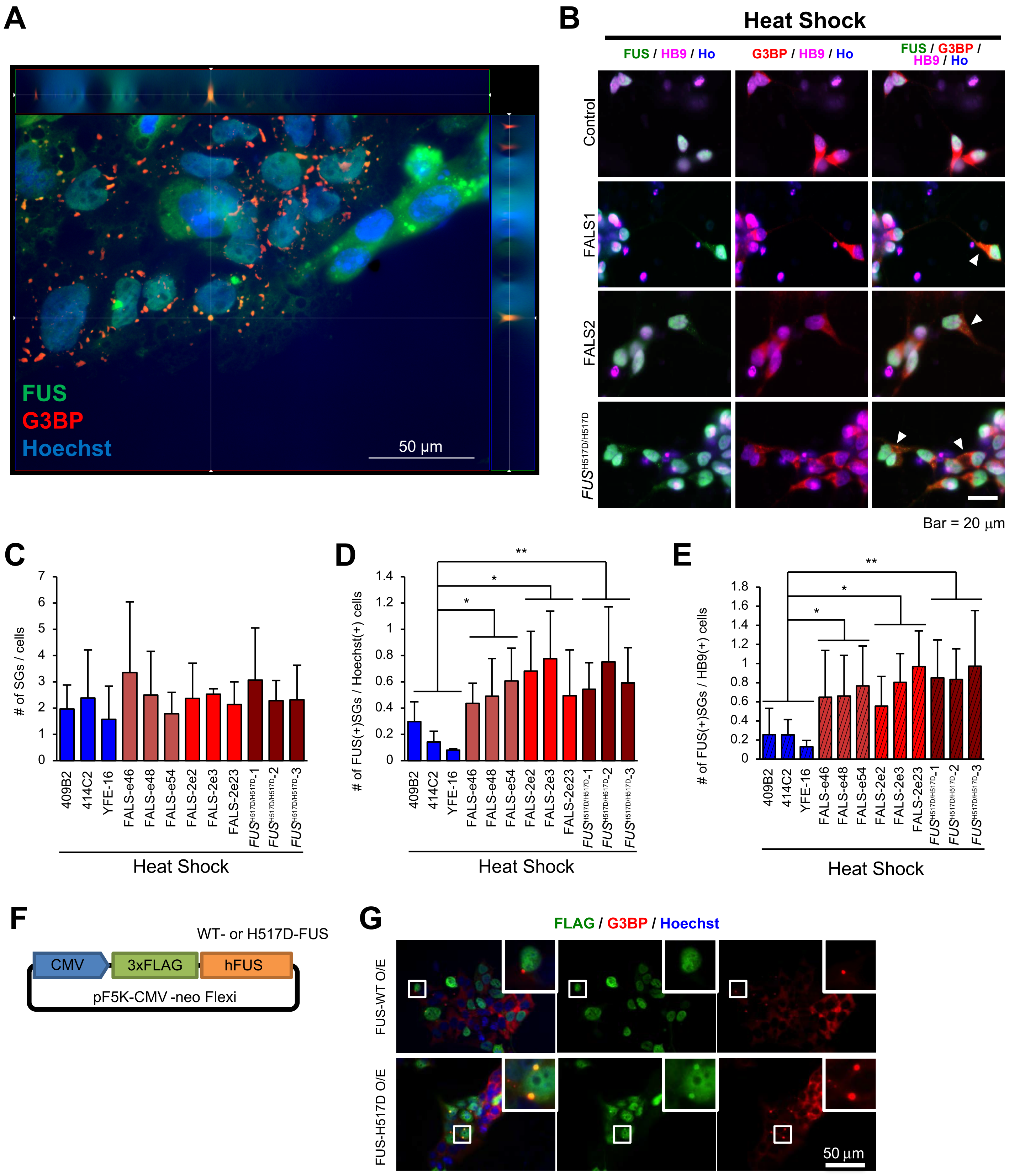

Figure S5. FUS protein localizes into stress granules under heat shock stress condition in iPSC and iPSC-derived neuron, and under arsenite treatment in FUS overexpression system (Related to Figure 5)

A. Representative orthogonal image of immunocytochemistry for FUS and SGs. FUS-positive aggregates were wrapped by G3BP-positive SGs. The scale bar represents 50 µm.

B. Representative images of immunocytochemistry for SG in iPSC-derived neurons under 44 °C heat shock condition. FUS co-localized with the SG marker G3BP (arrowhead). The scale bars represent 20 µm.

C. Quantitative data of the number of SGs per Hoechst-positive cells in iPSC-derived neurons under 44 °C condition (n = 3 independent experiments; mean ± SD; Dunnett's test).

D. Quantitative data of the number of FUS-positive SGs per Hoechst-positive cells in iPSC-derived neurons under 44 °C condition (n = 3 independent experiments; mean ± SD; Dunnett's test).

E. Quantitative data of the number of FUS-positive SG in HB9-positive motor neurons under 44 °C condition (n = 3 independent experiments; mean ± SD; Dunnett's test).

F. Schematic diagram of WT- or H517D-FUS expression vectors; CMV, CMV promoter sequence; 3xFLAG, 3xFLAG tag sequence.

G. Representative image of immunocytochemistry for SG in 293T cells transfected WT- or H517D-FUS expression vector. FLAG-stained cells expressed transfected FUS and H517D-FUS co-localized with G3BP. The scale bars represent 50 µm.

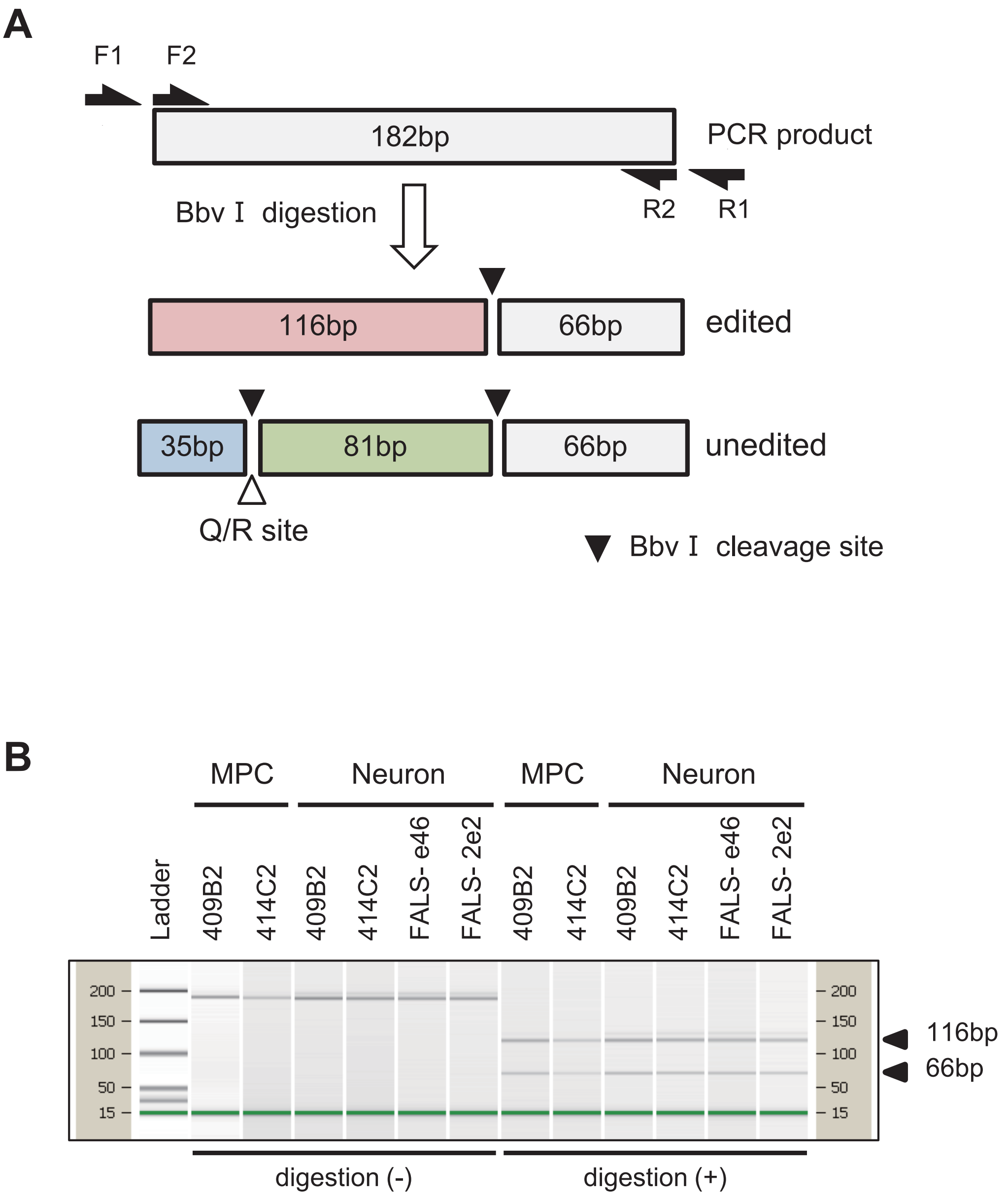

Figure S6. RNA editing analysis in AMPA receptor GluA2 subunit (Related to Figure 7)

A. Schematic diagram of the protocols for detecting the editing efficiency at the Q/R site of GluA2. Open bars represent nested PCR products. Intrinsic BbvI recognition sites are indicated by vertical solid arrowheads. The sizes of the DNA fragments generated by restriction digestion are indicated. Q, glutamine; R, arginine.

B. The gel-like image produced by the 2100 Bioanalyzer. All samples showed 116 bp and 66 bp bands with digestion.

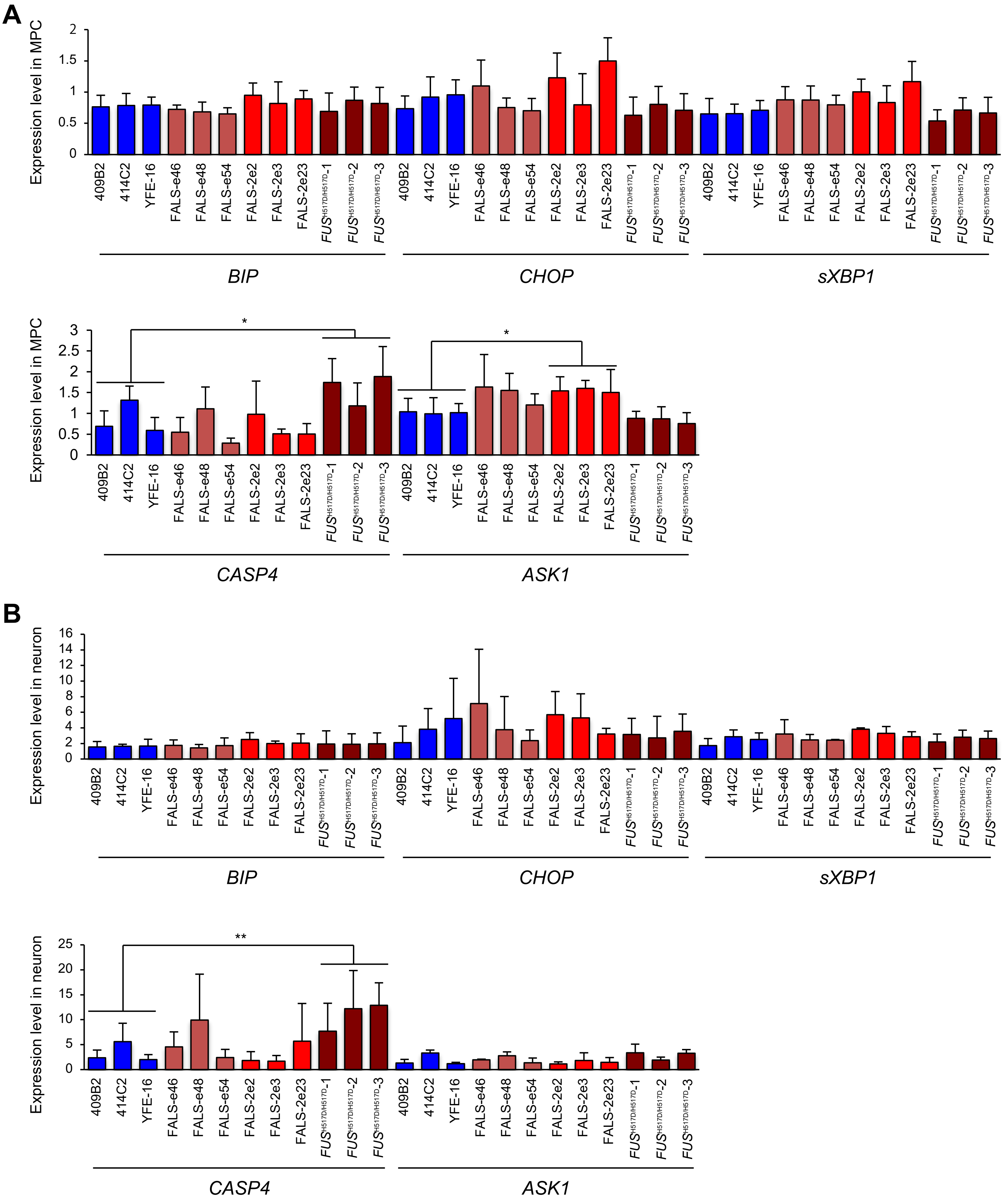

Figure S7. ER stress related gene expression (Related to Figure 7)
Quantitative RT-PCR analysis of the expression of ER stress marker genes in iPSC-derived MPCs (A) and neurons (B). The data are presented as the mRNA copy numbers for each gene divided by those for *ACTB*. (n = 3 independent experiments; mean ± SD; *p<0.05, ** p<0.01; Dunnett's test)

Table S1. Clinical information

Control or FALS	Cell line	Sex	Race	Age	Diagnosis
Control	409B2	Female	Caucasian	36	Healthy
	414C2				
	YFE-16	Male	Japanese	24	Healthy
FALS1	FALS-e46	Male	Japanese	39	Familial ALS
	FALS-e48				
	FALS-e54				
FALS2	FALS-2e2	Male	Japanese	42	Familial ALS
	FALS-2e3				
	FALS-2e23				

Table S2. Overlapping genes between Exon array and FUS CLIP-seq

FC	Stat ebayes	Gene symbol	Gene description
2.260	-5.087	NETO1	neuropilin (NRP) and tolloid (TLL)-like 1
1.980	-5.571	NFASC	neurofascin homolog (chicken)
1.965	-6.703	ST18	suppression of tumorigenicity 18 (breast carcinoma) (zinc finger protein)
1.817	-5.424	CADPS	Ca++-dependent secretion activator
1.684	-6.771	CNTNAP5	contactin associated protein-like 5
1.630	-6.717	SYT4	synaptotagmin IV
1.626	-6.190	LRP1B	low density lipoprotein receptor-related protein 1B | low density lipoprotein-related protein 1B (deleted in tumors)
1.599	-4.963	ANK2	ankyrin 2, neuronal
1.568	-5.448	PDE8B	phosphodiesterase 8B
1.522	-6.164	SNAP91	synaptosomal-associated protein, 91kDa homolog (mouse)
1.514	-5.846	FUT9	fucosyltransferase 9 (alpha (1,3) fucosyltransferase)
1.511	-6.688	ELAVL3	ELAV (embryonic lethal, abnormal vision, Drosophila)-like 3 (Hu antigen C)
1.504	-6.608	GNAO1	guanine nucleotide binding protein (G protein), alpha activating activity polypeptide O
1.436	-4.508	FMN2	formin 2
1.320	-5.692	SLC36A1	solute carrier family 36 (proton/amino acid symporter), member 1
1.313	-4.574	NAPB	N-ethylmaleimide-sensitive factor attachment protein, beta
1.300	-6.983	LRRC8B	leucine rich repeat containing 8 family, member B
1.290	-4.677	PREX1	phosphatidylinositol-3,4,5-trisphosphate-dependent Rac exchange factor 1
1.268	-6.804	SGIP1	SH3-domain GRB2-like (endophilin) interacting protein 1
1.262	-4.765	PSD3	pleckstrin and Sec7 domain containing 3
1.251	-5.125	DST	dystonin
1.206	-4.988	PPA2	pyrophosphatase (inorganic) 2
0.807	5.220	RPAIN	RPA interacting protein

Table S3. Exon array correlation graph

	409 B2	414 C2	YFE-16	FALS-e46	FALS-e48	FALS-e54	FALS-2e2	FALS-2e3	FALS-2e23
409 B2	1	0.9744	0.9761	0.9761	0.9708	0.9754	0.9727	0.9746	0.9774
414 C2	0.9744	1	0.9735	0.9723	0.9746	0.9724	0.9765	0.9782	0.9756
YFE-16	0.9761	0.9735	1	0.9748	0.9716	0.9748	0.9766	0.9803	0.9823
FALS-e46	0.9761	0.9723	0.9748	1	0.9792	0.9813	0.9776	0.9789	0.9796
FALS-e48	0.9708	0.9746	0.9716	0.9792	1	0.9775	0.9779	0.9784	0.976
FALS-e54	0.9754	0.9724	0.9748	0.9813	0.9775	1	0.976	0.978	0.9787
FALS-2e2	0.9727	0.9765	0.9766	0.9776	0.9779	0.976	1	0.9808	0.9798
FALS-2e3	0.9746	0.9782	0.9803	0.9789	0.9784	0.978	0.9808	1	0.9837
FALS-2e23	0.9774	0.9756	0.9823	0.9796	0.976	0.9787	0.9798	0.9837	1

Table S4. Antibody list

Antigen	Supplier	Cat No	Dilution ratio
Cleaved-CASPASE3	Cell Signaling Technology	9661	1:500
FUS	Bethyl Laboratories	A300-293A	1:1000
G3BP	BD Bioscience	611126	1:500
G3BP	Life Technologies	PA1-27843	1:1000
GFP	MBL	598	1:500
GLUR1	Millipore	04-855	1:500
HB9	DSHB	81.5C10	1:150
ISLET1	DSHB	39.4D5	1:1000
MAP2	Sigma	M4403	1:1000
OLIG2	R&D Systems	AF2418	1:500
SMI32	Covance	SMI-32P	1:2500
SOX2	Abcam	ab59776	1:200
SOX17	R&D Systems	AF1924	1:500
SSEA4	Abcam	ab16287	1:500
TRA-1-60	Millipore	MAB4360	1:500
VGLUT1	Synaptic Systems	135-303	1:1000
αSMA	Sigma	A2547	1:500
βIII-TUBULIN	Sigma	T8660	1:1000
βIII-TUBULIN	Covance	MMS-435P	1:2000

Table S5. Primer list

Gene	Forward	Reverse	Assay
FUS	ATGGCCTCAAACGATTATACCCA	GTAACTCTGCTGTCCGTAGGG	qPCR
FUS	GGGGCAACTTTGTATAATAAAGTTGCC GGAAATCCTATCAAGGTCTC (B3-5'FUS-Fw)	TTCGTATAATGTATGCTATACGAAGTTAT TCTTATCTCAAGTGGGTCTAC (LoxP-5'FUS-Rv)	TALEN
FUS	GGGGCAACTTTGTATAATAAAGTTGCC GGAAATCCTATCAAGGTCTC (B3-5'FUS-Fw)	GGGCTGCTTTTTTGTACAAACTTGATAA CTTCGTATAATGTATGCTATAC (B1r-LoxP-Rv)	TALEN
FUS	TTCGTATAGCATACATTATACGAAGTTAT GCTGGTAACTCAAATATAATG (PxoL-3'FUS-Fw)	GGGGCAACTTTGTATAGAAAAGTTGTC TTGGGTTAATGTTACGCTCT (B4-3'FUS-Rv)	TALEN
FUS	GGGCAGCTTTCTTGTACAAAGTGGATA ACTTCGTATAGCATACATTATAC (B2r-PxoL-Fw)	GGGGCAACTTTGTATAGAAAAGTTGTC TTGGGTTAATGTTACGCTCT (B4-3'FUS-Rv)	TALEN
FUS	GGGGCAAGTTTGTACAAAAAAGCAGA ATTCTACCGGGTAGGGGA (B1-EcoR1-pPGK-F')	GGGGCCACTTTGTACAAGAAAGCTGC TGCAGCCCTCGACTCTAG (B2-XbaI-PGKpA-R)	TALEN
FUS	GAAAGGCACGCTTCTCTTGTATTTTCG GAT (5'FUS-PCR-Fw)	TGCTGTCCATCTGCACGAGACTAGTGA G (PGKP-Rv)	PCR genotyping
FUS	CGAGCGGGTCACCGAGCTGCAAGAAC TC (PuroR-Fw)	TGGCAGCTGTCTTACAAACCAAGTTCG AAA (3'FUS-PCR-Rv)	PCR genotyping
FUS	GAAAGGCACGCTTCTCTTGTATTTTCG GAT (5'FUS-PCR-Fw)	TGGCAGCTGTCTTACAAACCAAGTTCG AAA (3'FUS-PCR-Rv)	genomic PCR
FUS	CCGGGGTGGTGGGGACAGAGGTGG (Seq-FUS-Fw)	TCATTTGGCCTTCTCCCCGAACACT (Seq-FUS-Rv)	Sequence
ASK1	CTGCATTTTGGGAAACTCGACT	AAGGTGGTAAAACAAGGACGG	qPCR
SLITRK4	CCTGATTTCTTCGACAAATGCAG	TCTCACAGTTGACATAGAGCACA	qPCR
ALCAM	TCCTGCCGTCTGCTCTTCT	TTCTGAGGTACGTCAAGTCGG	qPCR
NEUROD4	ACCAGGTACTTATGGGATGCT	AAGGCGAGCTTTGGTCATCTT	qPCR
ONECUT2	GGAATCCAAAACCGTGGAGTAA	CTCTTTGCGTTTGCACGCTG	qPCR
NETO1	TGCAGTGTGGAACTTGGACAA	TGGAGCGGCTTCTATGATGTAG	qPCR
ST18	CAAACCACCTAGAGTCCCAAAG	ACACCTGTTCTCACAAGGGATA	qPCR
CADPS	TCAGATGTCGTGCTGTCTTTC	TATACGATGCGATTTGGAGCC	qPCR
SYT4	ATGGGATACCCTACACCCAAAT	TCCCGAGAGAGGAATTAGAACTT	qPCR
RSU1	GCTGAGGACCAGTTGTGTGA	CGTCTTAGGGGCTACCTTCC	RT-PCR
RPH3AL	GCAAACCCACTGATCATTCC	TCCCAGTGATTCAGCTCCTC	RT-PCR
EFCAB13	GGAAAAGGAAATGCTGTCTAACC	ATCCCCAATATCCACCATGT	RT-PCR
OCT3/4 CDS	CCCCAGGGCCCCATTTTGGTACC	ACCTCAGTTTGAATGCATGGGAGAGC	qPCR
OCT3/4 pla	CATTCAAACTGAGGTAAGGG	TAGCGTAAAAGGAGCAACATAG	qPCR
KLF4 CDS	ACCCATCCTTCCTGCCCGATCAGA	TTGGTAATGGAGCGGCGGGACTTG	qPCR
KLF4 pla	CCACCTCGCCTTACACATGAAGA	TAGCGTAAAAGGAGCAACATAG	qPCR
SOX2 CDS	TTCACATGTCCCAGCACTACCAGA	TCACATGTGTGAGAGGGGCAGTGTGC	qPCR
SOX2 pla	TTCACATGTCCCAGCACTACCAGA	TTTGTTTGACAGGAGCGACAAT	qPCR
L-MYC CDS	GCGAACCCAAGACCCAGGCCTGCTCC	CAGGGGGTCTGCTCGCACCGTGATG	qPCR
L-MYC pla	GGCTGAGAAGAGGATGGCTAC	TTTGTTTGACAGGAGCGACAAT	qPCR
LIN28 CDS	AGCCATATGGTAGCCTCATGTCCGC	TCAATTCTGTGCCTCCGGGAGCAGGG TAGG	qPCR
LIN28 pla	AGCCATATGGTAGCCTCATGTCCGC	TAGCGTAAAAGGAGCAACATAG	qPCR
oriP	TTCCACGAGGGTAGTGAACC	TCGGGGGTGTTAGAGACAAC	genomic PCR
OCT4 endogenous	AGTTTGTGCCAGGGTTTTTG	ACTTCACCTTCCCTCCAACC	genomic PCR
BIP	TGTTCAACCAATTATCAGCAAACTC	TTCTGCTGTATCCTCTTCACCAGT	qPCR
CHOP	AGAACCAGGAAACGGAAACAGA	TCTCCTTCATGCGCTGCTTT	qPCR
spliced XBP-1	CTGAGTCCGCAGCAGGTGCAG	ATCCATGGGGAGATGTTCTGG	qPCR
CASP4	GAAACTCCAAGGGCCAAAGC	TCCATTTTCAATTGCCAGGAA	qPCR
GluR2	TCTGGTTTTCCTTGGGTGCC	AGATCCTCAGCACTTTCG	1st PCR
GluR2	GGTTTTCCTTGGGTGCCTTTAT	ATCCTCAGCACTTTCGATGG	nested PCR
ACTB	TGAAGTGTGACGTGGACATC	GGAGGAGCAATGATCTTGAT	PCR & qPCR

Supplemental Experimental Procedures

TALEN-directed mutagenesis

Platinum TALEN-expression vectors were constructed by modified Golden Gate-mediated method as previously described (Sakuma et al., 2013) with some modifications. Briefly, DNA-binding repeats were assembled with the two-step Golden Gate reaction using the Platinum Gate TALEN Kit (Addgene, Kit #1000000043). After the repeat assembly, the CMV promoter of the constructed TALEN vector was replaced with the EF1α promoter. . In order to target upstream region of splicing acceptor site of *FUS* intron 14, a pair of TALENs with following target sequence was designed and constructed (Figure S3A), 5'-TACTCGCTGGGTTAggaggggcagataggATATCTAGGCTTGGAGA-3' (Uppercase and underlined: Sequence binding to TALE-domain of TALENs, Uppercase without underline: T base binding to N-terminal domain of TALE, lowercase: spacer sequence cut by FokI-domain) and was used in farther experiment. In order to introduce H517D mutation in *FUS* exon 15 of control iPSC line, 409B2, a targeting donor plasmid with mutant exon 15 in 3' arm (Figure S3A) was constructed by Multisite Gateway-based method. Either 5' or 3' arm with appropriate *att*B signals at both ends and a *lox*P signal at either side was amplified by 2-step PCR using following PCR primers with additional recombination signals (oriented in the 5' to the 3' direction) and FALS patient genomic DNA with the mutant *FUS* gene as a template. For 5' arm, first with B3-5'FUS-Fw and LoxP-5'FUS-Rv, second with B3-5'FUS-Fw and B1r-LoxP-Rv. For 3' arm, first with PxoL-3'FUS-Fw and B4-3'FUS-Rv, second with B2r-PxoL-Fw and B4-3'FUS-Rv. Then the 5' and 3' arm fragments were cloned into pDONR P3-P1r (Sone et al., 2012) or pDONR-P2r-P4 (Sone et al., 2012), respectively by BP reaction. A positive-negative selection marker cassette, PGK-Puro^R-ΔTK (PurTK), was amplified by PCR using primers, B1-EcoRI-pPGK-F and B2-XbaI-PGKpA-R, and a plasmid vector, PB-TET-PH (kindly provided by Dr. Yuhki Nakatake, Keio University) as a template. Then it was cloned into pDONR201 (Life Technologies) by BP reaction. All the three fragments were confirmed by Sanger sequencing and assembled into pUC-DEST-R3R4 (Sone et al., 2012) by multisite LR reaction. Resulted plasmid clone, pUC-5'3'PDS-FUS(H517D) was used as the targeting donor plasmid (Figure S3A).

TALEN-mediated genome editing of iPSCs

For generating isogenic mutant lines, a control iPSC line, 409B2 was cultured under feeder-free condition using StemFit AK03 (Ajinomoto) and a recombinant laminin-511 E8 fragment, iMatrix-511 (Nippi) as previously described (Nakagawa et al., 2014). Cells were treated with Y27632 (Sigma) at 10 µM overnight before gene transfer. After being dissociated into single cells using TrypLESelect (Thermo Fisher), the cells were divided into 1×10^6 cells and centrifuged at $200\times g$ for 5 min. The supernatant was removed and the pellet was resuspended into 100 µl of Opti-MEM (Thermo Fisher) containing 10 µM Y27632, 5 µg each of left and right TALEN-expressing plasmids and 10 µg of targeting donor plasmid. Electroporation was performed using NEPA21 electroporator (Nepagene) with modifying the previously reported method (Li et al., 2015) with 275 V poring pulse voltage and 0.5 ms poring time. Cells were dispersed into 3 mL of StemFit AK03 containing 10 µM Y27632 and plated on an iMatrix-511-coated 6-cm dish (D=0). The medium was changed to StemFit AK03 without Y27632 every other day since the next day (D=1) until colony-picking. To obtain cells with homologous recombinant of PGK-PurTK into *FUS* locus, two times selection was performed with adding 1 µg/ml of puromycin to media for 24 h. Around D=14 to D=20, each single puromycin-resistant iPSC colony was picked up into 10 µl of TrypLESelect with Y27632 in one well of 96-well plate. After incubation for a few minutes at room temperature, 90µl of StemFit AK03 with Y27632 was added to the well and cells were dissociated roughly by pipetting and plated on one well of iMatrix-511-coated 24-well plate filled with 400 µl of StemFit AK03 with Y27632. The picked-up iPSCs were maintained in StemFit AK03 without Y27632 from the next day until the first passage. One quarter of the cells were passaged and the rest were harvested for preparing genomic DNA using KAPA Express Extract kit (Nippon Genetics). PCR genotyping for identification of knock-in clone was performed using following primers (oriented in the 5' to the 3' direction);
5'FUS-PCR-Fw and PGKP-Rv for detection of 5' border of knock-in allele and PuroR-Fw and 3'FUS-PCR-Rv for detection of 3' border of knock-in allele, 5'FUS-PCR-Fw and 3'FUS-PCR-Rv for detection of intact allele. The PCR fragments from knock-in clones were purified by PEG precipitation and confirmed for gene modification by Sanger sequencing using following primers; Seq-FUS-Fw and Seq-FUS-Rv. The established knock-in iPSC clones with successful gene modification was maintained by feeder-free method in 6-well plate.

Next, to obtain PGK-PurTK cassette free cells, the cells were infected with AdEFNCre-4FVF (kindly provided by Dr. Yumi Kanegae, the University of Tokyo), which is an EF-1α promoter derivative of a Cre recombinase expressing adenoviral vector, AxCANCre (Kanegae et al., 1995) (D=0). Two days after infection (D=2), 2.5 µg/ml of ganciclovir (Sigma) at final concentration was added to the media for selecting cells that is free from PGK-PurTK cassette flanked by two *lox*P signals. After selections (D=14 to D=20), each single ganciclovir-resistant iPSC colony was picked up as described above. PCR genotyping and Sanger sequencing of the PCR fragments were performed using the primers described above in order to confirm correct excision of the cassette and site-directed gene modification of H517D mutation derived from patient *FUS* gene.

RNA Editing Analysis

Analysis of RNA editing on AMPA receptor subunit GluA2 was performed as previously described with slight modifications (Kawahara et al., 2004; Nishimoto et al., 2008). Briefly, genomic DNA was amplified using 1st PCR primers, and nested PCR was conducted using the first PCR product as template. The nested PCR products were digested with BbvI restriction enzyme (New England Biolabs). The digested PCR products were analyzed using a Bioanalyzer 2100 (Agilent Technologies).

Supplemental References

Akiyama T, Warita H, Kato M, Nishiyama A, Izumi R, Ikeda C, Kamada M, Suzuki N, Aoki M. (2016). Genotype-phenotype relationships in familial ALS with FUS/TLS mutations in Japan. Muscle Nerve. doi: 10.1002/mus.25061.

Kanegae, Y., Lee, G., Sato, Y., Tanaka, M., Nakai, M., Sakaki, T., Sugano, S., and Saito, I. (1995). Efficient gene activation in mammalian cells by using recombinant adenovirus expressing site-specific Cre recombinase. Nucleic Acids Res 23, 3816-3821.

Li, H. L., Fujimoto, N., Sasakawa, N., Shirai, S., Ohkame, T., Sakuma, T., Tanaka, M., Amano, N., Watanabe, A., Sakurai, H., et al. (2015). Precise correction of the dystrophin gene in duchenne muscular dystrophy patient induced pluripotent stem cells by TALEN and CRISPR-Cas9. Stem Cell Reports 4, 143-154.

Nakagawa, M., Taniguchi, Y., Senda, S., Takizawa, N., Ichisaka, T., Asano, K., Morizane, A., Doi, D., Takahashi, J., Nishizawa, M., et al. (2014). A novel efficient feeder-free culture system for the derivation of human induced pluripotent stem cells. Sci Rep 4, 3594.

Nishimoto, Y., Yamashita, T., Hideyama, T., Tsuji, S., Suzuki, N., and Kwak, S. (2008). Determination of editors at the novel A-to-I editing positions. Neurosci. Res. *61*, 201–206.

Sakuma, T., Ochiai, H., Kaneko, T., Mashimo, T., Tokumasu, D., Sakane, Y., Suzuki, K., Miyamoto, T., Sakamoto, N., Matsuura, S., and Yamamoto, T. (2013). Repeating pattern of non-RVD variations in DNA-binding modules enhances TALEN activity. Sci Rep 3, 3379.

Sone, T., and Imamoto, F. (2012). Methods for constructing clones for protein expression in mammalian cells. Methods Mol Biol 801, 227-250.
