# Supplementary material for: Establishment of In Vitro FUS-Associated Familial Amyotrophic Lateral Sclerosis Model Using Human Induced Pluripotent Stem Cells
Source: Stem Cell Reports. 2016 Mar 17;6(4):496–510. doi: 10.1016/j.stemcr.2016.02.011 (PMC4834049; doi:10.1016/j.stemcr.2016.02.011)
Supplement: Document S2. Article plus Supplemental Information [file mmc2.pdf]

Stem Cell Reports
Article
OPEN ACCESS

Establishment of In Vitro FUS-Associated Familial Amyotrophic Lateral Sclerosis Model Using Human Induced Pluripotent Stem Cells

Naoki Ichiyanagi,[1,8] Koki Fujimori,[1,8] Masato Yano,[1,2,*] Chikako Ishihara-Fujisaki,[1] Takefumi Sone,[1] Tetsuya Akiyama,[3] Yohei Okada,[1,4] Wado Akamatsu,[5] Takuya Matsumoto,[1] Mitsuru Ishikawa,[1] Yoshinori Nishimoto,[1] Yasuharu Ishihara,[1] Tetsushi Sakuma,[6] Takashi Yamamoto,[6] Hitomi Tsuiji,[7] Naoki Suzuki,[3] Hitoshi Warita,[3] Masashi Aoki,[3] and Hideyuki Okano[1,*]
[1]Department of Physiology, School of Medicine, Keio University, 35 Shinanomachi, Shinjuku-ku, Tokyo 160-8582, Japan
[2]Division of Neurobiology and Anatomy, Graduate School of Medical and Dental Sciences, Niigata University, 1-757, Asahimachidori, Chuo-ku, Niigata 951-8510, Japan
[3]Department of Neurology, Tohoku University Graduate School of Medicine, 1-1 Seiryo-machi, Aoba-ku, Sendai, Miyagi 980-8574, Japan
[4]Department of Neurology, Aichi Medical University School of Medicine, 1-1 Yazako Karimata, Nagakute, Aichi 480-1195, Japan
[5]Center for Genomic and Regenerative Medicine, Graduated School of Medicine, Juntendo University, 2-1-1 Hongo, Bunkyo-ku, Tokyo 113-8421, Japan
[6]Department of Mathematical and Life Sciences, Graduate School of Science, Hiroshima University, 1-3-1 Kagamiyama, Higashihiroshima, Hiroshima 739-8526, Japan
[7]Department of Biomedical Science, Graduate School of Pharmaceutical Sciences, Nagoya City University, 3-1 Tanabe-dori, Mizuho-ku, Nagoya, Aichi 467-8603, Japan
[8]Co-first author
*Correspondence: myano@med.niigata-u.ac.jp (M.Y.), hidokano@a2.keio.jp (H.O.)
http://dx.doi.org/10.1016/j.stemcr.2016.02.011
This is an open access article under the CC BY license (http://creativecommons.org/licenses/by/4.0/).

SUMMARY

Amyotrophic lateral sclerosis (ALS) is a late-onset motor neuron disorder. Although its neuropathology is well understood, the cellular and molecular mechanisms are yet to be elucidated due to limitations in the currently available human genetic data. In this study, we generated induced pluripotent stem cells (iPSC) from two familial ALS (FALS) patients with a missense mutation in the *fused-in sarcoma* (*FUS*) gene carrying the heterozygous FUS H517D mutation, and isogenic iPSCs with the homozygous FUS H517D mutation by genome editing technology. These cell-derived motor neurons mimicked several neurodegenerative phenotypes including mis-localization of FUS into cytosolic and stress granules under stress conditions, and cellular vulnerability. Moreover, exon array analysis using motor neuron precursor cells (MPCs) combined with CLIP-seq datasets revealed aberrant gene expression and/or splicing pattern in FALS MPCs. These results suggest that iPSC-derived motor neurons are a useful tool for analyzing the pathogenesis of human motor neuron disorders.

INTRODUCTION

Amyotrophic lateral sclerosis (ALS) is a neurodegenerative disease resulting in the selective death of motor neurons (Cleveland and Rothstein, 2001). ALS symptoms are associated with muscle weakness and paralysis and approximately 80% of ALS patients die within 3–5 years after the onset of these symptoms. The prevalence of ALS is two per 100,000 people per year (Bruijn et al., 2004) and approximately 10% of patients have a familial history of the disease (Gros-Louis et al., 2006). Familial ALS (FALS) is identified by mutations in several genes, including *SOD1*, *TARDBP* and *FUS* (Chen et al., 2013).

Several efforts including animal and in vitro culture models have been undertaken to understand the pathogenic mechanism of ALS. In animal models, neurobiological phenotypes of ALS are observed, which are due to multiple pathogenic mechanisms, including protein degradation, oxidative stress, inflammation, paraspeckle formation, mitochondrial dysfunction and apoptotic pathways (Lanson and Pandey, 2012; Nishimoto et al., 2013; Robberecht and Philips, 2013; Tsao et al., 2012). In addition, the use of recently developed induced pluripotent stem cell (iPSC) technologies also enables understanding of the disease pathogenesis (Mattis and Svendsen, 2011; Okano and Yamanaka, 2014). Indeed, iPSCs have been generated from ALS patients with mutations in SOD1 (Chestkov and Vasilieva, 2014; Dimos et al., 2008), TDP-43 (Bilican et al., 2012; Egawa et al., 2012), C9ORF72 (Almeida et al., 2013; Sareen et al., 2013) and recent publications of FUS (Lenzi et al., 2015; Liu et al., 2015; Di Salvio et al., 2015) suggest a useful tool for pursuing the cellular pathogenesis and mechanism underlying FALS.

FUS, also known as Translocated in Liposarcoma (TLS), is a DNA/RNA-binding protein containing a glycine-rich region, an RNA recognition motif and a nuclear localization signal (Lattante et al., 2013; Yang et al., 2010). In FALS, more than 50 mutations in the *FUS* gene have been reported (Lattante et al., 2013). Some mutant FUS proteins form nuclear/cytosolic protein aggregations that shift from the nucleus to the cytoplasm (Dormann et al., 2010; Suzuki et al., 2012; Vance et al., 2013; Zhou et al., 2013). This sequestration of FUS into aggregations is thought to be a potential cue for the initiation of motor neuron degeneration.

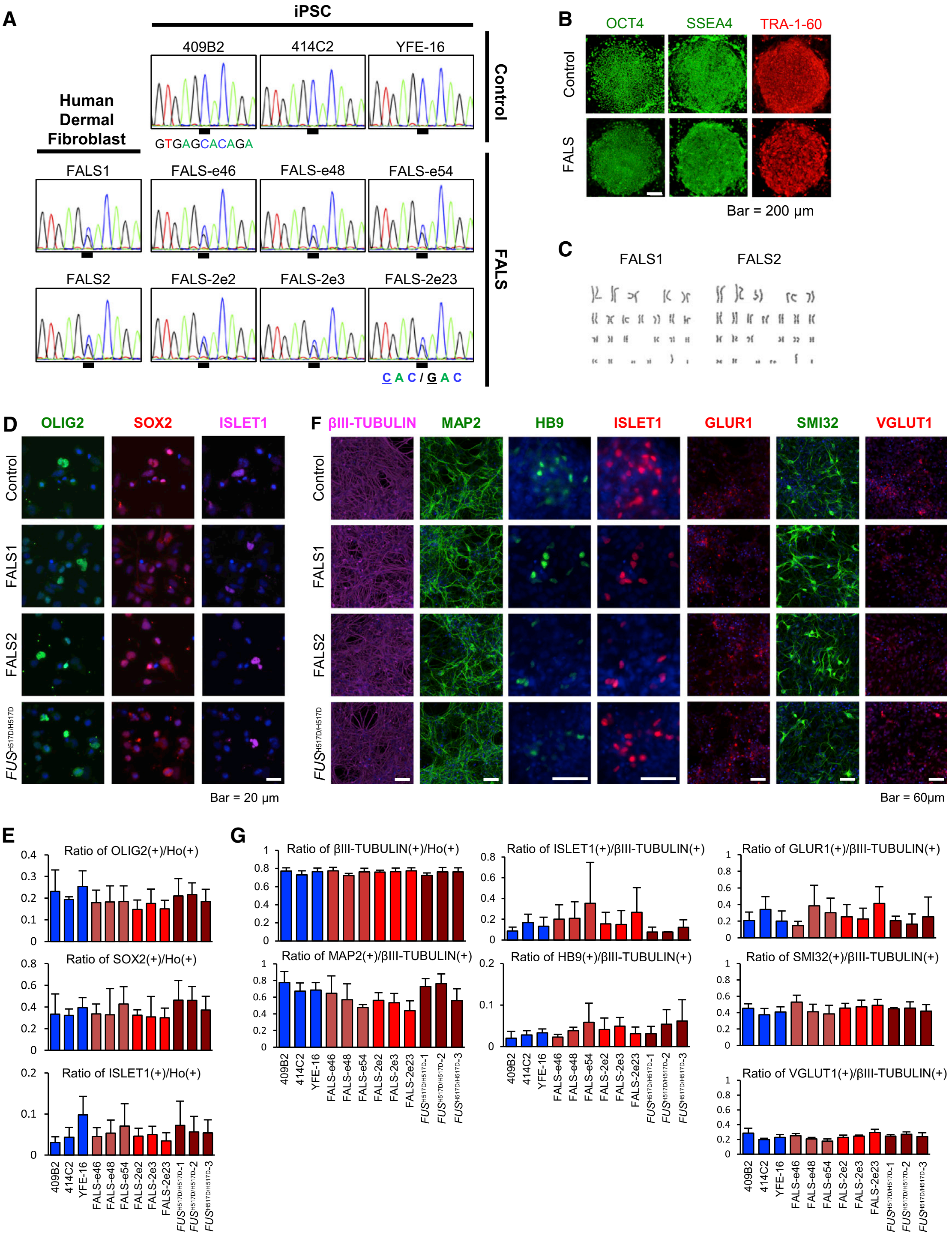

Figure 1. Characterization of iPSCs and Differentiation into Motor Neurons

(A) Human dermal fibroblasts from two ALS patients who carried the FUS H517D heterozygous mutation (C-to-G heterozygous mutation); the mutation was maintained in the generated iPSCs.

(B) Representative image of immunochemical analysis of pluripotent markers, OCT4, SSEA4 and TRA-1-60. Control, YFE-16; FALS, FALS-2e2. The same images are shown in Figure S1B. The scale bar represents 200 μm.

(C) Representative karyotypes of the generated FALS1 and FALS2 iPSC lines are shown.

(legend continued on next page)

In the present study, we generated iPSCs from two ALS patients carrying the FUS H517D mutation, healthy volunteers and isogenic iPSCs with FUS H517D mutation using the TALEN system and investigated the multifaceted cellular phenotypes of their motor neurons in vitro. We differentiated these iPSCs into the motor neuron cell lineage and observed significant mis-localization of mutant FUS to the cytosol and the accumulation of FUS in stress granules under various stresses, which has never been reported for wild-type FUS protein. Furthermore, using two comprehensive analyses, exon array combined with previous CLIP-seq data (Lagier-Tourenne et al., 2012), we identified transcripts showing aberrant gene expression, which may be involved in FUS-dependent pathology, even in motor neuron precursor cells (MPCs) derived from FALS iPSCs. The subsequent analysis using the IN Cell Analyzer revealed an increase in neuronal cell death and a decrease in neurite length in FALS iPSC-derived HB9-positive motor neurons compared with controls under stress conditions. In contrast, in our analysis on βIII-TUBULIN-positive neurons, there were nearly no changes in FUS mis-localization, FUS accumulation in stress granules, occurrence of neuronal cell death, or neurite length. Thus, the present report describes the in vitro modeling of human ALS with the *FUS* mutation to uncover the pathogenetic history of this disease.

RESULTS

Generation of FUS-ALS-iPSCs and Differentiation into Motor Neurons

We generated FALS iPSCs derived from skin fibroblasts isolated from two FALS patients (Figure S1A and Table S1), both with a point mutation in the gene encoding the C-terminal domain of the FUS protein (Akiyama et al., 2016). To reprogram the skin cells into FUS-ALS-iPSCs, we used episomal vectors carrying *OCT4*, *SOX2*, *KLF4*, *L-MYC*, *LIN28* and *p53* shRNA. The two ALS patients had a heterozygous C-to-G transition at nucleotide 1,550 (c.1550C>G) in the exon 15 coding sequence of the *FUS* gene (hereafter referred to as patient-1, FALS-e46, FALS-e48, and FALS-e54; and patient-2, FALS-2e2, FALS-2e3, and FALS-2e23). We confirmed that FALS iPSCs also harbored the heterozygous C-to-G point mutation in the *FUS* gene (Figure 1A) and had no other mutations in the *FUS* gene (data not shown). This point mutation causes a single amino acid substitution (histidine to aspartic acid) at amino acid position 517 in the C-terminal domain of the FUS protein, termed H517D. Furthermore, we established another control iPSC line, YFE-16 (Shimojo et al., 2015), in addition to the two control human iPSC lines, 409B2 and 414C2, which were established previously (Table S1) (Okita et al., 2011). These three control lines were derived from two individuals who were diagnosed as clinically healthy and did not have the C-to-G point mutation in the *FUS* gene (Figure 1A). Importantly, these iPSC lines showed the typical morphology of colonies similar to human embryonic stem cell lines, based on the expression of pluripotent stem cell markers (SSEA4, OCT4, and TRA-1-60) by immunocytochemical analysis (Figures 1B and S1B; control and FALS in Figure 1B represented YFE-16 and FALS-2e2 respectively and the same images are shown in Figure S1B), normal karyotypes by the G-band staining method (Figure 1C) and no exogenous transgene expression by qRT-PCR (Figure S1C) or *oriP* genomic PCR (Figure S1D). We also confirmed the pluripotency of differentiation potentials into three germ layers (Figure S1E). In addition, we generated isogenic iPSCs with FUS H517D homozygous mutation (hereafter referred to as *FUS*$^{H517D/H517D}$-1, -2, and -3) using the TALEN genome editing systems on 409B2 control iPSCs (Figure S2).

All iPSCs were differentiated into neural lineages including motor neurons based on a previously described protocol but with slight modifications (Bilican et al., 2012; Chambers and Fasano, 2009; Egawa et al., 2012; Hester et al., 2011; Hu and Zhang, 2009; Imaizumi et al., 2015; Nizzardo et al., 2010; Surmacz et al., 2012; Matsumoto et al., 2016). The iPSCs were transferred into a suspension culture to form the neurosphere in neural progenitor maintenance media containing retinoic acid and the hedgehog signaling activator, purmorphamine, to promote the commitment of MPCs. All MPCs were constructed from motor neuron progenitor marker, OLIG2 and SOX2 double-positive cells, and an early marker of motor neuron differentiation, ISLET1 positive cells (Figure 1D). Quantitative analysis for the ratio of marker expression using IN Cell Analyzer revealed that there was no significant difference in the differentiation ratio between control, FALS, and *FUS*$^{H517D/H517D}$ MPCs (Figure 1E). MPCs

(D) Representative image of immunocytochemistry for the neural stem cell marker (SOX2) and motor neuron progenitor markers (OLIG2 and ISLET1). The scale bar represents 20 μm.
(E) Quantitative data of the ratio of each MPC marker-positive cell/Hoechst-positive cell (n = 3 independent experiments; means ± SD; Tukey's test).
(F) Representative image of immunocytochemistry for motor neuron markers (HB9, ISLET1 and SMI32) and other neural markers (βIII-TUBULIN, MAP2, VGLUT1 and GLUR1). The scale bars represent 60 μm.
(G) Quantitative data of the ratio of each marker-positive cell/βIII-TUBULIN-positive cell (n = 3 independent experiments; mean ± SD; Tukey's test).

were passaged twice and then adhered on poly-ornithine/laminin-coated plates. Neurons were differentiated after 20 days in adherent culture. These neuronal cell populations contained motor neurons (i.e., HB9, ISLET1 and SMI32), glutamatergic neurons (i.e., VGLUT1) and glutamate-responsive neurons (i.e., GLUR1) (Figure 1F) and there was no significant difference in the ratio of these markers between control, FALS, and *FUS*$^{H517D/H517D}$ neurons (Figure 1G).

Aberrant Gene Expression in FALS Motor Neuron Progenitor Cells

We performed exon array analysis using an Affymetrix GeneChip Human Exon 1.0 ST Array to analyze RNA profiles in control and FALS-derived MPCs, which efficiently differentiate into motor neurons. We compared 18,738 genes using core probes to observe fold changes (FCs) in gene expression between the three control and six FALS-derived cell types. A scatterplot of FCs showed a very high correlation coefficient of more than 0.9, suggesting our in vitro culture model was stable regarding their transcriptome. Next, we prepared a list of FUS-regulated genes, which were differentially expressed for each cell, by filtering the gene-level signal intensities with Bayes statistics p values <0.001. Of the 159 differently expressed genes we identified, 124 genes were upregulated and 35 genes were downregulated in FALS MPCs as summarized in plots and a heatmap (Figures 2A and 2B). We analyzed the Gene Ontology (GO) terms (Figure 2C) of the genes, including *SLITRK4*, *DST*, *GNAO1*, *ALCAM*, *NFASC*, *NEUROD4* and *ONECUT2* that were aberrantly expressed in FALS-derived cells. We found that these genes were associated with neuron differentiation, neuron development and cell adhesion and were enriched compared with the profiles of control MPCs (Aruga and Mikoshiba, 2003).

We reanalyzed a previous FUS CLIP-seq (crosslinking and immunoprecipitation, followed by high-throughput sequencing) dataset (Lagier-Tourenne et al., 2012) to determine whether this gene regulation is involved in direct regulation of the FUS protein. Among 10,159 FUS CLIP clusters, we defined 1,558 genes with tag number ≥20 and peak height ≥10; approximately 75% were located in intronic sequences (Figure 2D), which is consistent with previously reported FUS binding sites. We found that 23 genes were significantly overlapped using the exon array of 159 genes and potential FUS targets, as well as 1,558 genes based on the CLIP-seq dataset (Figures S3A, S3B, and Table S2). Most of these differently expressed genes were validated by qRT-PCR analysis (Figure 2E), indicating consistency between qRT-PCR and exon array analyses (Table S3).

We also analyzed the alternative splicing changes in control and FALS MPCs by exon array. We identified altered expression of exons between control and FALS MPCs including *RSU1* (Ras suppressor protein 1), *RPH3AL* (rabphilin 3A-like) and *EFCAB13* (EF-hand calcium binding domain 13), which had differences in expression levels on the core probes (Figure 3A). To validate these results by semi-quantitative RT-PCR assay (Figure 3B), we designed the primers flanking the splicing target exons in these genes and validated alternative splicing changes using total RNA obtained from tertiary MPC-specific cells by semi-quantitative RT-PCR analysis. As expected, these three genes showed dramatic splicing changes in FALS MPCs (Figure 3C). To confirm whether the splicing changes between control and FALS-derived cells depend on the mutation of the FUS protein, we analyzed the splicing patterns of these three genes in the MPCs derived from *FUS*$^{H517D/H517D}$-iPSCs carrying the FUS H517D homozygous mutation. We confirmed that differential splicing changes also occur in the *RPH3AL* and *EFCAB13* genes between control and *FUS*$^{H517D/H517D}$ MPCs (Figures 3D and S3C). Semi-quantitative analysis of splicing variants revealed that *FUS*$^{H517D/H517D}$ MPCs express higher levels of the *RPH3AL* 377-bp band than 409B2 control MPCs, but express similar levels of *RSU1* and *EFCAB13* splicing bands (Figure 3E). These results suggest that aberrant gene expressions and/or splicing changes are associated partially with mutant FUS.

Mutant FUS Is Localized in Stress Granules under Stress Conditions

The H517D mutation in FUS lies in a nuclear localization signal (NLS) (Figure 4A). Previous reports revealed that the FUS protein localizes to the nucleus; however, the mutations in the NLS sequences of the FUS protein cause mis-localization in the cytoplasm, even under normal conditions. To address the possibility that the FUS protein from FALS iPSC-derived cells harboring the H517D mutation also mis-localizes to the cytoplasm, we performed immunocytochemical analysis. The data confirmed that there is cytoplasmic mis-localization of the FUS protein in FALS and *FUS*$^{H517D/H517D}$ iPSCs (Figure 4B) and we determined the ratio of cytosolic FUS using IN Cell Analyzer (Figure 4C). The FUS proteins in FALS and *FUS*$^{H517D/H517D}$ iPSC-derived neuronal lineage cells (Figures 4D and 4E) and HB9-positive motor neurons (Figures 4D and 4F) were also mis-localized into cytosol. In addition, we performed biochemical analysis to measure the expression levels of *FUS* mRNA. Control, FALS and *FUS*$^{H517D/H517D}$ MPCs and neurons expressed similar levels of the *FUS* gene (Figure S4).

It has been reported that mutant FUS localizes into cytoplasmic stress granules (SGs) upon various stimuli (Aulas et al., 2012; Bentmann et al., 2012; Vance et al., 2013). SGs are cytosolic structures that transiently form upon exposure of cells to environmental stress, such as heat, oxidative stress, or hypoxia functions in the cellular defense

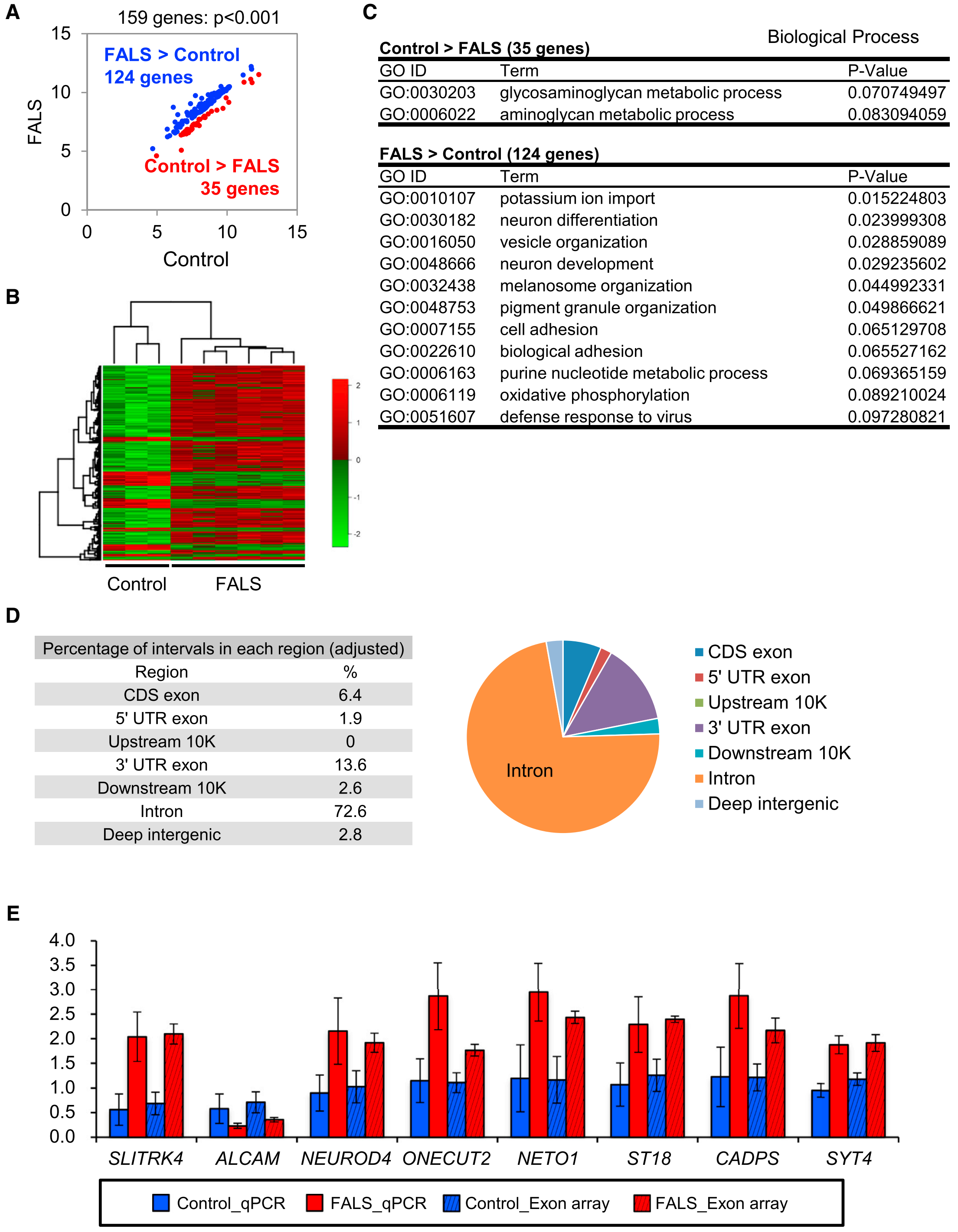

Figure 2. Exon Array Analysis Using MPCs and Comparison with FUS CLIP-Seq

(A) Scatterplot analysis of gene expression using control and FALS MPCs. A total of 124 genes were upregulated (blue) and 35 were downregulated (red) in FALS MPCs compared with control MPCs.

(B) The heatmap of correlation coefficients.

(C) Major GO terms showed both increases and decreases in gene expression in FALS versus control MPCs.

(D) Reanalysis of previously reported CLIP-seq (Lagier-Tourenne et al., 2012).

(E) Quantitative RT-PCR analysis of the expression levels for eight randomly selected genes in control and FALS iPSC-derived MPCs. Solid and hatched bars show qRT-PCR and exon array data, respectively (n = 3–6 independent samples; mean ± SD; Dunnett's test).

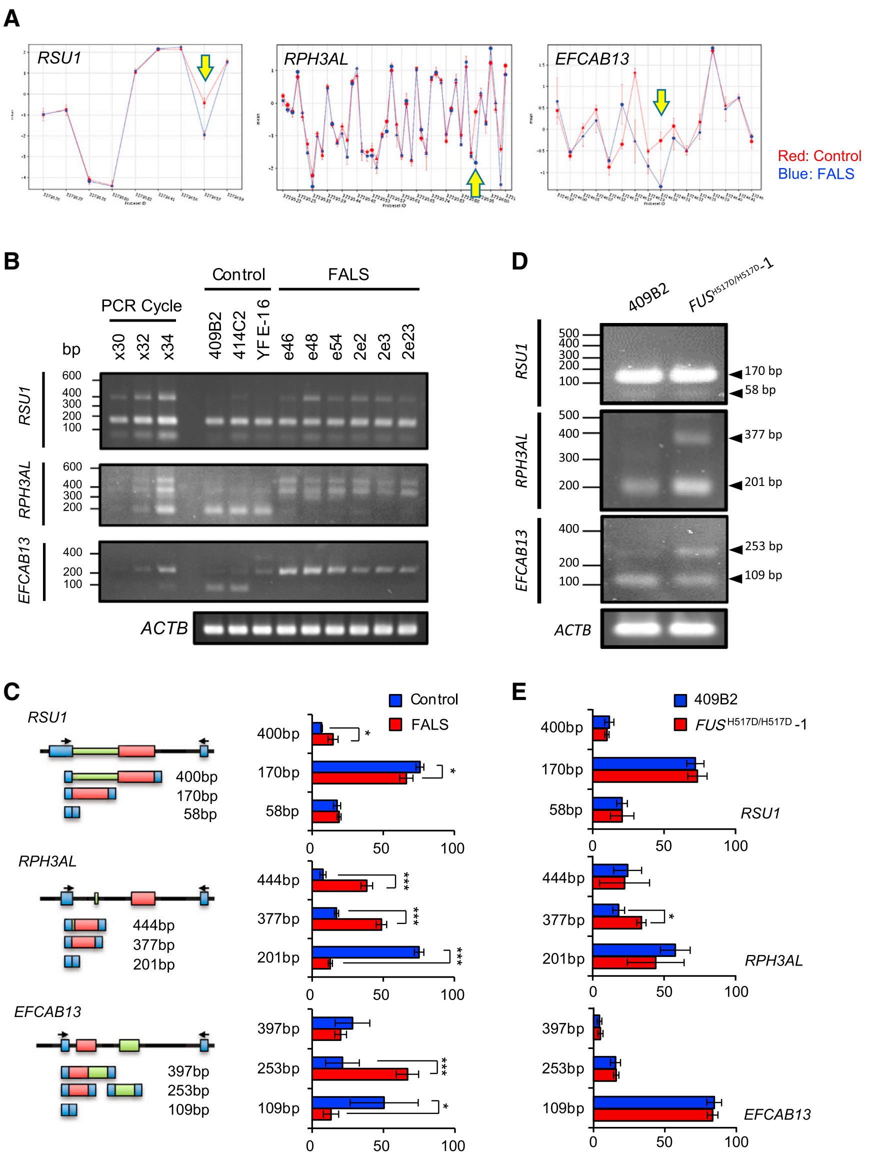

Figure 3. Alternative Splicing Analysis on MPCs

(A) The plots of the expression levels in exon probes. Red and blue lines show control and FALS expression levels, respectively. The yellow arrows show the change points of splicing between controls and FALS in each gene.

(B) RT-PCR of splicing variants in *RSU1*, *RPH3AL* and *EFCAB13* in iPSC-derived MPCs. The PCR cycle validated the PCR cycle numbers.

(C) Schematic figure of alternative splicing in each gene (left) and the measurement of the expression level of each of the spliced bands in (B) (right) (n = 3–6 independent samples; mean ± SD; *p < 0.05; ***p < 0.001; Student's t test).

(D) RT-PCR of splicing variants in *RSU1*, *RPH3AL* and *EFCAB13* in 409B2 and $FUS^{H517D/H517D}$-1 iPSC-derived MPCs.

(E) Expression levels of each of the spliced bands in (D) (n = 3 independent experiments; mean ± SD; *p < 0.05; Student's t test).

against stress, as well as translational repression of a subset of mRNAs (Nishimoto et al., 2010). We induced oxidative stress in iPSCs and iPSC-derived neurons by treatment with 0.5 mM sodium arsenite for 60 min to observe the formation of SGs using anti-G3BP (Ras GTPase-activating protein-binding protein) as a marker of SGs for immunocytochemistry. First we found that FUS proteins from FALS and $FUS^{H517D/H517D}$ iPSCs and iPSC-derived neurons leak and form aggregates in the cytosol, and that their aggregates co-localize with SGs (Figure S5A). In contrast, wild-type FUS remained in the nucleus and did not form G3BP-positive cytoplasmic granules (Figures 5A and 5F). Next, we determined the number of all SGs and FUS-positive SGs per OCT4-positive iPSCs or HB9-positive motor neurons using IN Cell Analyzer. The number of all SGs in iPSCs was unchanged in all lines (Figure 5B); however, the levels of FUS-positive SGs are significantly higher in FALS- and $FUS^{H517D/H517D}$-iPSCs than in controls (Figure 5C). We next examined similar assays in neuronal lineages. Importantly, we confirmed that the number of neuronal cell populations and all SGs in the differentiating cells in our culture was not significantly changed among all the lines (Figures 5D, 5E and 5G). FALS- and $FUS^{H517D/H517D}$-iPSC-derived neuronal lineage cells and HB9-positive motor neurons showed higher levels of FUS-positive SGs than did controls with significant changes (Figures 5H and 5I). We obtained similar results under the condition of 44°C heat shock (Figures S5B–S5E). In addition, FALS iPSC lines expressed both H517D-mutant and normal *FUS*; therefore, we used a transient expression assay to confirm whether the H517D

mutant FUS protein is localized into cytoplasmic SGs under stress conditions. The 293T cell lines were transfected with plasmids encoding either wild-type FUS or H517D mutated FUS (Figure S5F). Expectedly, only the H517D FUS mutant was co-localized with G3BP-positive SGs under the treatment with arsenite but not wild-type FUS, reflecting our observation in FUS iPSC lines (Figure S5G).

Stress Vulnerability in FALS Motor Neurons

While ALS is an adult-onset neurodegenerative disease that specifically targets motor neurons, GO analysis and overlapping genes with CLIP-seq predicted that FALS motor neurons are involved in neural development (Table S2). To address the possibility that FALS iPSC-derived neurons display any defects of neuronal maturation, we measured neurite length on motor neurons. Neural cell populations derived from iPSCs included various neuronal subtypes. Therefore, to label only motor neurons, HB9-venus reporter lentivirus (HB9:Venus) (Shimojo et al., 2015) was infected into the iPSC-derived cells. Motor neurons were visualized by Venus fluorescent protein (Figure 6A); we identified motor neurons labeled by the Venus fluorescent protein with the anti-GFP antibody to determine the length of neurites by IN Cell Analyzer (Figure 6B). As a result, there were no differences between controls, FALS, and *FUS*$^{H517D/H517D}$-derived βIII-TUBULIN-positive neurons under all conditions: unstressed, sodium arsenite treatment and glutamate treatment (Figure 6C). In HB9:Venus-positive motor neurons, however, FALS and *FUS*$^{H517D/H517D}$ neurons showed significantly shorter neurites than controls by treatment with both sodium arsenite and glutamate (Figure 6D). These results suggest that FALS-derived neurons might show not only neuronal maturation (in this case, neurite maintenance) but also cellular vulnerability especially in motor neurons. Therefore, we next pursued the cell viability of FALS-derived neurons and HB9-positive motor neurons under normal and stress conditions by immunostaining with cleaved-CASPASE3, which is a marker for apoptosis (Figure 7A). In all neurons labeled with βIII-TUBULIN, we found significant differences between control and *FUS*$^{H517D/H517D}$ lines; this may be due to homozygous mutation of FUS protein (Figure 7B). However, we were unable to confirm this as a single population of the FALS line also has a significant change. On the other hand, in HB9-positive motor neurons, the changes in the apoptotic cell population were much greater between control and all FALS and *FUS*$^{H517D/H517D}$ lines (Figure 7C). These results suggest that iPSC-derived HB9-positive motor neurons with FUS H517D mutation are vulnerable to stress and even with normal conditions rather than other types of neurons, this phenomenon reflects the ALS-like malady phenotype. Therefore we conclude that our in vitro FALS model is a useful tool to pursue the mechanism underlying the disease phenotype and treatment.

DISCUSSION

In the present study, we established ALS-specific human iPSCs from two patients with a point mutation in the *FUS* gene and isogenic iPSCs with FUS H517D homozygous mutations derived from healthy 409B2 iPSCs using the TALEN genome editing system. In addition, we differentiated into patient derived neurons to observe the disease history of FALS during neuronal differentiation in vitro. FALS-derived motor neuron lineage cells and HB9-positive mature motor neurons showed several ALS-related phenotypes such as neuronal cell death, pathological cellular structure and altered gene regulation, including steady-state transcript levels and alternative splicing. Our in vitro model may thus enable an investigation into the correlations between the molecular pathophysiology of ALS and various cell biological phenomena.

RNA-mediated mechanisms in ALS originated from the first discovery of ubiquitinated TDP43 protein, which is an RNA-binding protein (Neumann et al., 2006). Subsequent studies found the mutation in this gene among FALS (Ricketts et al., 2014; Rutherford et al., 2008; Sreedharan et al., 2008). TDP43-containing protein inclusions in cells ultimately came to be recognized as a pathological hallmark in ALS and frontotemporal lobar degeneration (FTLD). The other RNA-binding protein, the FUS/TLS protein coding gene, also contains potential causative mutations in FALS and in FTLD (Kwiatkowski et al., 2009; Vance et al., 2009). In the present study, the mutant FUS protein was co-localized with the stress marker protein G3BP in our cultured iPSCs, HB9-positive motor neurons derived from them, and fibroblast cell line overexpressing FUS protein responding to stress conditions, arsenite and heat shock (Figures 5F and S5). In general, the stress granule is a mechanism for avoiding stress and has been implicated in the cellular stress defense. These granules are in equilibrium between assembly and disassembly to manage correct gene regulation with cellular conditions. One such pathological hypothesis considers that once this equilibrium is disrupted, these stress granules form irreversible protein inclusions, named "pathological aggregates." In our in vitro model, we observed that FUS mutant proteins localize to SGs in iPSC-derived HB9-positive motor neurons, even under unstressed conditions; the co-localization of FUS and G3BP has not been observed in wild-type cells expressing normal FUS. This suggests the possibility that over-migration of FUS into SGs potentially causes aberrant gene expression and/or splicing, and that these granules may eventually form pathological aggregates, leading to neuronal cell death.

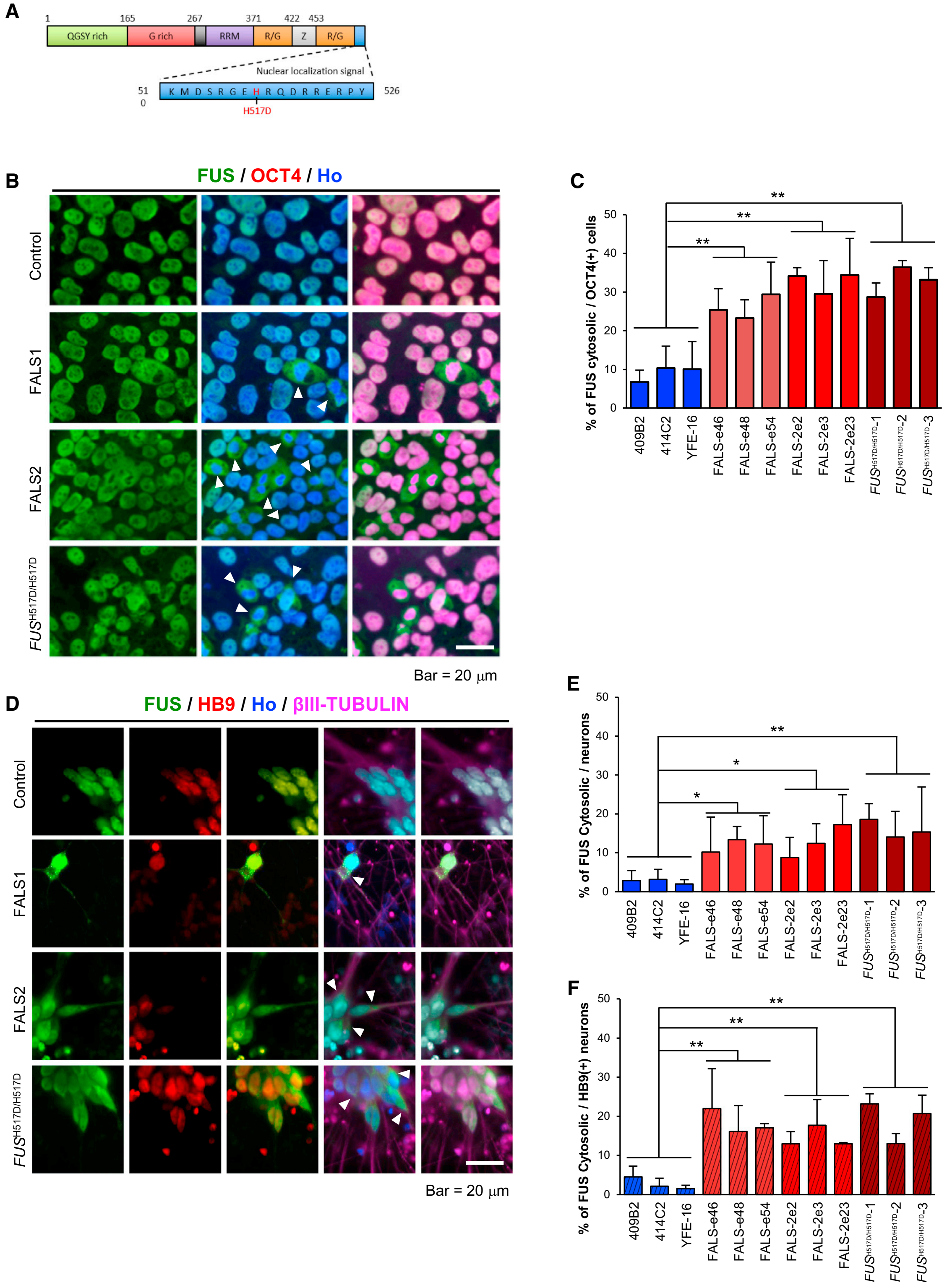

Figure 4. FUS Localization in iPSCs and iPSC-Derived Neurons

(A) Schematic diagram of FUS. The H517D mutation is located in the nuclear localization signal; RRM, RNA recognition motif; R/G, R/G rich region; Z, zinc finger domain.

(B) Representative images of immunocytochemistry for FUS in iPSCs. Arrowheads indicate cytosolic FUS. The scale bar represents 20 μm.

(legend continued on next page)

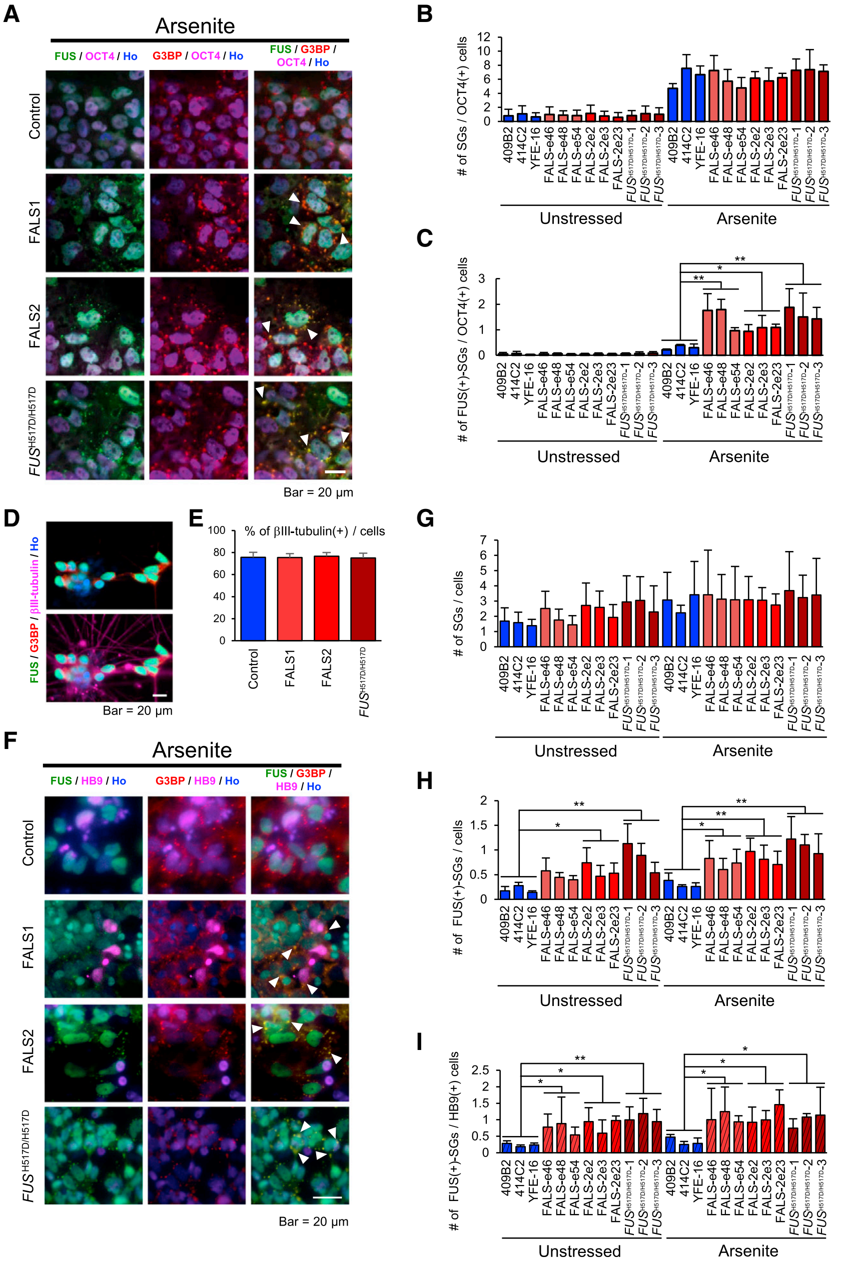

Figure 5. FUS Protein Localization into Stress Granules

(A) Representative images of immunocytochemistry for SG in iPSCs under 0.5 mM sodium arsenite stress conditions. FUS in FALS and *FUS*$^{H517D/H517D}$ iPSCs co-localized with the SG marker G3BP (arrowhead), whereas FUS in control iPSCs remained in the nucleus. The scale bar represents 20 μm. In (A)–(I) images or graphs, Arsenite means addition of 0.5 mM sodium arsenite, 1 hr treatment.

(B) Quantitative data of the number of SGs per OCT4-positive cell in iPSCs (n = 3 independent experiments; mean ± SD; Dunnett's test).

(C) Quantitative data of the number of FUS-positive SGs per OCT4-positive cell in iPSCs (n = 3 independent experiments; mean ± SD; *p < 0.05, **p < 0.01; Dunnett's test).

(D) Representative images of immunocytochemistry for βIII-TUBULIN-positive neurons. The scale bar represents 20 μm.

(E) Quantitative data of the percentage of βIII-TUBULIN-positive neurons (n = 3 independent experiments; mean ± SD; Dunnett's test).

(F) Representative images of immunocytochemistry for SG in iPSC-derived neurons under 0.5 mM sodium arsenite stress conditions. FUS co-localized with the SG marker G3BP (arrowhead). The scale bar represents 20 μm.

(G) Quantitative data of the number of SGs per Hoechst-positive cell in iPSC-derived neurons (n = 3 independent experiments; mean ± SD; Dunnett's test).

(H) Quantitative data of the number of FUS-positive SGs per Hoechst-positive cell in iPSC-derived neurons (n = 3 independent experiments; mean ± SD; *p < 0.05, **p < 0.01; Dunnett's test).

(I) Quantitative data of the number of FUS-positive SGs per HB9-positive cell in iPSC-derived neurons (n = 3 independent experiments; mean ± SD; *p < 0.05, **p < 0.01; Dunnett's test).

FUS binds to DNA as well as to RNA and regulates the expression of many transcripts in multiple steps of the gene regulation process (Dormann and Haass, 2013). In this study, we used FALS MPCs efficiently oriented into neuronal lineages including HB9-positive motor neurons to observe altered gene regulation in FALS-derived cells

(C) Quantitative data of the percentages of cytosolic FUS ratio per OCT4-positive cells in iPSCs (n = 3 independent experiments; mean ± SD; **p < 0.01; Dunnett's test).
(D) Representative images of immunocytochemistry for FUS in iPSC-derived neurons. Arrowheads indicate cytosolic FUS. The scale bar represents 20 μm.
(E) Quantitative data of the percentage of cytosolic FUS ratio per Hoechst-positive cell in iPSC-derived neurons (n = 3 independent experiments; mean ± SD; *p < 0.05, **p < 0.01; Dunnett's test).
(F) Quantitative data of the percentage of cytosolic FUS ratio per HB9-positive cell in iPSC-derived neurons (n = 3 independent experiments; mean ± SD; **p < 0.01; Dunnett's test).

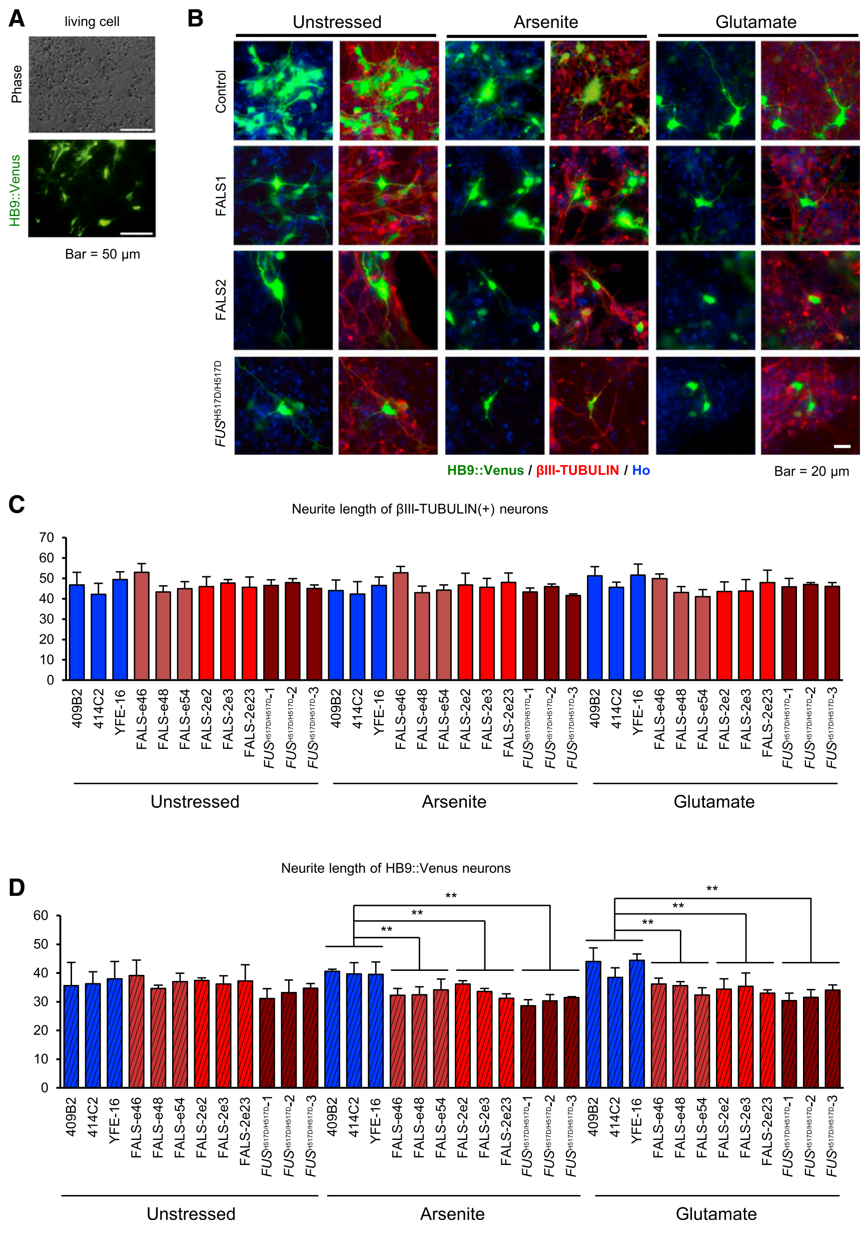

Figure 6. Shorter Neurites in FALS iPSC-Derived Motor Neurons

(A) Representative images of HB9::Venus-positive living motor neurons. The scale bars represent 50 μm.

(B) Representative images of immunocytochemistry for HB9::Venus-positive motor neurons with anti-GFP antibody. The scale bar represents 20 μm. In (B)–(D) images or graphs, Arsenite means 1.0 mM sodium arsenite, 1 hr treatment; Glutamate means 1.0 mM glutamate, 24 hr treatment.

(C) Quantitative data of the neurite length of βIII-TUBULIN-positive neurons (n = 3 independent experiments; mean ± SD; Dunnett's test).

(D) Quantitative data of the neurite length of GFP-positive motor neurons (n = 3 independent experiments; mean ± SD; **p < 0.01; Dunnett's test).

and to discover an early disease-related diagnostic marker. FUS has been shown to associate with RNA polymerase II and TFIID, thereby participating in the general transcriptional regulation process (Bertolotti et al., 1996). In addition, FUS binds to TBP and TFIID to repress transcription by RNAPIII, suggesting that FUS controls the cross-regulation between RNA polymerases (Tan and Manley, 2010). Recent studies, however, have shown that the recruitment of FUS proteins to promoter regions with lncRNA represses transcription (Wang et al., 2008). Furthermore, FUS binding to the antisense RNA transcribed by RNAPIII from promoter regions downregulates transcription (Ishigaki et al., 2012). These past studies suggest that FUS regulates transcriptional repression by various mechanisms in specific target genes. This finding could reflect our observations from our microarray assay that 78% (124 out of 159 genes) of the significantly changed genes in transcript levels are upregulated in FALS MPCs. Furthermore, with more specific analysis that focused on FUS direct targets that we defined by using the CLIP-seq dataset, we found that 95.6% of genes (of 23 genes) are upregulated in FALS.

In one proteomics study, FUS/TLS proteins were also identified as general splicing factors, which may be an early stage of the splicing process (Hartmuth et al., 2002). Our exon array analysis also revealed aberrant

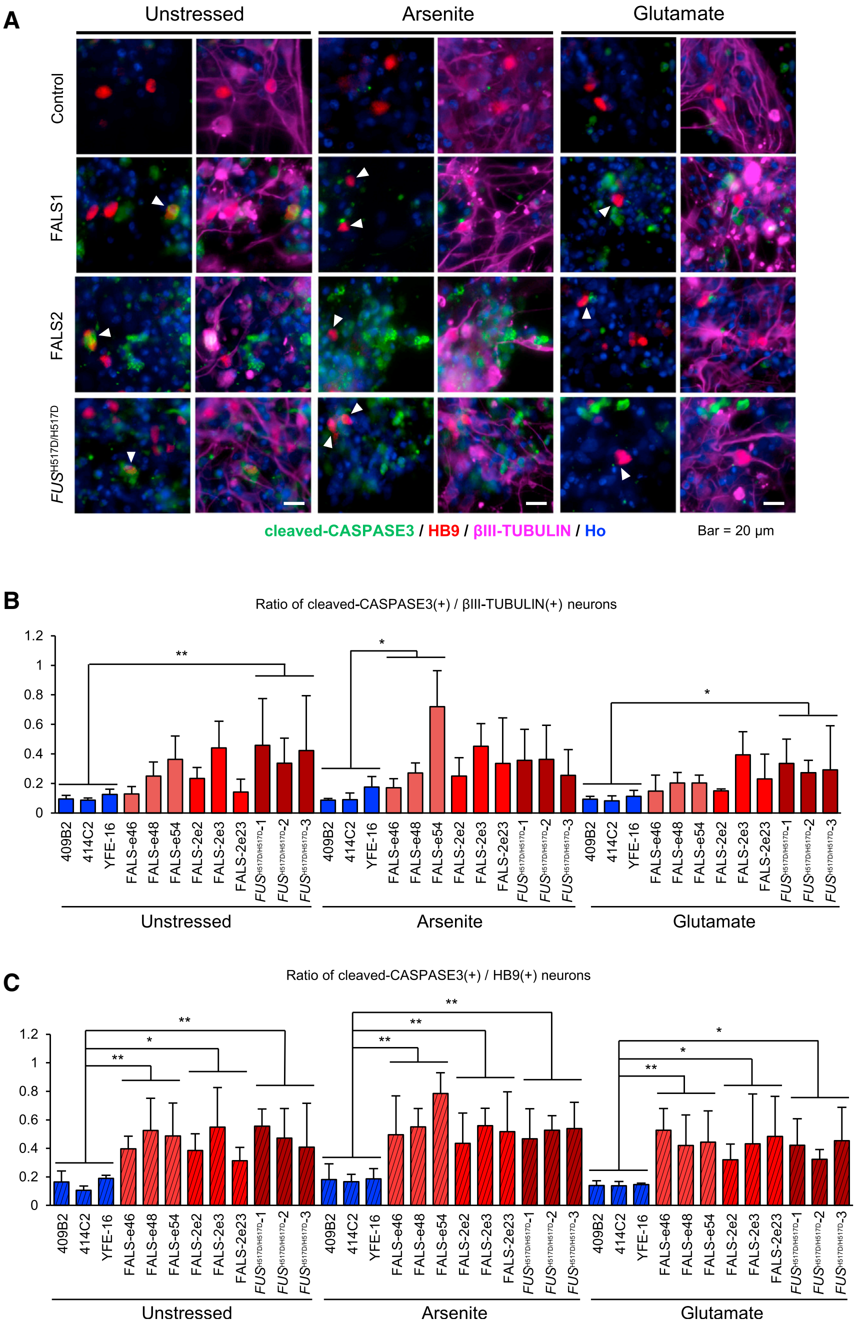

Figure 7. Enhanced Apoptosis in FALS iPSC-Derived Motor Neurons

(A) Representative images of immunocytochemistry for apoptotic HB9-positive motor neurons using markers for apoptosis (cleaved-CASPASE3), immature neurons (βIII-TUBULIN), and motor neurons (HB9). Arrowheads indicate HB9 positive cells. The scale bars represent 20 μm. In (A)–(C) images or graphs, Arsenite means 0.5 mM sodium arsenite, 1 hr treatment; Glutamate means 3.0 mM glutamate, 24 hr treatment.

(B) Quantitative data of the ratio of cleaved-CASPASE3-positive cells in βIII-TUBULIN-positive neurons (n = 3 independent experiments; mean ± SD; $^*p < 0.05$, $^{**}p < 0.01$; Dunnett's test).

(C) Quantitative data of the ratio of cleaved-CASPASE3-positive cells in HB9-positive motor neurons (n = 3 independent experiments; mean ± SD; $^*p < 0.05$, $^{**}p < 0.01$; Dunnett's test).

gene splicing events in *RSU1*, *RPH3AL*, and *EFCAB13* genes in the FALS patients. Of these, aberrant RNA processing of *RPH3AL* was also confirmed by our isogenic $FUS^{H517D/H517D}$ lines, suggesting the direct effects of FUS H517D mutation. However, in the other two alternative exons, we did not detect significant differences between control and isogenic $FUS^{H517D/H517D}$ lines, suggesting that FUS H517D mutation is not likely to have direct effects on their splicing regulation. RPH3AL is an associated protein of RAB3A (Haynes et al., 2001) and RAB27A (Fukuda, 2003) and regulates exocytosis in dense-core granules from endocrine cells (Haynes et al., 2001). In addition, mutant RPH3AL is mis-localized throughout the cytosol, whereas WT-RPH3AL is localized in the distal portion of the neurites (Fukuda et al., 2004). To date, there has been no reports of functional analyses of products from each alternative splicing event in these three genes. This may be involved in the pathological features of FUS-mediated pathologies and may also be useful as early diagnostic markers for ALS. Our iPSC model for FALS may thus

represent a useful tool for observing gene expression levels in the motor neuron lineage, as gene expression analysis in motor progenitor cells oriented into HB9-positive motor neurons cannot be achieved using conventional disease models.

We also addressed whether pathological features observed in other ALS models are observed in our in vitro FALS model. Motor neurons in patients with sporadic ALS express abundant unedited GLUA2 forms in the AMPA receptor subunit (Kawahara et al., 2004; Kwak et al., 2010), but the unedited GLUA2 form in FALS MPCs and neurons derived from iPSCs was not detected (Figure S6). ER stress is increasingly recognized as an important pathway leading to cell death in animal and cellular disease models based on mutant SOD1 (Atkin et al., 2006; Saxena et al., 2009), and mutant FUS proteins would also be predicted to induce ER stress and to interact with protein disulfide isomerase, similar to mutant SOD1 (Farg et al., 2012). In this study, however, the expression of ER stress-related genes (*BIP*, *CHOP*, spliced *XBP1*, *CASP4*, and *ASK1*) almost did not change in FALS and $FUS^{H517D/H517D}$ MPCs and neurons (Figure S7).

Our study showed that neurite length and the ratio of apoptotic cells in FALS- and $FUS^{H517D/H517D}$ iPSC-derived neurons were the same as in control motor neurons. With several stresses especially in glutamate treatment, however, FALS- and $FUS^{H517D/H517D}$ HB9-positive motor neurons exhibited shorter neurites and an increased cleaved-CASPASE3-positive cell ratio compared with control HB9-positive motor neurons. These results suggest that FALS iPSC-derived motor neurons are vulnerable to oxidative and excitatory stresses and there is a possibility that these phenomena are relevant to ALS-like phenotypes. We thus determined that these ALS patient-specific iPSC-derived motor neurons recapitulate disease phenotypes.

Recently, other groups also established in vitro FUS iPSCs models showing the inclusion of the stress granule of FUS mutant proteins (Lenzi et al., 2015; Liu et al., 2015). In addition to these findings, we observed additional ALS-like phenotypes and motor neuron vulnerability under some stress conditions and discovered potential biologically relevant aberrant gene expressions. Importantly, these events are also confirmed by the use of genome editing technology to generate isogenic mutant FUS H517D, suggesting that the present in vitro FALS model is able to recapitulate the ALS-like phenotypes. Based on these results, our mutant FUS-associated iPSC-derived HB9-positive motor neurons can be added to the list of model systems that may provide general tools for use in the analysis of the pathogenic process and drug screening studies in human motor neuron disorders.

EXPERIMENTAL PROCEDURES

Isolation of Human Skin Fibroblasts and Generation of iPSCs

The 409B2 and 414C2 iPSCs were kindly provided by Dr. Shinya Yamanaka (Okita et al., 2011). A skin-punch biopsy from a healthy 24-year-old Japanese man obtained after written informed consent (Keio University School of Medicine) was used to generate the control YFE-16 (Shimojo et al., 2015) iPSCs. FALS-1 and FALS-2 iPSCs were generated from a 39-year-old Japanese man and 43-year-old Japanese man, respectively. All the human iPSC clones were established by episomal vector transduction of transcription factors (SOX2, OCT4, KLF4, L-MYC, LIN28 and short hairpin RNA of p53) into human dermal fibroblasts (Okita et al., 2011). The cells were evaluated as described previously (Egawa et al., 2012; Müller et al., 2011; Okita et al., 2011). All the experimental procedures for skin biopsy and iPSC production were approved by the Keio University School of Medicine Ethics committee (approval number, 20080016) and Tohoku University School of Medicine Ethics committee (approval number, 2010-306).

Motor Neuron Differentiation

Motor neuron differentiation of iPSCs was performed as previously described with slight modifications (Imaizumi et al., 2015). Briefly, iPSCs were exposed to a medium including 3 μM dorsomorphin (Sigma), 3 μM SB431542 (Tocris Bioscience), and 3 μM CHIR99021 (Stemgent) over 5 consecutive days. Next, iPSCs were detached from feeder layers and then enzymatically dissociated into single cells. The dissociated cells were cultured in suspension in media hormone mix (MHM) (Okada et al., 2008) including 2% B27 supplement (Life Technologies), 2 ng/ml basic fibroblast growth factor (PeproTech), 3 μM CHIR99021 (Stemgent), 2 μM SB431542 (Tocris Bioscience), 10 μM Y27632 (Wako), 1 μM retinoic acid (Sigma), 1 μM purmorphamine (Calbiochem), and 10 ng/ml recombinant human leukemia inhibitory factor (Millipore) for 5–24 days to allow the formation of MPCs. MPCs were passaged repeatedly by dissociation into single cells followed by culture in the same manner. Typically, MPCs at three passages were used for analysis. The starting cell density was 1×10^4 cells/ml in primary MPC culture and 1×10^5 cells/ml in secondary and tertiary MPC cultures. For terminal differentiation, dissociated MPCs were allowed to adhere to poly-L-ornithine- and laminin-coated dishes with 5×10^4 cells/well in 96-well plate in MHM/B27 including 1× N2 supplement (Gibco), 1× GlutaMAX (Gibco), 10 ng/ml recombinant human brain-derived neurotrophic factor (R&D Systems), 10 ng/ml recombinant human glial cell line-derived neurotrophic factor (R&D Systems), 50 ng/ml recombinant hSHH (R&D Systems), 10 ng/ml insulin growth factor 1 (R&D Systems), 200 ng/ml ascorbic acid, 50 nM retinoic acid (Sigma), and 1 μM dibutyryl cyclic AMP (Sigma), and cultured for 20 days. Glutamate was added in the last 24 hr and sodium arsenite was added in the last 1 hr.

Immunocytochemistry

Immunocytochemistry was performed as described previously (Imaizumi et al., 2015). Detailed conditions are given in the Supplemental Information (Table S4). Fluorescence images were

acquired on AxioVision (Zeiss), BZ-9000 (Keyence), or IN Cell Analyzer (GE Healthcare).

High-Content Analysis

For the MPC population assay, neuronal subtype assay, neurite length analysis and cleaved-CASPASE3 analysis, stained plates were imaged on the high-content cellular analysis system IN Cell Analyzer 6000 (GE Healthcare) and a set of 5 × 5 fields were collected from each well using the 20× objective, resulting in over 10,000 cells being scored per well. For FUS mis-localization analysis and stress granule analysis, stained plates were imaged on IN Cell Analyzer 6000 and a set of 6 × 6 fields were collected from each well using the 60× objective, resulting in over 9,000 cells being scored per well. Analysis (IN Cell Developer Toolbox v1.9; GE Healthcare) began by identifying intact nuclei stained by Hoechst, which were defined as traced nuclei that were larger than 50 μm^2 in surface area and with intensity levels that were typical and lower than the threshold brightness of pyknotic cells. Each traced nucleus region was then expanded by 50% and cross-referenced with MPC markers (OLIG2, SOX2 and ISLET1), motor neuron markers (ISLET1, SMI32 and HB9), glutamatergic neuron marker (VGLUT1), glutamate-responsive neuron marker (GLUR1), neuron markers (MAP2 and βIII-TUBULIN), and pluripotent marker (OCT4) to identify them; from these images, the percentages of these were calculated. By setting areas on each cell type or neural subtype, the ratio of FUS mis-localization into cytosol, the number of stress granules, or the number of FUS-positive stress granules in OCT4-positve iPSCs, βIII-TUBULIN-positive neurons or HB9-positive motor neurons were analyzed. Using the above-described traced images of each cell, neurite length and the cleaved-CASPASE3-positive cell ratio in βIII-TUBULIN-positive neurons or HB9-positive motor neurons were analyzed.

Quantitative RT-PCR

RNA was isolated using an RNeasy kit (Qiagen) and reverse transcription using iScript cDNA Synthesis Kit (Bio-Rad). Quantitative RT-PCR was performed using SYBR Premix Ex Taq II (TaKaRa) on the ViiA 7 Real-Time PCR System (Life Technologies) (Table S5).

Sequence Analysis

Genomic DNA was isolated using a DNeasy kit (Qiagen) and amplified using intronic primers and direct nucleotide sequencing (Table S5). Both sense and antisense strands of all amplicons were sequenced using the Big Dye 3.1 dideoxy terminator methods (Applied Biosystems) and ABI Prism 3130xL Genetic Analyzer (Applied Biosystems).

Exon Array for MPCs

Exon array analysis was performed using an Affymetrix GeneChip Human Exon 1.0 ST Array. Data were analyzed using the GeneSpring GX7.3.1 software (Agilent), UCSC Genome browser (http://genome.ucsc.edu/index.html), and DAVID Bioinformatics Resources (http://david.abcc.ncifcrf.gov/). Exon array data have been registered in the Gene Expression Omnibus under accession number GEO: GSE76698.

SUPPLEMENTAL INFORMATION

Supplemental Information includes Supplemental Experimental Procedures, seven figures, and five tables and can be found with this article online at http://dx.doi.org/10.1016/j.stemcr.2016.02.011.

AUTHOR CONTRIBUTIONS

N.I., K.F., M.Y., and H.O. conceived and designed the experiments and wrote the manuscript. N.I. and K.F. performed most of the experiments and analyzed the data. N.I., C.I.-F., T.S., T.A., Y.O., W.A., T.M., M.I., Y.I., T.S., and T.Y. contributed to generate the patient-derived hiPSCs, isogenic hiPSCs, and analyzed the culture assay results. N.I., Y.N., H.T., and M.Y. analyzed and validated the microarray data and helped with in vitro analysis. N.S., H.W., and M.A. contributed to clinical and genetic analyses of the patient-coordinated study. All the authors read and approved the final version of the manuscript.

ACKNOWLEDGMENTS

We thank the members of the Okano laboratory for helpful comments and discussion. The research described in this study was supported by grants from the Program for Intractable Disease Research Utilizing Disease-specific iPS Cells funded by the Japan Science and Technology Agency (JST)/Japan Agency for Medical Research and Development (A-MED) to H.O., Ministry of Health, Labor and Welfare (MHLW) of Japan to H.O., Translational Research Network Program from A-MED to Keio University, the New Energy and Industrial Technology Development Organization (NEDO), and the Ministry of Education, Science, Sports and Culture (MEXT) (Scientific Research on Innovative Area, a MEXT Grant-in-Aid Project FY2014-2018 "Brain Protein Aging and Dementia Control"). H.O. is a scientific consultant for San Bio Co., Ltd.

Received: May 20, 2015
Revised: February 17, 2016
Accepted: February 18, 2016
Published: March 17, 2016

REFERENCES

Akiyama, T., Warita, H., Kato, M., Nishiyama, A., Izumi, R., Ikeda, C., Kamada, M., Suzuki, N., and Aoki, M. (2016). Genotype-phenotype relationships in familial ALS with FUS/TLS mutations in Japan. Muscle Nerve. http://dx.doi.org/10.1002/mus.25061.

Almeida, S., Gascon, E., Tran, H., Chou, H.J., Gendron, T.F., Degroot, S., Tapper, A.R., Sellier, C., Charlet-Berguerand, N., Karydas, A., et al. (2013). Modeling key pathological features of frontotemporal dementia with C9ORF72 repeat expansion in iPSC-derived human neurons. Acta Neuropathol. *126*, 385–399.

Aruga, J., and Mikoshiba, K. (2003). Identification and characterization of Slitrk, a novel neuronal transmembrane protein family controlling neurite outgrowth. Mol. Cell. Neurosci. *24*, 117–129.

Atkin, J.D., Farg, M.A., Turner, B.J., Tomas, D., Lysaght, J.A., Nunan, J., Rembach, A., Nagley, P., Beart, P.M., Cheema, S.S., et al. (2006). Induction of the unfolded protein response in familial amyotrophic

lateral sclerosis and association of protein-disulfide isomerase with superoxide dismutase 1. J. Biol. Chem. *281*, 30152–30165.

Aulas, A., Stabile, S., and Vande Velde, C. (2012). Endogenous TDP-43, but not FUS, contributes to stress granule assembly via G3BP. Mol. Neurodegener. *7*, 54.

Bentmann, E., Neumann, M., Tahirovic, S., Rodde, R., Dormann, D., and Haass, C. (2012). Requirements for stress granule recruitment of fused in sarcoma (FUS) and TAR DNA-binding protein of 43 kDa (TDP-43). J. Biol. Chem. *287*, 23079–23094.

Bertolotti, A., Lutz, Y., Heard, D.J., Chambon, P., and Toral, L. (1996). hTAF(II)68, a novel RNA/ssDNA-binding protein with homology to the pro-oncoproteins TLS/FUS and EWS is associated with both TFIID and RNA polymerase II. EMBO J. *15*, 5022–5031.

Bilican, B., Serio, A., Barmada, S.J., Nishimura, A.L., Sullivan, G.J., Carrasco, M., Phatnani, H.P., Puddifoot, C.a, Story, D., Fletcher, J., et al. (2012). Mutant induced pluripotent stem cell lines recapitulate aspects of TDP-43 proteinopathies and reveal cell-specific vulnerability. Proc. Natl. Acad. Sci. USA *109*, 5803–5808.

Bruijn, L.I., Miller, T.M., and Cleveland, D.W. (2004). Unraveling the mechanisms involved in motor neuron degeneration in ALS. Annu. Rev. Neurosci. *27*, 723–749.

Chambers, S., and Fasano, C. (2009). Highly efficient neural conversion of human ES and iPS cells by dual inhibition of SMAD signaling. Nat. Biotechnol. *27*, 275–280.

Chen, S., Sayana, P., Zhang, X., and Le, W. (2013). Genetics of amyotrophic lateral sclerosis: an update. Mol. Neurodegener. *8*, 28.

Chestkov, I., and Vasilieva, E. (2014). Patient-specific induced pluripotent stem cells for SOD1-associated amyotrophic lateral sclerosis pathogenesis studies. Acta Naturae *6*, 54–60.

Cleveland, D., and Rothstein, J. (2001). From Charcot to Lou Gehrig: deciphering selective motor neuron death in ALS. Nat. Rev. Neurosci. *2*, 806–819.

Di Salvio, M., Piccinni, V., Gerbino, V., Mantoni, F., Camerini, S., Lenzi, J., Rosa, A., Chellini, L., Loreni, F., Carrì, M.T., et al. (2015). Pur-alpha functionally interacts with FUS carrying ALS-associated mutations. Cell Death Dis. *6*, e1943.

Dimos, J.T., Rodolfa, K.T., Niakan, K.K., Weisenthal, L.M., Mitsumoto, H., Chung, W., Croft, G.F., Saphier, G., Leibel, R., Goland, R., et al. (2008). Induced pluripotent stem cells generated from patients with ALS can be differentiated into motor neurons. Science *321*, 1218–1221.

Dormann, D., and Haass, C. (2013). Fused in sarcoma (FUS): an oncogene goes awry in neurodegeneration. Mol. Cell. Neurosci. *56*, 475–486.

Dormann, D., Rodde, R., Edbauer, D., Bentmann, E., Fischer, I., Hruscha, A., Than, M.E., Mackenzie, I.R.A., Capell, A., Schmid, B., et al. (2010). ALS-associated fused in sarcoma (FUS) mutations disrupt Transportin-mediated nuclear import. EMBO J. *29*, 2841–2857.

Egawa, N., Kitaoka, S., Tsukita, K., Naitoh, M., Takahashi, K., Yamamoto, T., Adachi, F., Kondo, T., Okita, K., Asaka, I., et al. (2012). Drug screening for ALS using patient-specific induced pluripotent stem cells. Sci. Transl. Med. *4*, 145ra104.

Farg, M.A., Soo, K.Y., Walker, A.K., Pham, H., Orian, J., Horne, M.K., Warraich, S.T., Williams, K.L., Blair, I.P., and Atkin, J.D. (2012). Mutant FUS induces endoplasmic reticulum stress in amyotrophic lateral sclerosis and interacts with protein disulfide-isomerase. Neurobiol. Aging *33*, 2855–2868.

Fukuda, M. (2003). Distinct Rab binding specificity of Rim1, Rim2, rabphilin, and Noc2. Identification of a critical determinant of Rab3A/Rab27A recognition by Rim2. J. Biol. Chem. *278*, 15373–15380.

Fukuda, M., Kanno, E., and Yamamoto, A. (2004). Rabphilin and Noc2 are recruited to dense-core vesicles through specific interaction with Rab27A in PC12 cells. J. Biol. Chem. *279*, 13065–13075.

Gros-Louis, F., Gaspar, C., and Rouleau, G.A. (2006). Genetics of familial and sporadic amyotrophic lateral sclerosis. Biochim. Biophys. Acta *1762*, 956–972.

Hartmuth, K., Urlaub, H., Vornlocher, H., Will, C.L., Gentzel, M., Wilm, M., and Lührmann, R. (2002). Protein composition of human prespliceosomes isolated by a tobramycin affinity-selection method. Proc. Natl. Acad. Sci. USA *99*, 16719–16724.

Haynes, L.P., Evans, G.J., Morgan, A., and Burgoyne, R.D. (2001). A direct inhibitory role for the Rab3-specific effector, Noc2, in Ca2+-regulated exocytosis in neuroendocrine cells. J. Biol. Chem. *276*, 9726–9732.

Hester, M.E., Murtha, M.J., Song, S., Rao, M., Miranda, C.J., Meyer, K., Tian, J., Boulting, G., Schaffer, D.V., Zhu, M.X., et al. (2011). Rapid and efficient generation of functional motor neurons from human pluripotent stem cells using gene delivered transcription factor codes. Mol. Ther. *19*, 1905–1912.

Hu, B.-Y., and Zhang, S.-C. (2009). Differentiation of spinal motor neurons from pluripotent human stem cells. Nat. Protoc. *4*, 1295–1304.

Imaizumi, K., Sone, T., Ibata, K., Fujimori, K., Yuzaki, M., Akamatsu, W., and Okano, H. (2015). Controlling the regional identity of hPSC-derived neurons to uncover neuronal subtype specificity of neurological disease phenotypes. Stem Cell Rep. *5*, 1–13.

Ishigaki, S., Masuda, A., Fujioka, Y., Iguchi, Y., Katsuno, M., Shibata, A., Urano, F., Sobue, G., and Ohno, K. (2012). Position-dependent FUS-RNA interactions regulate alternative splicing events and transcriptions. Sci. Rep. *2*, 529.

Kawahara, Y., Ito, K., Sun, H., Aizawa, H., Kanazawa, I., and Kwak, S. (2004). Glutamate receptors: RNA editing and death of motor neurons. Nature *427*, 801.

Kwak, S., Hideyama, T., Yamashita, T., and Aizawa, H. (2010). AMPA receptor-mediated neuronal death in sporadic ALS. Neuropathology *30*, 182–188.

Kwiatkowski, T.J., Bosco, D.A., Leclerc, A.L., Tamrazian, E., Vanderburg, C.R., Russ, C., Davis, A., Gilchrist, J., Kasarskis, E.J., Munsat, T., et al. (2009). Mutations in the FUS/TLS gene on chromosome 16 cause familial amyotrophic lateral sclerosis. Science *323*, 1205–1208.

Lagier-Tourenne, C., Polymenidou, M., Hutt, K.R., Vu, A.Q., Baughn, M., Huelga, S.C., Clutario, K.M., Ling, S.-C., Liang, T.Y., Mazur, C., et al. (2012). Divergent roles of ALS-linked proteins FUS/TLS and TDP-43 intersect in processing long pre-mRNAs. Nat. Neurosci. *15*, 1488–1497.

Lanson, N.A., and Pandey, U.B. (2012). FUS-related proteinopathies: lessons from animal models. Brain Res. *1462*, 44–60.

Lattante, S., Rouleau, G.A., and Kabashi, E. (2013). TARDBP and FUS mutations associated with amyotrophic lateral sclerosis: summary and update. Hum. Mutat. *34*, 812–826.

Lenzi, J., De Santis, R., de Turris, V., Morlando, M., Laneve, P., Calvo, A., Caliendo, V., Chio, A., Rosa, A., and Bozzoni, I. (2015). ALS mutant FUS proteins are recruited into stress granules in induced pluripotent stem cell-derived motoneurons. Dis. Model. Mech. *8*, 755–766.

Liu, X., Chen, J., Liu, W., Li, X., Chen, Q., Liu, T., Gao, S., and Deng, M. (2015). The fused in sarcoma protein forms cytoplasmic aggregates in motor neurons derived from integration-free induced pluripotent stem cells generated from a patient with familial amyotrophic lateral sclerosis carrying the FUS-P525L mutation. Neurogenetics *16*, 223–231.

Matsumoto, T., Fujimori, K., Andoh-Noda, T., Ando, T., Kuzumaki, N., Toyoshima, M., Tada, H., Imaizumi, K., Ishikawa, M., Yamaguchi, R., et al. (2016). Functional neurons generated from T cell-derived induced pluripotent stem cells for neurological disease modeling. Stem Cell Rep. *6*, 1–14.

Mattis, V.B., and Svendsen, C.N. (2011). Induced pluripotent stem cells: a new revolution for clinical neurology? Lancet Neurol. *10*, 383–394.

Müller, F.-J., Schuldt, B.M., Williams, R., Mason, D., Altun, G., Papapetrou, E.P., Danner, S., Goldmann, J.E., Herbst, A., Schmidt, N.O., et al. (2011). A bioinformatic assay for pluripotency in human cells. Nat. Methods *8*, 315–317.

Neumann, M., Sampathu, D.M., and Kwong, L.K. (2006). Ubiquitinated TDP-43 in frontotemporal lobar degeneration and amyotrophic lateral sclerosis. Science *314*, 130–133.

Nishimoto, Y., Ito, D., Yagi, T., Nihei, Y., Tsunodo, Y., and Suzuki, N. (2010). Characterization of alternative isoforms and inclusion body of the TAR DNA-binding protein-43. J. Biol. Chem. *285*, 608–619.

Nishimoto, Y., Nakagawa, S., Hirose, T., Okano, H.J., Takao, M., Shibata, S., Suyama, S., Kuwako, K.-I., Imai, T., Murayama, S., et al. (2013). The long non-coding RNA nuclear-enriched abundant transcript 1_2 induces paraspeckle formation in the motor neuron during the early phase of amyotrophic lateral sclerosis. Mol. Brain *6*, 31.

Nizzardo, M., Simone, C., Falcone, M., Locatelli, F., Riboldi, G., Comi, G.P., and Corti, S. (2010). Human motor neuron generation from embryonic stem cells and induced pluripotent stem cells. Cell. Mol. Life Sci. *67*, 3837–3847.

Okada, Y., Matsumoto, A., Shimazaki, T., Enoki, R., Koizumi, A., Ishii, S., Itoyama, Y., Sobue, G., and Okano, H. (2008). Spatiotemporal recapitulation of central nervous system development by murine embryonic stem cell-derived neural stem/progenitor cells. Stem Cells *26*, 3086–3098.

Okano, H., and Yamanaka, S. (2014). iPS cell technologies: significance and applications to CNS regeneration and disease. Mol. Brain *7*, 22.

Okita, K., Matsumura, Y., Sato, Y., Okada, A., Morizane, A., Okamoto, S., Hong, H., Nakagawa, M., Tanabe, K., Tezuka, K., et al. (2011). A more efficient method to generate integration-free human iPS cells. Nat. Methods *8*, 409–412.

Ricketts, T., McGoldrick, P., Fratta, P., de Oliveira, H.M., Kent, R., Phatak, V., Brandner, S., Blanco, G., Greensmith, L., Acevedo-Arozena, A., et al. (2014). A nonsense mutation in mouse Tardbp affects TDP43 alternative splicing activity and causes limb-clasping and body tone defects. PLoS One *9*, e85962.

Robberecht, W., and Philips, T. (2013). The changing scene of amyotrophic lateral sclerosis. Nat. Rev. Neurosci. *14*, 248–264.

Rutherford, N.J., Zhang, Y., Baker, M., Gass, J.M., Finch, N.A., Xu, Y., Stewart, H., Kelley, B.J., Kuntz, K., Crook, R.J.P., et al. (2008). Novel mutations in TARDBP (TDP-43) in patients with familial amyotrophic lateral sclerosis. PLoS Genet. *4*, e1000193.

Sareen, D., O'Rourke, J.G., Meera, P., Muhammad, A.K., Grant, S., Simpkinson, M., Bell, S., Carmona, S., Ornelas, L., Sahabian, A., et al. (2013). Targeting RNA foci in iPSC-derived motor neurons from ALS patients with a C9ORF72 repeat expansion. Sci. Transl. Med. *5*, 208ra149.

Saxena, S., Cabuy, E., and Caroni, P. (2009). A role for motoneuron subtype-selective ER stress in disease manifestations of FALS mice. Nat. Neurosci. *12*, 627–636.

Shimojo, D., Onodera, K., Doi-torii, Y., Ishihara, Y., Hattori, C., Miwa, Y., Tanaka, S., Okada, R., Ohyama, M., Shoji, M., et al. (2015). Rapid, efficient and simple motor neuron differentiation from human pluripotent stem cells. Mol. Brain *8*, 79.

Sreedharan, J., Blair, I.P., Tripathi, V.B., Hu, X., Vance, C., Rogelj, B., Ackerley, S., Durnall, J.C., Williams, K.L., Buratti, E., et al. (2008). TDP-43 mutations in familial and sporadic amyotrophic lateral sclerosis. Science *319*, 1668–1672.

Surmacz, B., Fox, H., Gutteridge, A., Fish, P., Lubitz, S., and Whiting, P. (2012). Directing differentiation of human embryonic stem cells toward anterior neural ectoderm using small molecules. Stem Cells *30*, 1875–1884.

Suzuki, N., Kato, S., Kato, M., Warita, H., Mizuno, H., Kato, M., Shimakura, N., Akiyama, H., Kobayashi, Z., Konno, H., et al. (2012). FUS/TLS-immunoreactive neuronal and glial cell inclusions increase with disease duration in familial amyotrophic lateral sclerosis with an R521C FUS/TLS mutation. J. Neuropathol. Exp. Neurol. *71*, 779–788.

Tan, A.Y., and Manley, J.L. (2010). TLS inhibits RNA polymerase III transcription. Mol. Cell. Biol. *30*, 186–196.

Tsao, W., Jeong, Y.H., Lin, S., Ling, J., Price, D.L., Chiang, P.-M., and Wong, P.C. (2012). Rodent models of TDP-43: recent advances. Brain Res. *1462*, 26–39.

Vance, C., Rogelj, B., Hortobágyi, T., De Vos, K.J., Nishimura, A.L., Sreedharan, J., Hu, X., Smith, B., Ruddy, D., Wright, P., et al. (2009). Mutations in FUS, an RNA processing protein, cause familial amyotrophic lateral sclerosis type 6. Science *323*, 1208–1211.

Vance, C., Scotter, E.L., Nishimura, A.L., Troakes, C., Mitchell, J.C., Kathe, C., Urwin, H., Manser, C., Miller, C.C., Hortobágyi, T., et al. (2013). ALS mutant FUS disrupts nuclear localization and sequesters wild-type FUS within cytoplasmic stress granules. Hum. Mol. Genet. *22*, 2676–2688.

Wang, X., Arai, S., Song, X., Reichart, D., Du, K., Pascual, G., Tempst, P., Rosenfeld, M.G., Glass, C.K., and Kurokawa, R. (2008). Induced ncRNAs allosterically modify RNA-binding proteins in cis to inhibit transcription. Nature *454*, 126–130.

Yang, S., Warraich, S.T., Nicholson, G.A., and Blair, I.P. (2010). Fused in sarcoma/translocated in liposarcoma: a multifunctional DNA/RNA binding protein. Int. J. Biochem. Cell Biol. *42*, 1408–1411.

Zhou, Y., Liu, S., Liu, G., Oztürk, A., and Hicks, G.G. (2013). ALS-associated FUS mutations result in compromised FUS alternative splicing and autoregulation. PLoS Genet. *9*, e1003895.

Stem Cell Reports, Volume *6*

Supplemental Information

Establishment of In Vitro FUS-Associated Familial Amyotrophic Lateral Sclerosis Model Using Human Induced Pluripotent Stem Cells

Naoki Ichiyanagi, Koki Fujimori, Masato Yano, Chikako Ishihara-Fujisaki, Takefumi Sone, Tetsuya Akiyama, Yohei Okada, Wado Akamatsu, Takuya Matsumoto, Mitsuru Ishikawa, Yoshinori Nishimoto, Yasuharu Ishihara, Tetsushi Sakuma, Takashi Yamamoto, Hitomi Tsuiji, Naoki Suzuki, Hitoshi Warita, Masashi Aoki, and Hideyuki Okano

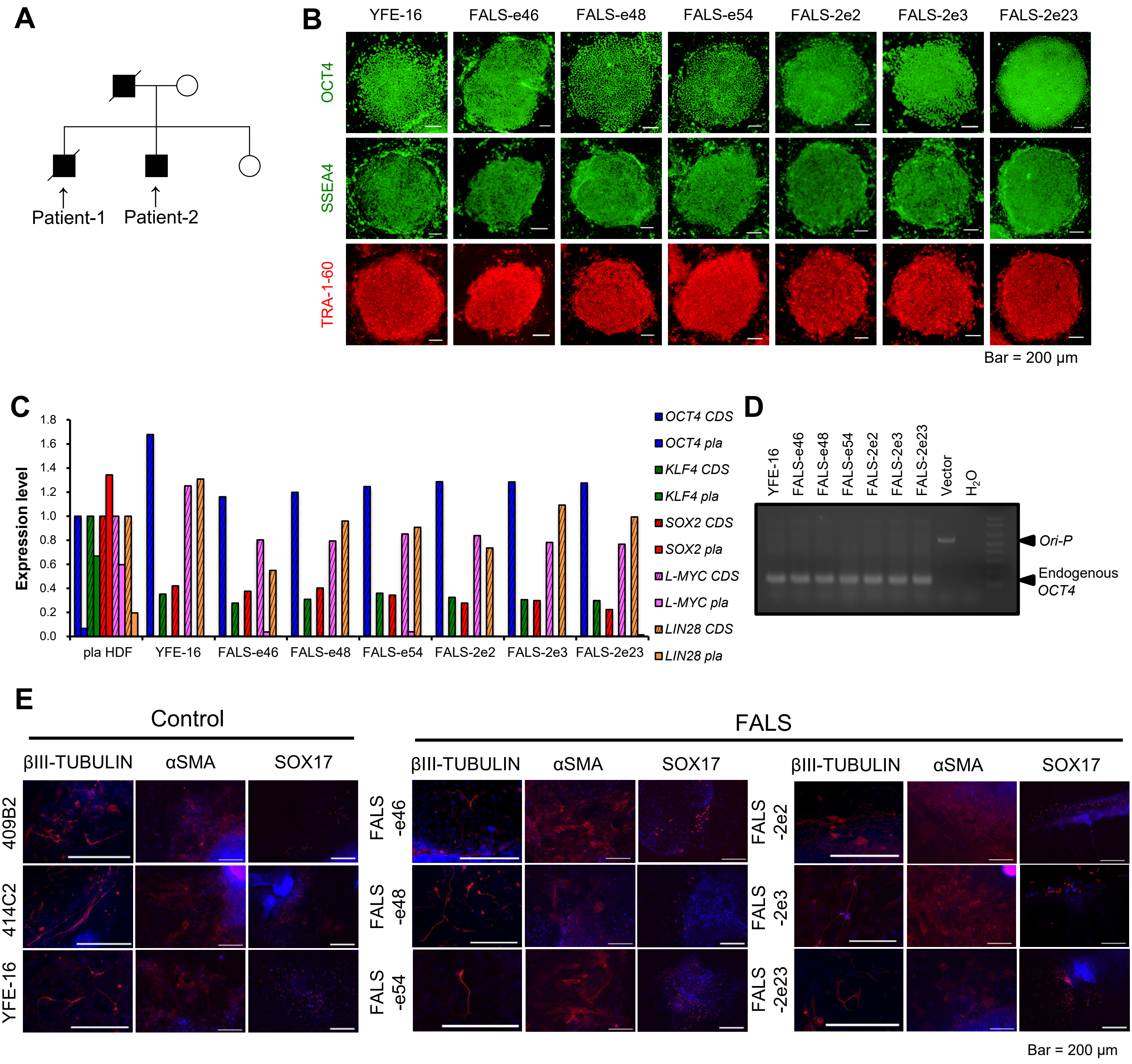

Figure S1. Pluripotent markers, Transgene expression, Genome integration, and *in vitro* differentiation into three-germ layers (Related to Figure 1)

A. Pedigree of the patients carrying *FUS*^{H517D} mutation (extracted from Akiyama et al. (2016)). iPSCs were generated from Patient-1 and Patient-2. The detailed pedigree tree of this family is described in Akiyama et al. (2016).

B. Representative image of immunochemical analysis of the pluripotent markers, OCT4, SSEA4, and TRA-1-60. The scale bars represent 200 μm.

C. Quantitative RT-PCR analysis of the expression of transgenes of episomal vectors in established YFE-16 and FALS iPSC clones. The data are presented as the mRNA copy numbers for each transgene divided by those for *ACTB*. As a control, fibroblasts at 6 days after transfection of 5 episomal vectors were analyzed (pla HDF). CDS, for detection of coding sequence; pla, for detection of plasmid vector-derived expression.

D. Integrated episomal vectors were detected by PCR for *Ori-P* cassette in established YFE-16 and FALS iPSC clones. Endogenous *OCT4* was detected for the expression control, and Vector and H2O were positive and negative controls, respectively.

E. Representative image of immunocytochemistry for the in vitro three-germ layer assay. βIII-TUBULIN, αSMA, and SOX17 are the markers of ectoderm, mesoderm, and endoderm, respectively. Scale bars, 200 μm.

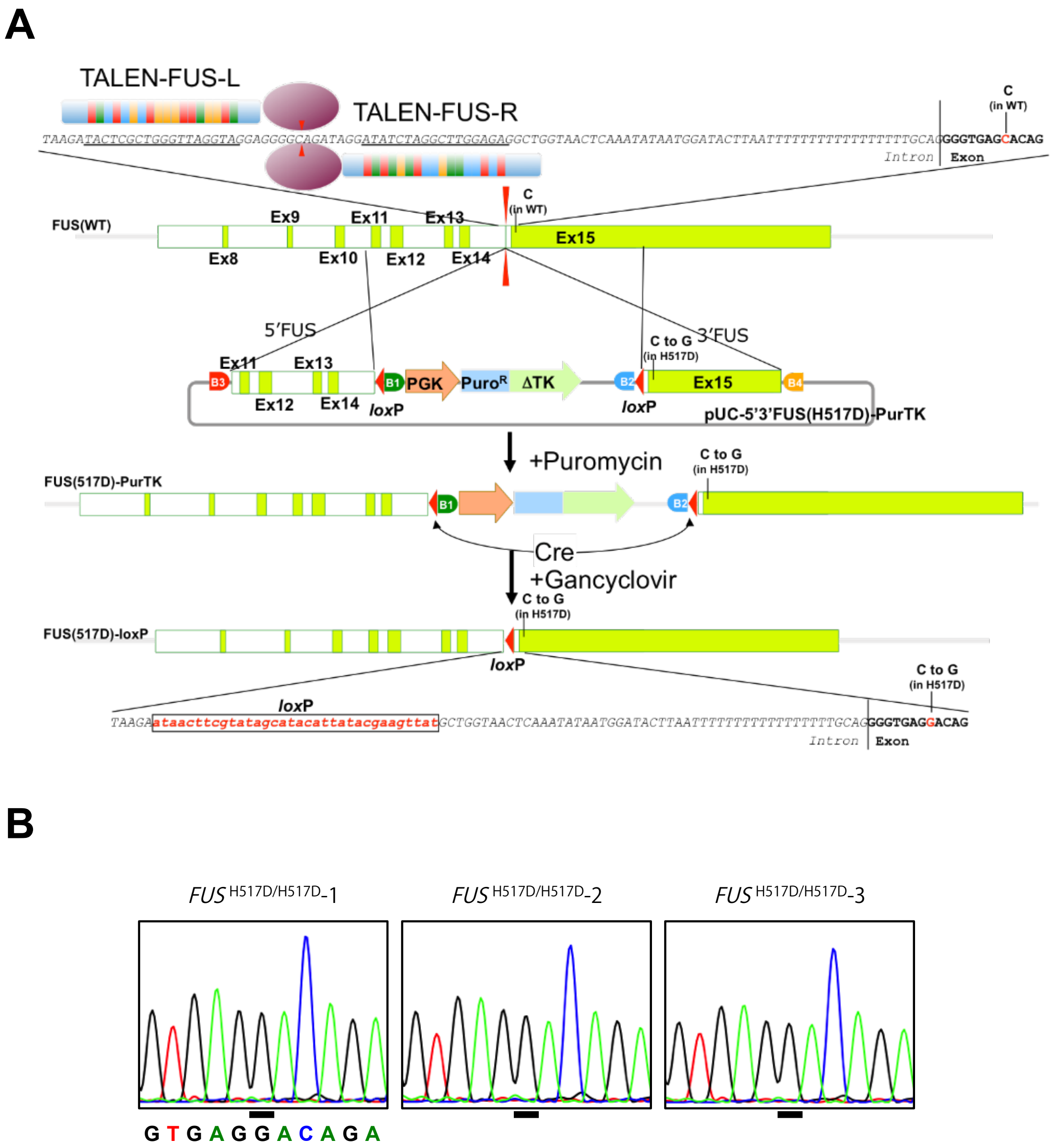

Figure S2. TALEN-mediated genome editing in iPSC (Related to Figure 1)

A. Schematic presentation of the protocols for TALEN-mediated genome editing in iPSC.

B. The *FUS* H517D (C-to-G) homozygous mutations identified in TALEN-mediated iPSCs, *FUS*$^{H517D/H517D}$-1, -2, and -3.

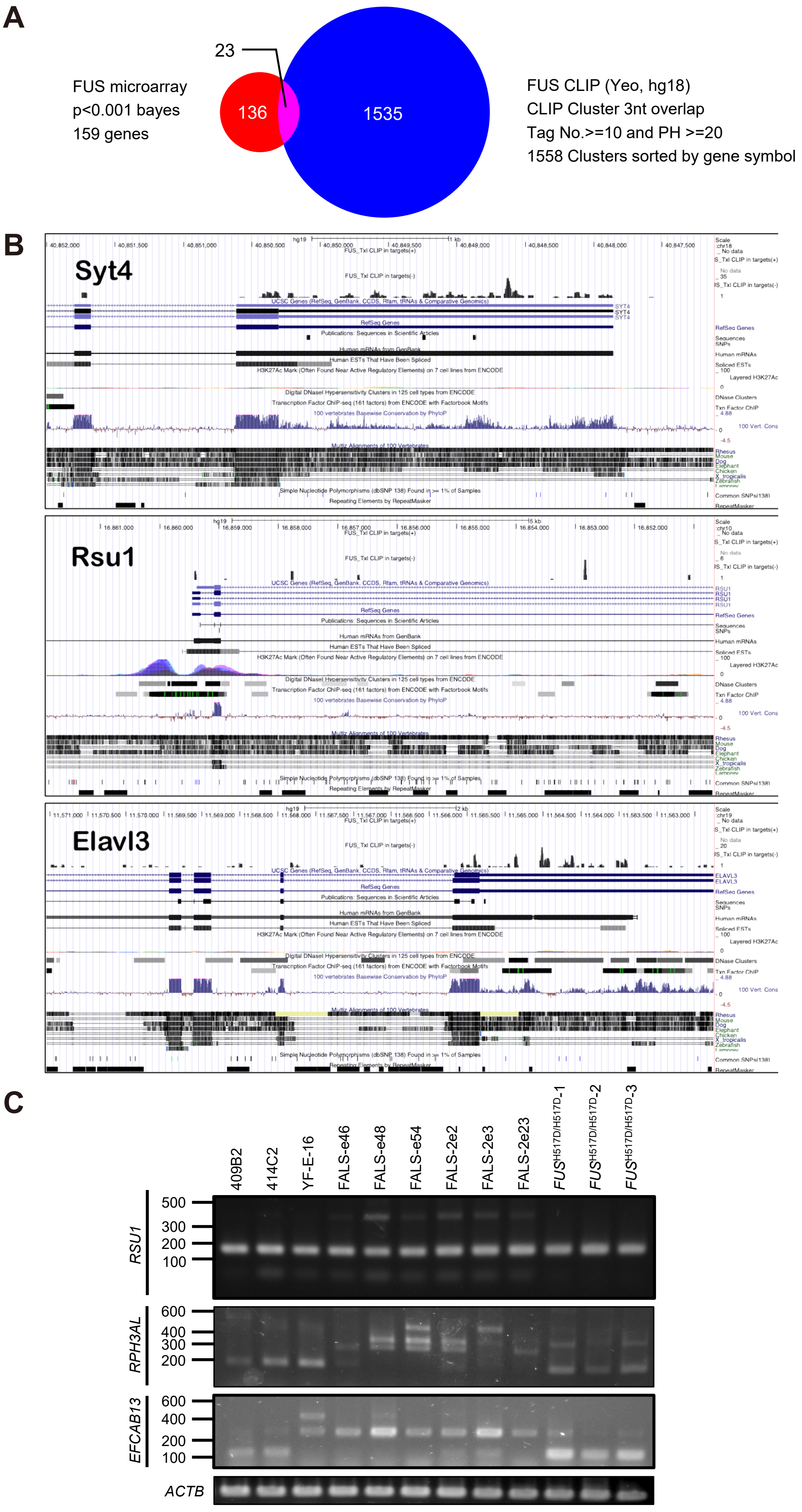

Figure S3. Comparison with exon array and CLIP-seq, and alternative splicing analysis on MPCs (Related to Figure 2 and Figure 3)

A. Venn diagram showing the number of exon array clusters (red) and FUS CLIP-seq clusters (blue) that overlap (purple) each other. Exon array clusters were then restricted within p<0.001 in Bayesian statistics, and CLIP-seq clusters were then restricted within a 3 nt overlap, tag numbers of more than 10, and peak height (PH) of more than 20.

B. The graphic view from the genome browser showing general gene information, scale, chromosomal coordinates, and the genes *SYT4*, *RSU1*, and *ELAVL3*.

C. Representative image of RT-PCR of splicing variants in *RSU1*, *RPH3AL*, and *EFCAB13* in iPSC-derived MPCs.

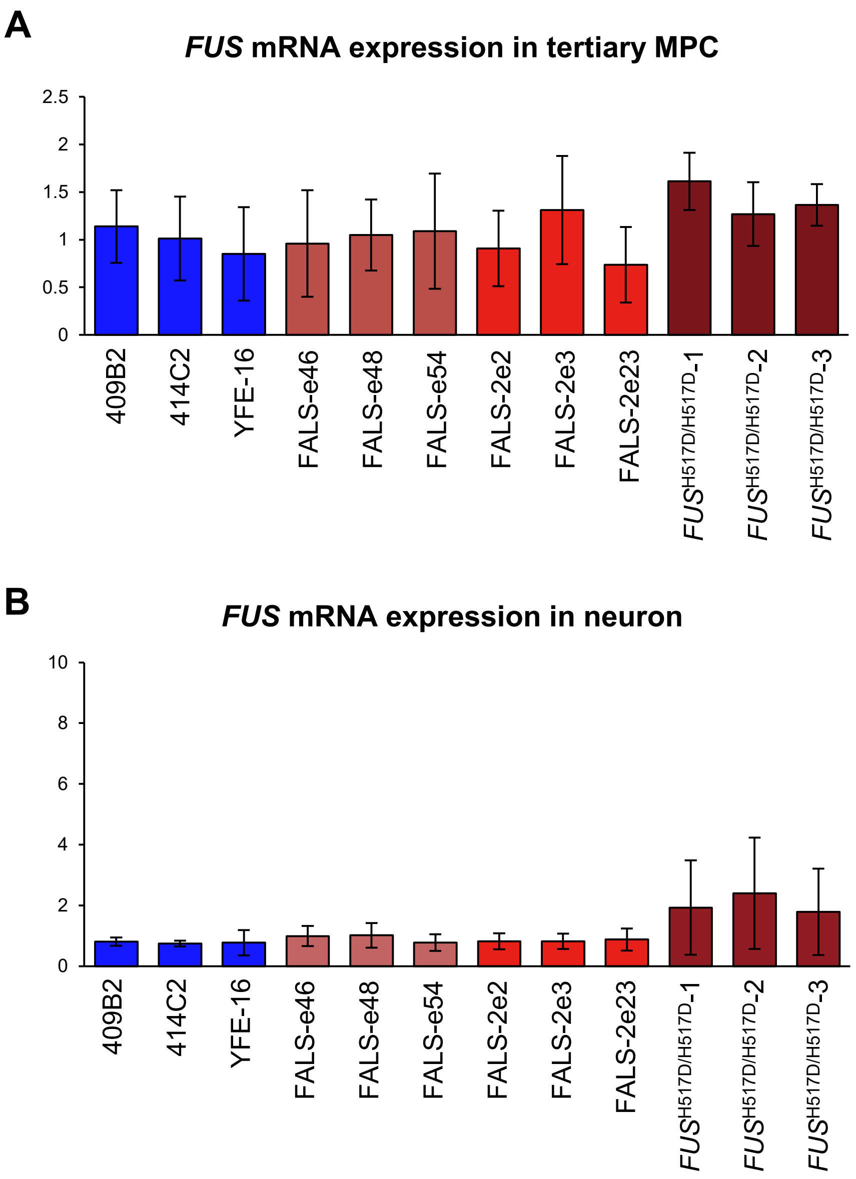

Figure S4. *FUS* gene expression (Related to Figure 4)

Quantitative RT-PCR analysis of the expression of the *FUS* gene in MPCs (A) and neurons (B). The data are presented as the mRNA copy numbers divided by *ACTB*. (n = 3 independent experiments; mean ± SD; Dunnett's test)

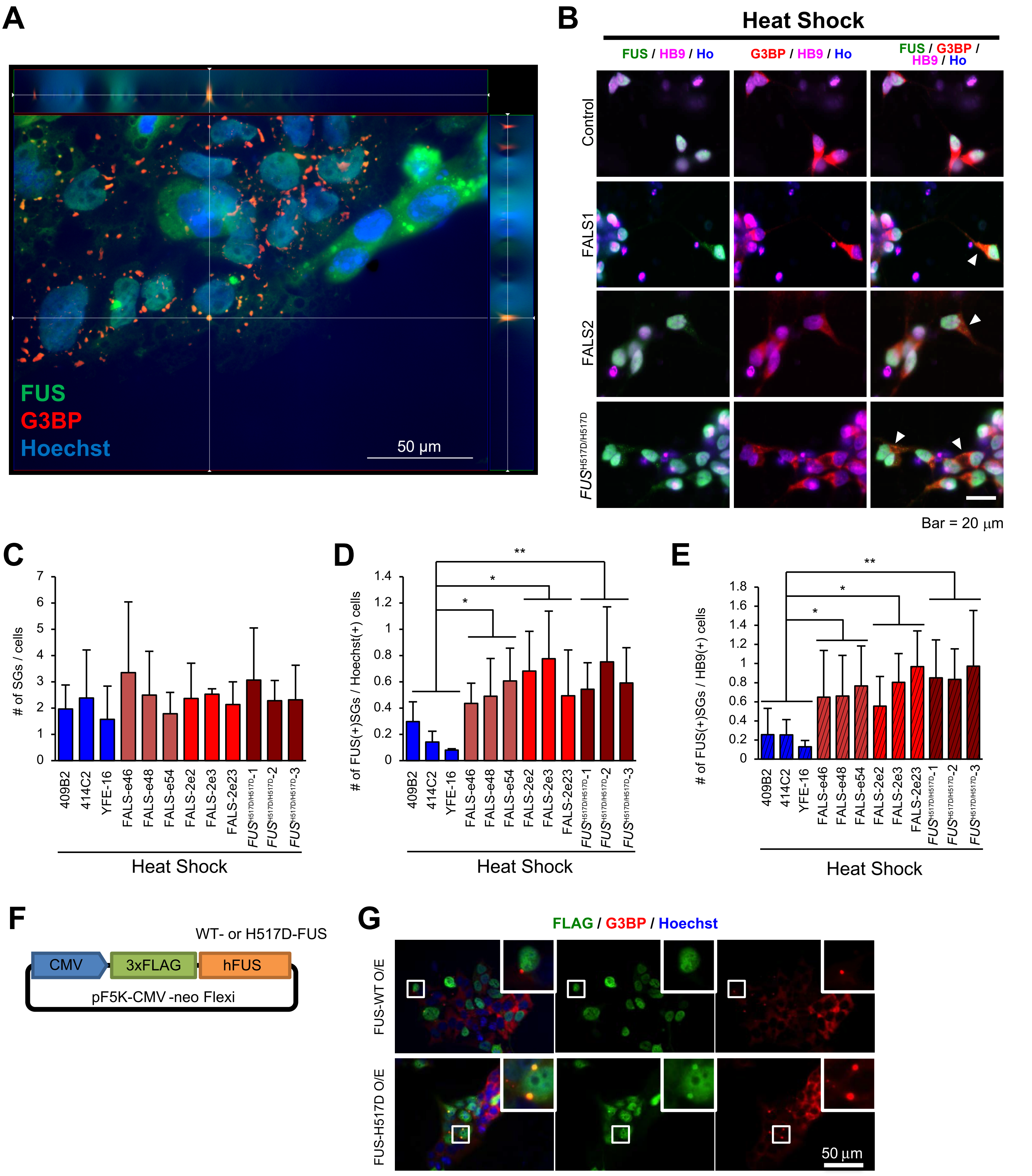

Figure S5. FUS protein localizes into stress granules under heat shock stress condition in iPSC and iPSC-derived neuron, and under arsenite treatment in FUS overexpression system (Related to Figure 5)

A. Representative orthogonal image of immunocytochemistry for FUS and SGs. FUS-positive aggregates were wrapped by G3BP-positive SGs. The scale bar represents 50 µm.

B. Representative images of immunocytochemistry for SG in iPSC-derived neurons under 44 °C heat shock condition. FUS co-localized with the SG marker G3BP (arrowhead). The scale bars represent 20 µm.

C. Quantitative data of the number of SGs per Hoechst-positive cells in iPSC-derived neurons under 44 °C condition (n = 3 independent experiments; mean ± SD; Dunnett's test).

D. Quantitative data of the number of FUS-positive SGs per Hoechst-positive cells in iPSC-derived neurons under 44 °C condition (n = 3 independent experiments; mean ± SD; Dunnett's test).

E. Quantitative data of the number of FUS-positive SG in HB9-positive motor neurons under 44 °C condition (n = 3 independent experiments; mean ± SD; Dunnett's test).

F. Schematic diagram of WT- or H517D-FUS expression vectors; CMV, CMV promoter sequence; 3xFLAG, 3xFLAG tag sequence.

G. Representative image of immunocytochemistry for SG in 293T cells transfected WT- or H517D-FUS expression vector. FLAG-stained cells expressed transfected FUS and H517D-FUS co-localized with G3BP. The scale bars represent 50 µm.

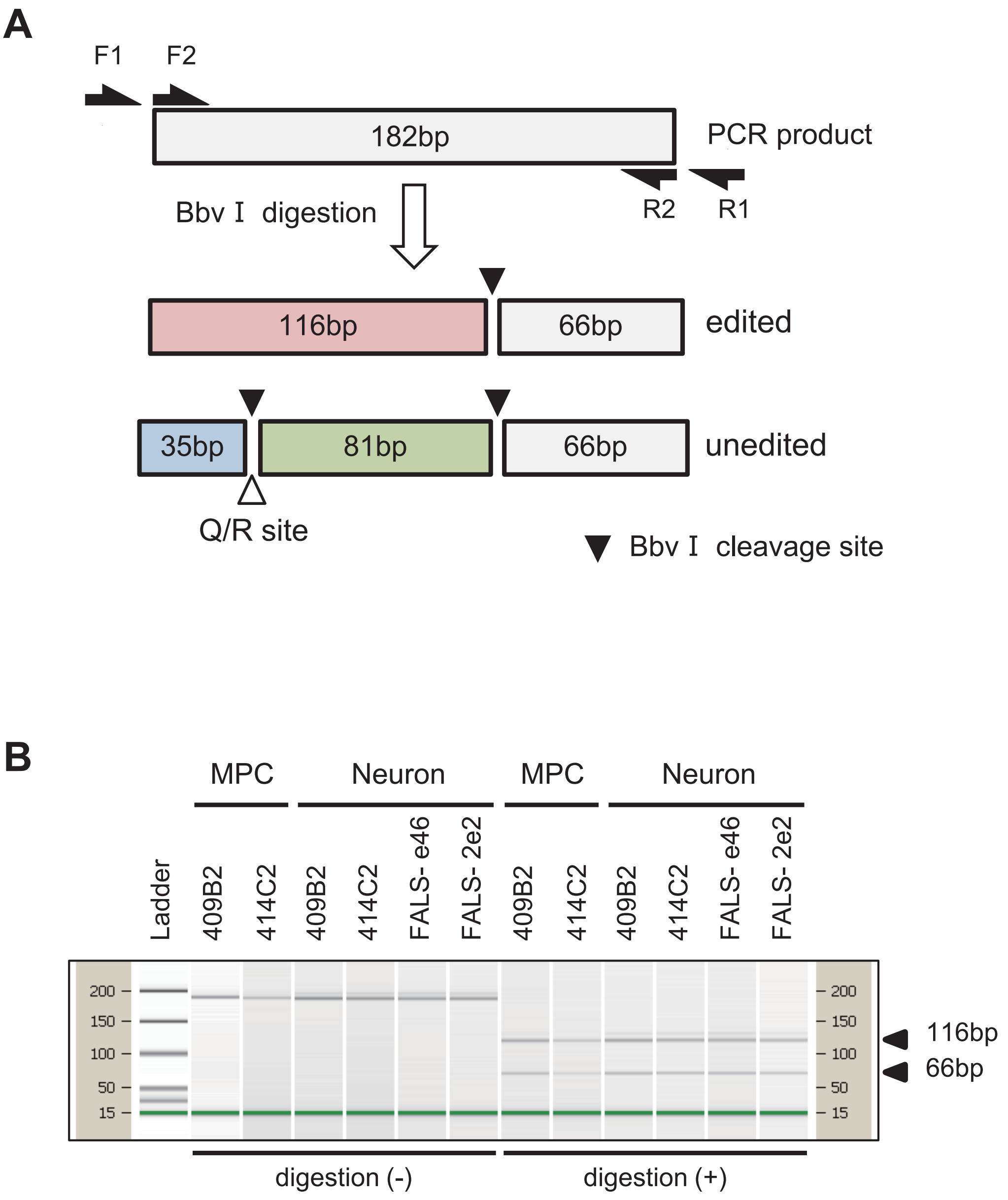

Figure S6. RNA editing analysis in AMPA receptor GluA2 subunit (Related to Figure 7)

A. Schematic diagram of the protocols for detecting the editing efficiency at the Q/R site of GluA2. Open bars represent nested PCR products. Intrinsic BbvI recognition sites are indicated by vertical solid arrowheads. The sizes of the DNA fragments generated by restriction digestion are indicated. Q, glutamine; R, arginine.

B. The gel-like image produced by the 2100 Bioanalyzer. All samples showed 116 bp and 66 bp bands with digestion.

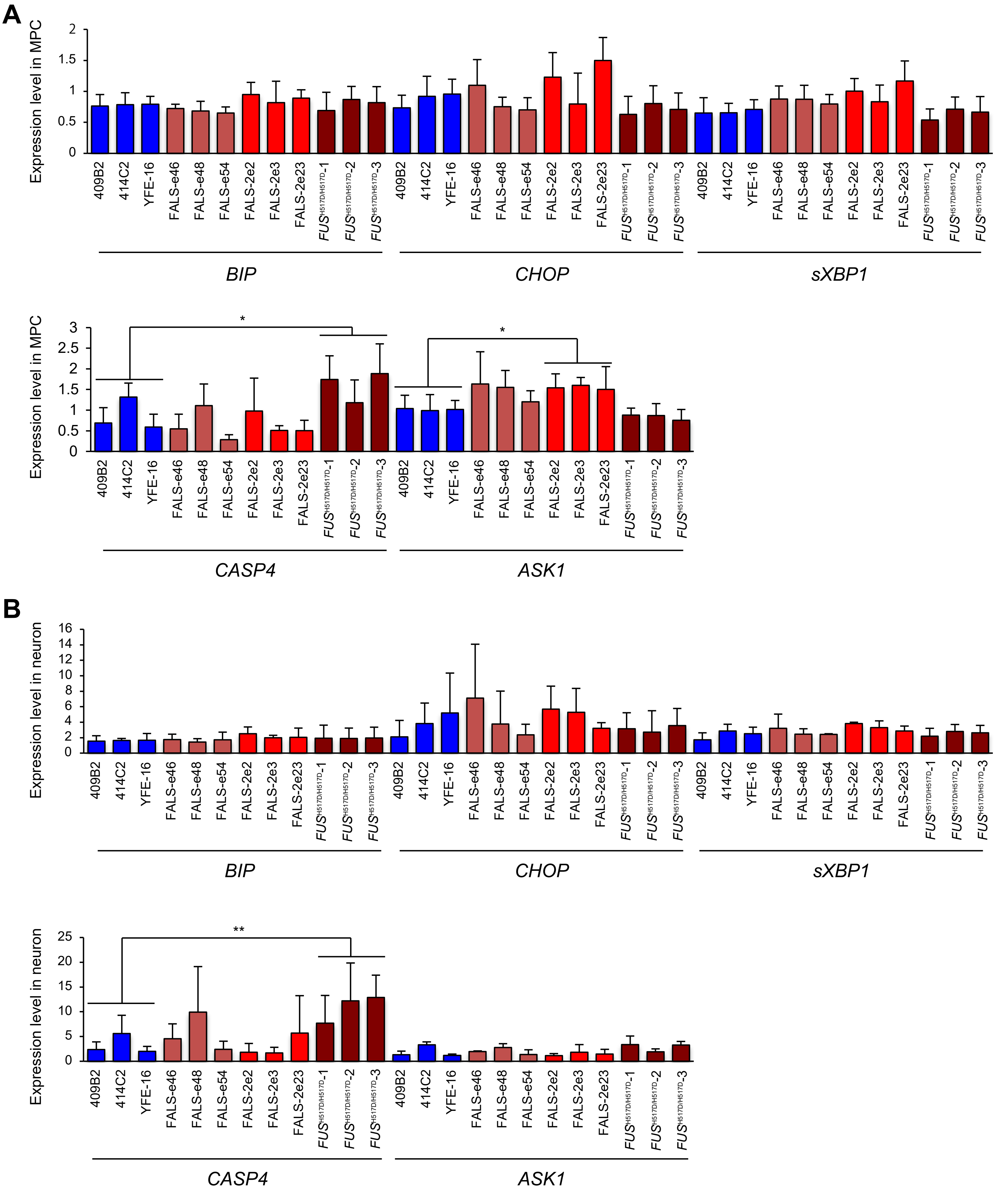

Figure S7. ER stress related gene expression (Related to Figure 7)
Quantitative RT-PCR analysis of the expression of ER stress marker genes in iPSC-derived MPCs (A) and neurons (B). The data are presented as the mRNA copy numbers for each gene divided by those for *ACTB*. (n = 3 independent experiments; mean ± SD; *p<0.05, ** p<0.01; Dunnett's test)

Table S1. Clinical information

Control or FALS	Cell line	Sex	Race	Age	Diagnosis
Control	409B2	Female	Caucasian	36	Healthy
	414C2				
	YFE-16	Male	Japanese	24	Healthy
FALS1	FALS-e46	Male	Japanese	39	Familial ALS
	FALS-e48				
	FALS-e54				
FALS2	FALS-2e2	Male	Japanese	42	Familial ALS
	FALS-2e3				
	FALS-2e23				

Table S2. Overlapping genes between Exon array and FUS CLIP-seq

FC	Stat ebayes	Gene symbol	Gene description
2.260	-5.087	NETO1	neuropilin (NRP) and tolloid (TLL)-like 1
1.980	-5.571	NFASC	neurofascin homolog (chicken)
1.965	-6.703	ST18	suppression of tumorigenicity 18 (breast carcinoma) (zinc finger protein)
1.817	-5.424	CADPS	Ca++-dependent secretion activator
1.684	-6.771	CNTNAP5	contactin associated protein-like 5
1.630	-6.717	SYT4	synaptotagmin IV
1.626	-6.190	LRP1B	low density lipoprotein receptor-related protein 1B | low density lipoprotein-related protein 1B (deleted in tumors)
1.599	-4.963	ANK2	ankyrin 2, neuronal
1.568	-5.448	PDE8B	phosphodiesterase 8B
1.522	-6.164	SNAP91	synaptosomal-associated protein, 91kDa homolog (mouse)
1.514	-5.846	FUT9	fucosyltransferase 9 (alpha (1,3) fucosyltransferase)
1.511	-6.688	ELAVL3	ELAV (embryonic lethal, abnormal vision, Drosophila)-like 3 (Hu antigen C)
1.504	-6.608	GNAO1	guanine nucleotide binding protein (G protein), alpha activating activity polypeptide O
1.436	-4.508	FMN2	formin 2
1.320	-5.692	SLC36A1	solute carrier family 36 (proton/amino acid symporter), member 1
1.313	-4.574	NAPB	N-ethylmaleimide-sensitive factor attachment protein, beta
1.300	-6.983	LRRC8B	leucine rich repeat containing 8 family, member B
1.290	-4.677	PREX1	phosphatidylinositol-3,4,5-trisphosphate-dependent Rac exchange factor 1
1.268	-6.804	SGIP1	SH3-domain GRB2-like (endophilin) interacting protein 1
1.262	-4.765	PSD3	pleckstrin and Sec7 domain containing 3
1.251	-5.125	DST	dystonin
1.206	-4.988	PPA2	pyrophosphatase (inorganic) 2
0.807	5.220	RPAIN	RPA interacting protein

Table S3. Exon array correlation graph

	409 B2	414 C2	YFE-16	FALS-e46	FALS-e48	FALS-e54	FALS-2e2	FALS-2e3	FALS-2e23
409 B2	1	0.9744	0.9761	0.9761	0.9708	0.9754	0.9727	0.9746	0.9774
414 C2	0.9744	1	0.9735	0.9723	0.9746	0.9724	0.9765	0.9782	0.9756
YFE-16	0.9761	0.9735	1	0.9748	0.9716	0.9748	0.9766	0.9803	0.9823
FALS-e46	0.9761	0.9723	0.9748	1	0.9792	0.9813	0.9776	0.9789	0.9796
FALS-e48	0.9708	0.9746	0.9716	0.9792	1	0.9775	0.9779	0.9784	0.976
FALS-e54	0.9754	0.9724	0.9748	0.9813	0.9775	1	0.976	0.978	0.9787
FALS-2e2	0.9727	0.9765	0.9766	0.9776	0.9779	0.976	1	0.9808	0.9798
FALS-2e3	0.9746	0.9782	0.9803	0.9789	0.9784	0.978	0.9808	1	0.9837
FALS-2e23	0.9774	0.9756	0.9823	0.9796	0.976	0.9787	0.9798	0.9837	1

Table S4. Antibody list

Antigen	Supplier	Cat No	Dilution ratio
Cleaved-CASPASE3	Cell Signaling Technology	9661	1:500
FUS	Bethyl Laboratories	A300-293A	1:1000
G3BP	BD Bioscience	611126	1:500
G3BP	Life Technologies	PA1-27843	1:1000
GFP	MBL	598	1:500
GLUR1	Millipore	04-855	1:500
HB9	DSHB	81.5C10	1:150
ISLET1	DSHB	39.4D5	1:1000
MAP2	Sigma	M4403	1:1000
OLIG2	R&D Systems	AF2418	1:500
SMI32	Covance	SMI-32P	1:2500
SOX2	Abcam	ab59776	1:200
SOX17	R&D Systems	AF1924	1:500
SSEA4	Abcam	ab16287	1:500
TRA-1-60	Millipore	MAB4360	1:500
VGLUT1	Synaptic Systems	135-303	1:1000
αSMA	Sigma	A2547	1:500
βIII-TUBULIN	Sigma	T8660	1:1000
βIII-TUBULIN	Covance	MMS-435P	1:2000

Table S5. Primer list

Gene	Forward	Reverse	Assay
FUS	ATGGCCTCAAACGATTATACCCA	GTAACTCTGCTGTCCGTAGGG	qPCR
FUS	GGGGCAACTTTGTATAATAAAGTTGCC GGAAATCCTATCAAGGTCTC (B3-5'FUS-Fw)	TTCGTATAATGTATGCTATACGAAGTTAT TCTTATCTCAAGTGGGTCTAC (LoxP-5'FUS-Rv)	TALEN
FUS	GGGGCAACTTTGTATAATAAAGTTGCC GGAAATCCTATCAAGGTCTC (B3-5'FUS-Fw)	GGGCTGCTTTTTTGTACAAACTTGATAA CTTCGTATAATGTATGCTATAC (B1r-LoxP-Rv)	TALEN
FUS	TTCGTATAGCATACATTATACGAAGTTAT GCTGGTAACTCAAATATAATG (PxoL-3'FUS-Fw)	GGGGCAACTTTGTATAGAAAAGTTGTC TTGGGTTAATGTTACGCTCT (B4-3'FUS-Rv)	TALEN
FUS	GGGCAGCTTTCTTGTACAAAGTGGATA ACTTCGTATAGCATACATTATAC (B2r-PxoL-Fw)	GGGGCAACTTTGTATAGAAAAGTTGTC TTGGGTTAATGTTACGCTCT (B4-3'FUS-Rv)	TALEN
FUS	GGGGCAAGTTTGTACAAAAAAGCAGA ATTCTACCGGGTAGGGGA (B1-EcoR1-pPGK-F')	GGGGCCACTTTGTACAAGAAAGCTGC TGCAGCCCTCGACTCTAG (B2-XbaI-PGKpA-R)	TALEN
FUS	GAAAGGCACGCTTCTCTTGTATTTTCG GAT (5'FUS-PCR-Fw)	TGCTGTCCATCTGCACGAGACTAGTGA G (PGKP-Rv)	PCR genotyping
FUS	CGAGCGGGTCACCGAGCTGCAAGAAC TC (PuroR-Fw)	TGGCAGCTGTCTTACAAACCAAGTTCG AAA (3'FUS-PCR-Rv)	PCR genotyping
FUS	GAAAGGCACGCTTCTCTTGTATTTTCG GAT (5'FUS-PCR-Fw)	TGGCAGCTGTCTTACAAACCAAGTTCG AAA (3'FUS-PCR-Rv)	genomic PCR
FUS	CCGGGGTGGTGGGGACAGAGGTGG (Seq-FUS-Fw)	TCATTTGGCCTTCTCCCCGAACACT (Seq-FUS-Rv)	Sequence
ASK1	CTGCATTTTGGGAAACTCGACT	AAGGTGGTAAAACAAGGACGG	qPCR
SLITRK4	CCTGATTTCTTCGACAAATGCAG	TCTCACAGTTGACATAGAGCACA	qPCR
ALCAM	TCCTGCCGTCTGCTCTTCT	TTCTGAGGTACGTCAAGTCGG	qPCR
NEUROD4	ACCAGGTACTTATGGGATGCT	AAGGCGAGCTTTGGTCATCTT	qPCR
ONECUT2	GGAATCCAAAACCGTGGAGTAA	CTCTTTGCGTTTGCACGCTG	qPCR
NETO1	TGCAGTGTGGAACTTGGACAA	TGGAGCGGCTTCTATGATGTAG	qPCR
ST18	CAAACCACCTAGAGTCCCAAAG	ACACCTGTTCTCACAAGGGATA	qPCR
CADPS	TCAGATGTCGTGCTGTCTTTC	TATACGATGCGATTTGGAGCC	qPCR
SYT4	ATGGGATACCCTACACCCAAAT	TCCCGAGAGAGGAATTAGAACTT	qPCR
RSU1	GCTGAGGACCAGTTGTGTGA	CGTCTTAGGGGCTACCTTCC	RT-PCR
RPH3AL	GCAAACCCACTGATCATTCC	TCCCAGTGATTCAGCTCCTC	RT-PCR
EFCAB13	GGAAAAGGAAATGCTGTCTAACC	ATCCCCAATATCCACCATGT	RT-PCR
OCT3/4 CDS	CCCCAGGGCCCCATTTTGGTACC	ACCTCAGTTTGAATGCATGGGAGAGC	qPCR
OCT3/4 pla	CATTCAAACTGAGGTAAGGG	TAGCGTAAAAGGAGCAACATAG	qPCR
KLF4 CDS	ACCCATCCTTCCTGCCCGATCAGA	TTGGTAATGGAGCGGCGGGACTTG	qPCR
KLF4 pla	CCACCTCGCCTTACACATGAAGA	TAGCGTAAAAGGAGCAACATAG	qPCR
SOX2 CDS	TTCACATGTCCCAGCACTACCAGA	TCACATGTGTGAGAGGGGCAGTGTGC	qPCR
SOX2 pla	TTCACATGTCCCAGCACTACCAGA	TTTGTTTGACAGGAGCGACAAT	qPCR
L-MYC CDS	GCGAACCCAAGACCCAGGCCTGCTCC	CAGGGGGTCTGCTCGCACCGTGATG	qPCR
L-MYC pla	GGCTGAGAAGAGGATGGCTAC	TTTGTTTGACAGGAGCGACAAT	qPCR
LIN28 CDS	AGCCATATGGTAGCCTCATGTCCGC	TCAATTCTGTGCCTCCGGGAGCAGGG TAGG	qPCR
LIN28 pla	AGCCATATGGTAGCCTCATGTCCGC	TAGCGTAAAAGGAGCAACATAG	qPCR
oriP	TTCCACGAGGGTAGTGAACC	TCGGGGGTGTTAGAGACAAC	genomic PCR
OCT4 endogenous	AGTTTGTGCCAGGGTTTTTG	ACTTCACCTTCCCTCCAACC	genomic PCR
BIP	TGTTCAACCAATTATCAGCAAACTC	TTCTGCTGTATCCTCTTCACCAGT	qPCR
CHOP	AGAACCAGGAAACGGAAACAGA	TCTCCTTCATGCGCTGCTTT	qPCR
spliced XBP-1	CTGAGTCCGCAGCAGGTGCAG	ATCCATGGGGAGATGTTCTGG	qPCR
CASP4	GAAACTCCAAGGGCCAAAGC	TCCATTTTCAATTGCCAGGAA	qPCR
GluR2	TCTGGTTTTCCTTGGGTGCC	AGATCCTCAGCACTTTCG	1st PCR
GluR2	GGTTTTCCTTGGGTGCCTTTAT	ATCCTCAGCACTTTCGATGG	nested PCR
ACTB	TGAAGTGTGACGTGGACATC	GGAGGAGCAATGATCTTGAT	PCR & qPCR

Supplemental Experimental Procedures

TALEN-directed mutagenesis

Platinum TALEN-expression vectors were constructed by modified Golden Gate-mediated method as previously described (Sakuma et al., 2013) with some modifications. Briefly, DNA-binding repeats were assembled with the two-step Golden Gate reaction using the Platinum Gate TALEN Kit (Addgene, Kit #1000000043). After the repeat assembly, the CMV promoter of the constructed TALEN vector was replaced with the EF1α promoter. . In order to target upstream region of splicing acceptor site of *FUS* intron 14, a pair of TALENs with following target sequence was designed and constructed (Figure S3A), 5'-TACTCGCTGGGTTAggaggggcagataggATATCTAGGCTTGGAGA-3' (Uppercase and underlined: Sequence binding to TALE-domain of TALENs, Uppercase without underline: T base binding to N-terminal domain of TALE, lowercase: spacer sequence cut by FokI-domain) and was used in farther experiment. In order to introduce H517D mutation in *FUS* exon 15 of control iPSC line, 409B2, a targeting donor plasmid with mutant exon 15 in 3' arm (Figure S3A) was constructed by Multisite Gateway-based method. Either 5' or 3' arm with appropriate *att*B signals at both ends and a *lox*P signal at either side was amplified by 2-step PCR using following PCR primers with additional recombination signals (oriented in the 5' to the 3' direction) and FALS patient genomic DNA with the mutant *FUS* gene as a template. For 5' arm, first with B3-5'FUS-Fw and LoxP-5'FUS-Rv, second with B3-5'FUS-Fw and B1r-LoxP-Rv. For 3' arm, first with PxoL-3'FUS-Fw and B4-3'FUS-Rv, second with B2r-PxoL-Fw and B4-3'FUS-Rv. Then the 5' and 3' arm fragments were cloned into pDONR P3-P1r (Sone et al., 2012) or pDONR-P2r-P4 (Sone et al., 2012), respectively by BP reaction. A positive-negative selection marker cassette, PGK-Puro^{R}-ΔTK (PurTK), was amplified by PCR using primers, B1-EcoRI-pPGK-F and B2-XbaI-PGKpA-R, and a plasmid vector, PB-TET-PH (kindly provided by Dr. Yuhki Nakatake, Keio University) as a template. Then it was cloned into pDONR201 (Life Technologies) by BP reaction. All the three fragments were confirmed by Sanger sequencing and assembled into pUC-DEST-R3R4 (Sone et al., 2012) by multisite LR reaction. Resulted plasmid clone, pUC-5'3'PDS-FUS(H517D) was used as the targeting donor plasmid (Figure S3A).

TALEN-mediated genome editing of iPSCs

For generating isogenic mutant lines, a control iPSC line, 409B2 was cultured under feeder-free condition using StemFit AK03 (Ajinomoto) and a recombinant laminin-511 E8 fragment, iMatrix-511 (Nippi) as previously described (Nakagawa et al., 2014). Cells were treated with Y27632 (Sigma) at 10 μM overnight before gene transfer. After being dissociated into single cells using TrypLESelect (Thermo Fisher), the cells were divided into 1×10^{6} cells and centrifuged at $200 \times g$ for 5 min. The supernatant was removed and the pellet was resuspended into 100 μl of Opti-MEM (Thermo Fisher) containing 10 μM Y27632, 5 μg each of left and right TALEN-expressing plasmids and 10 μg of targeting donor plasmid. Electroporation was performed using NEPA21 electroporator (Nepagene) with modifying the previously reported method (Li et al., 2015) with 275 V poring pulse voltage and 0.5 ms poring time. Cells were dispersed into 3 mL of StemFit AK03 containing 10 μM Y27632 and plated on an iMatrix-511-coated 6-cm dish (D=0). The medium was changed to StemFit AK03 without Y27632 every other day since the next day (D=1) until colony-picking. To obtain cells with homologous recombinant of PGK-PurTK into *FUS* locus, two times selection was performed with adding 1 μg/ml of puromycin to media for 24 h. Around D=14 to D=20, each single puromycin-resistant iPSC colony was picked up into 10 μl of TrypLESelect with Y27632 in one well of 96-well plate. After incubation for a few minutes at room temperature, 90μl of StemFit AK03 with Y27632 was added to the well and cells were dissociated roughly by pipetting and plated on one well of iMatrix-511-coated 24-well plate filled with 400 μl of StemFit AK03 with Y27632. The picked-up iPSCs were maintained in StemFit AK03 without Y27632 from the next day until the first passage. One quarter of the cells were passaged and the rest were harvested for preparing genomic DNA using KAPA Express Extract kit (Nippon Genetics). PCR genotyping for identification of knock-in clone was performed using following primers (oriented in the 5' to the 3' direction);
5'FUS-PCR-Fw and PGKP-Rv for detection of 5' border of knock-in allele and PuroR-Fw and 3'FUS-PCR-Rv for detection of 3' border of knock-in allele, 5'FUS-PCR-Fw and 3'FUS-PCR-Rv for detection of intact allele. The PCR fragments from knock-in clones were purified by PEG precipitation and confirmed for gene modification by Sanger sequencing using following primers; Seq-FUS-Fw and Seq-FUS-Rv. The established knock-in iPSC clones with successful gene modification was maintained by feeder-free method in 6-well plate.

Next, to obtain PGK-PurTK cassette free cells, the cells were infected with AdEFNCre-4FVF (kindly provided by Dr. Yumi Kanegae, the University of Tokyo), which is an EF-1α promoter derivative of a Cre recombinase expressing adenoviral vector, AxCANCre (Kanegae et al., 1995) (D=0). Two days after infection (D=2), 2.5 μg/ml of ganciclovir (Sigma) at final concentration was added to the media for selecting cells that is free from PGK-PurTK cassette flanked by two *lox*P signals. After selections (D=14 to D=20), each single ganciclovir-resistant iPSC colony was picked up as described above. PCR genotyping and Sanger sequencing of the PCR fragments were performed using the primers described above in order to confirm correct excision of the cassette and site-directed gene modification of H517D mutation derived from patient *FUS* gene.

RNA Editing Analysis

Analysis of RNA editing on AMPA receptor subunit GluA2 was performed as previously described with slight modifications (Kawahara et al., 2004; Nishimoto et al., 2008). Briefly, genomic DNA was amplified using 1st PCR primers, and nested PCR was conducted using the first PCR product as template. The nested PCR products were digested with BbvI restriction enzyme (New England Biolabs). The digested PCR products were analyzed using a Bioanalyzer 2100 (Agilent Technologies).

Supplemental References

Akiyama T, Warita H, Kato M, Nishiyama A, Izumi R, Ikeda C, Kamada M, Suzuki N, Aoki M. (2016). Genotype-phenotype relationships in familial ALS with FUS/TLS mutations in Japan. Muscle Nerve. doi: 10.1002/mus.25061.

Kanegae, Y., Lee, G., Sato, Y., Tanaka, M., Nakai, M., Sakaki, T., Sugano, S., and Saito, I. (1995). Efficient gene activation in mammalian cells by using recombinant adenovirus expressing site-specific Cre recombinase. Nucleic Acids Res 23, 3816-3821.

Li, H. L., Fujimoto, N., Sasakawa, N., Shirai, S., Ohkame, T., Sakuma, T., Tanaka, M., Amano, N., Watanabe, A., Sakurai, H., et al. (2015). Precise correction of the dystrophin gene in duchenne muscular dystrophy patient induced pluripotent stem cells by TALEN and CRISPR-Cas9. Stem Cell Reports 4, 143-154.

Nakagawa, M., Taniguchi, Y., Senda, S., Takizawa, N., Ichisaka, T., Asano, K., Morizane, A., Doi, D., Takahashi, J., Nishizawa, M., et al. (2014). A novel efficient feeder-free culture system for the derivation of human induced pluripotent stem cells. Sci Rep 4, 3594.

Nishimoto, Y., Yamashita, T., Hideyama, T., Tsuji, S., Suzuki, N., and Kwak, S. (2008). Determination of editors at the novel A-to-I editing positions. Neurosci. Res. *61*, 201–206.

Sakuma, T., Ochiai, H., Kaneko, T., Mashimo, T., Tokumasu, D., Sakane, Y., Suzuki, K., Miyamoto, T., Sakamoto, N., Matsuura, S., and Yamamoto, T. (2013). Repeating pattern of non-RVD variations in DNA-binding modules enhances TALEN activity. Sci Rep 3, 3379.

Sone, T., and Imamoto, F. (2012). Methods for constructing clones for protein expression in mammalian cells. Methods Mol Biol 801, 227-250.
